# Supplementary material for: Functional Network Mapping Reveals State-Dependent Response to IGF1 Treatment in Rett Syndrome
Source: Brain Sci. 2020 Aug 3;10(8):515. doi: 10.3390/brainsci10080515 (PMC7465931; doi:10.3390/brainsci10080515)
Supplement: Supplementary file 1 [file brainsci-10-00515-s001.pdf]

Supplementary Materials

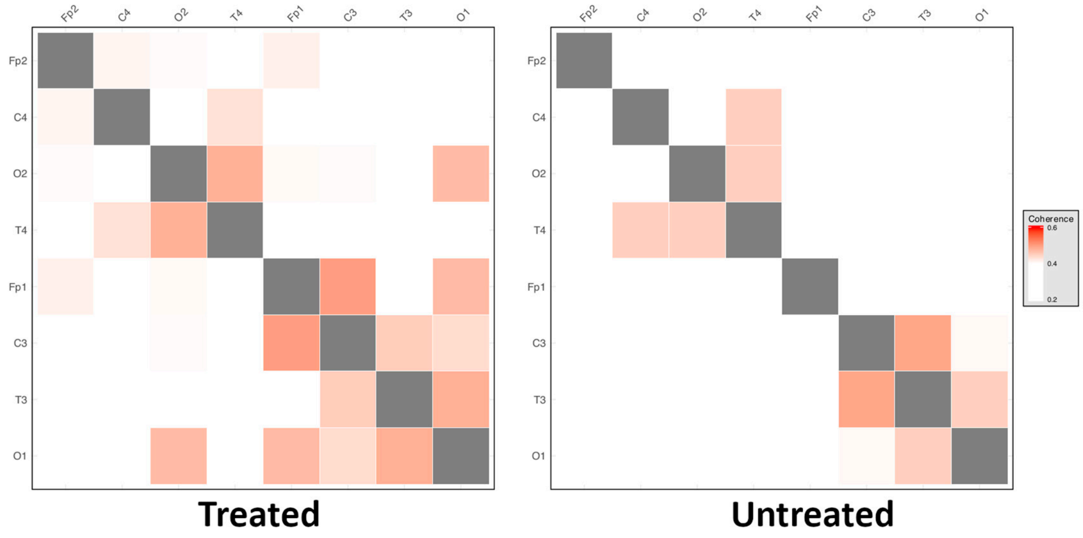

Figure S1. Heatmap demonstrating distribution of coherence between individual electrode pairs, treated vs. untreated at baseline. There are statistically significant differences between a number of long-range electrode pairs, suggesting differences in network connectivity at baseline.

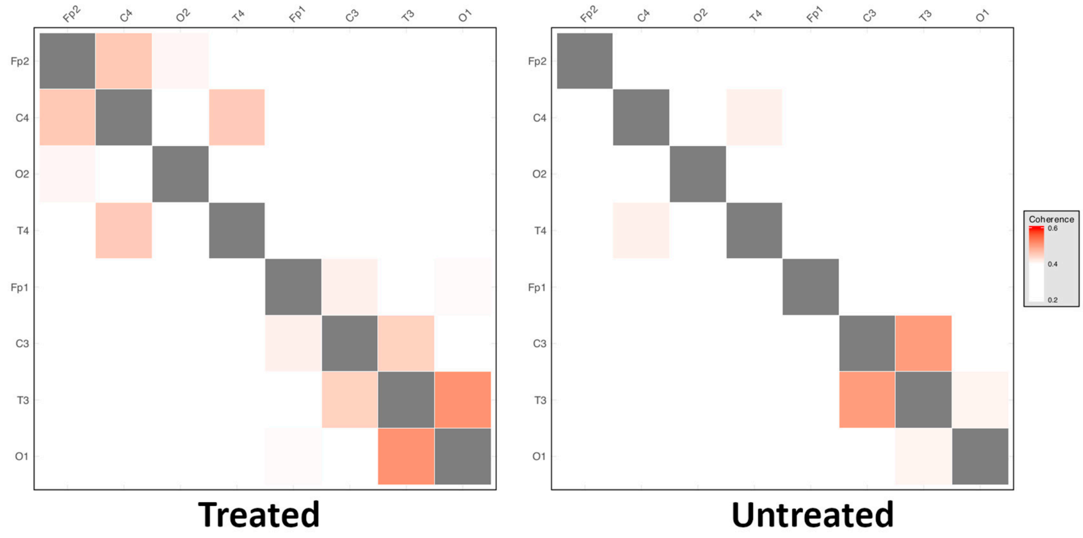

Figure S2. Heatmap demonstrating distribution of coherence between individual electrode pairs, treated vs, untreated at twelve months. There were no statistically significant differences between any individual electrode pairs, suggesting a change in connectivity following treatment.

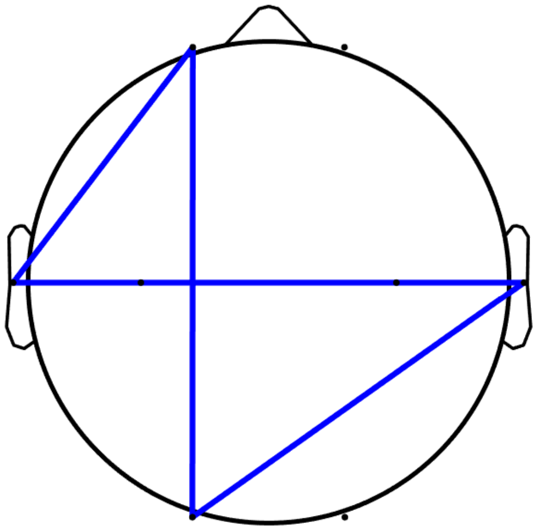

Figure S3. Schematic of differences network architecture at baseline, treated vs. untreated groups. Lines join channels with a statistically significant difference in coherence between groups at baseline ($p < 0.05$). The differences in network features were predominantly driven by long-range connections.

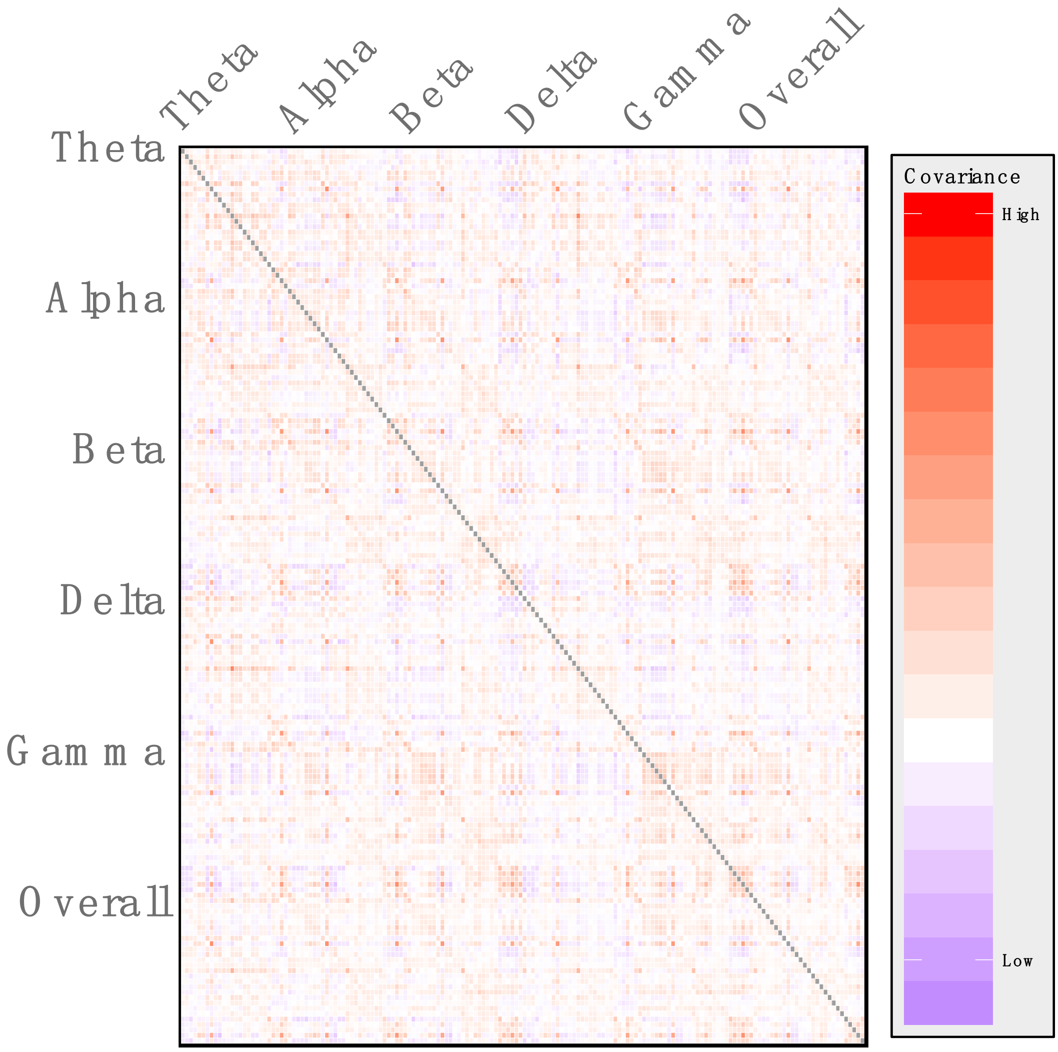

Figure S4. Covariance matrix of network measures, treated patients at baseline. There were statistically significant differences in network loadings at baseline between treated and untreated groups, suggesting that network-level differences may exist that were not apparent on clinical assessment. This may have implications for targeting treatment.

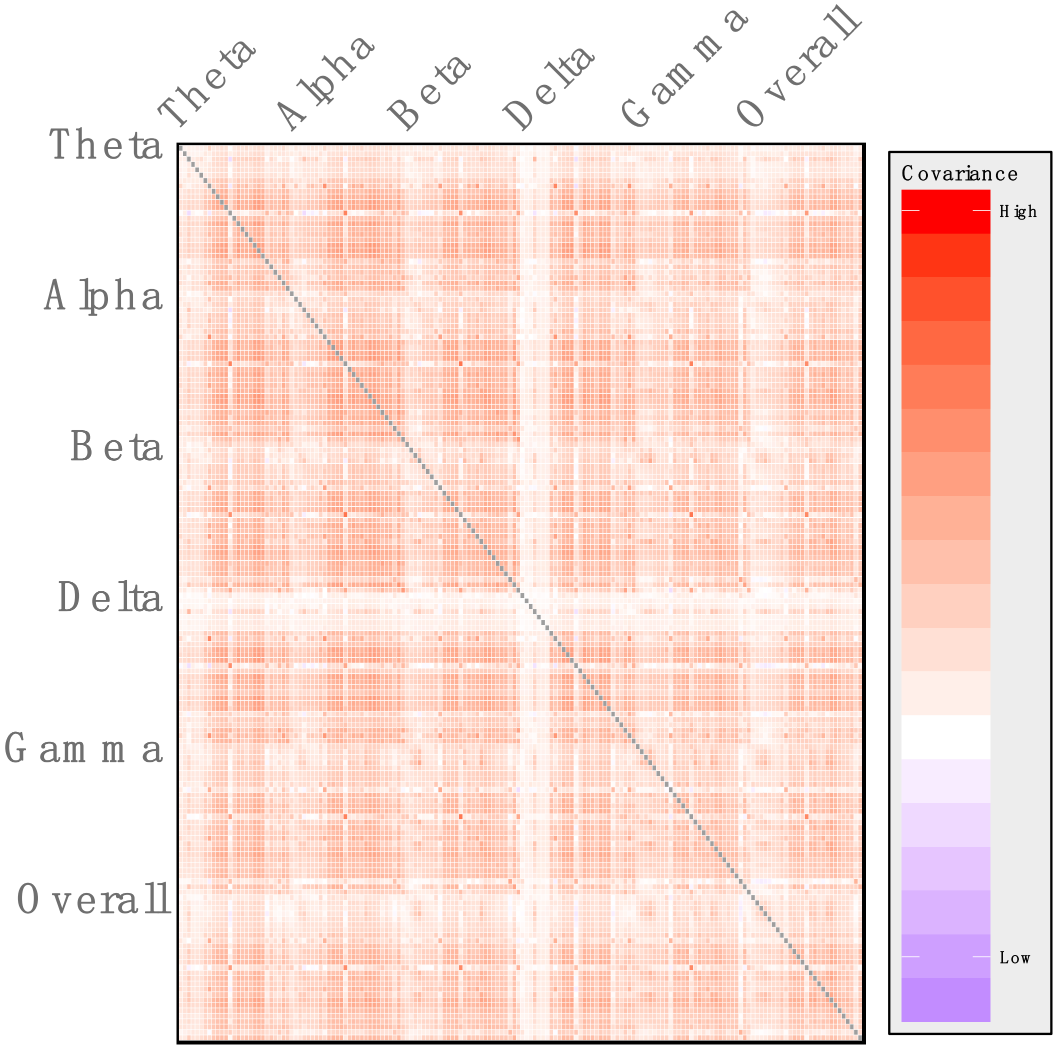

Figure S5. Covariance matrix of network measures, untreated patients at baseline. There were statistically significant differences in network loadings at baseline between treated and untreated groups, suggesting that network-level differences may exist that were not apparent on clinical assessment. This may have implications for targeting treatment.

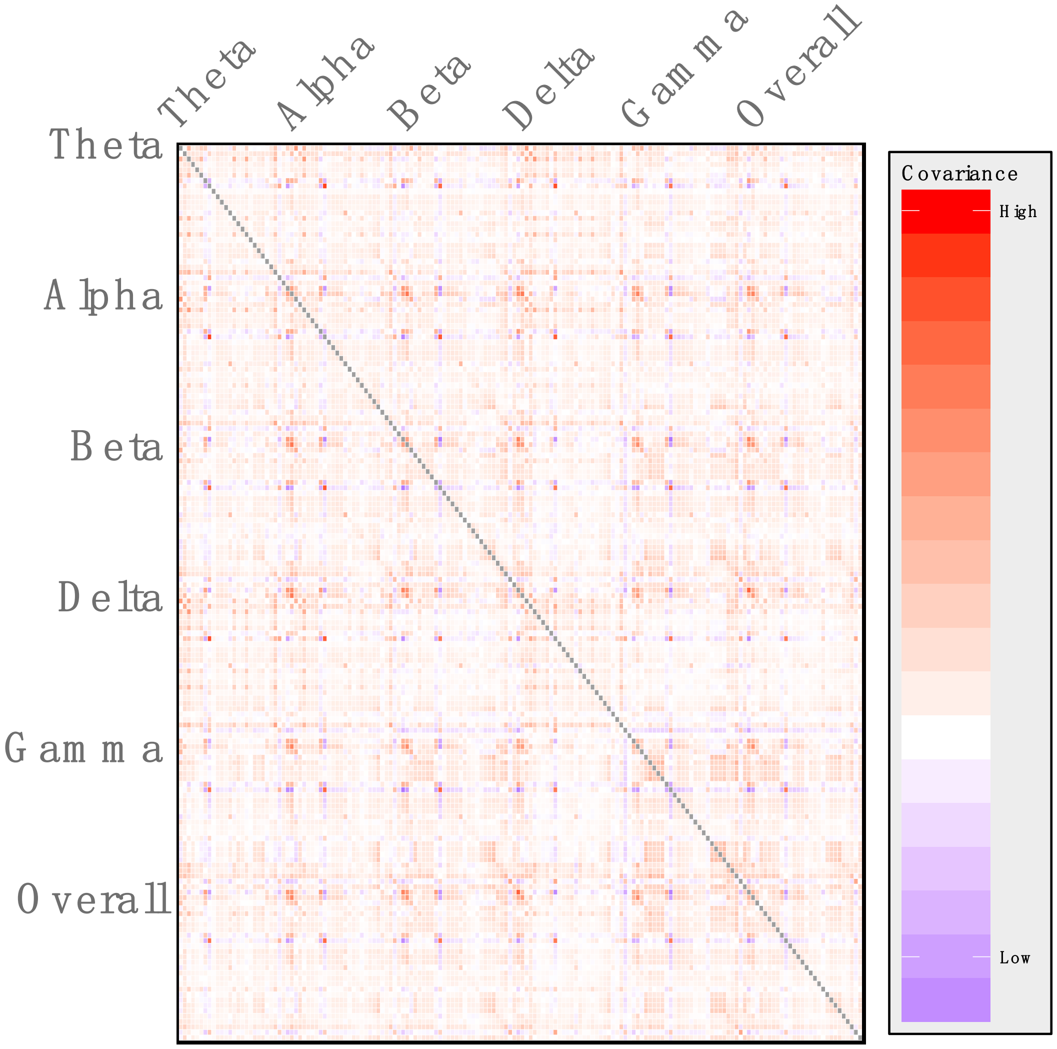

Figure S6. Covariance matrix of network measures, treated patients at 12 months. There was a statistically significant effect of treatment group & time on network loadings, suggesting that treatment with IGF-1 caused alterations in cortical electrophysiology. This underlines the emerging importance of network electrophysiology in Rett Syndrome and its treatment and may have implications for targeting treatment based on electrophysiological biomarkers.

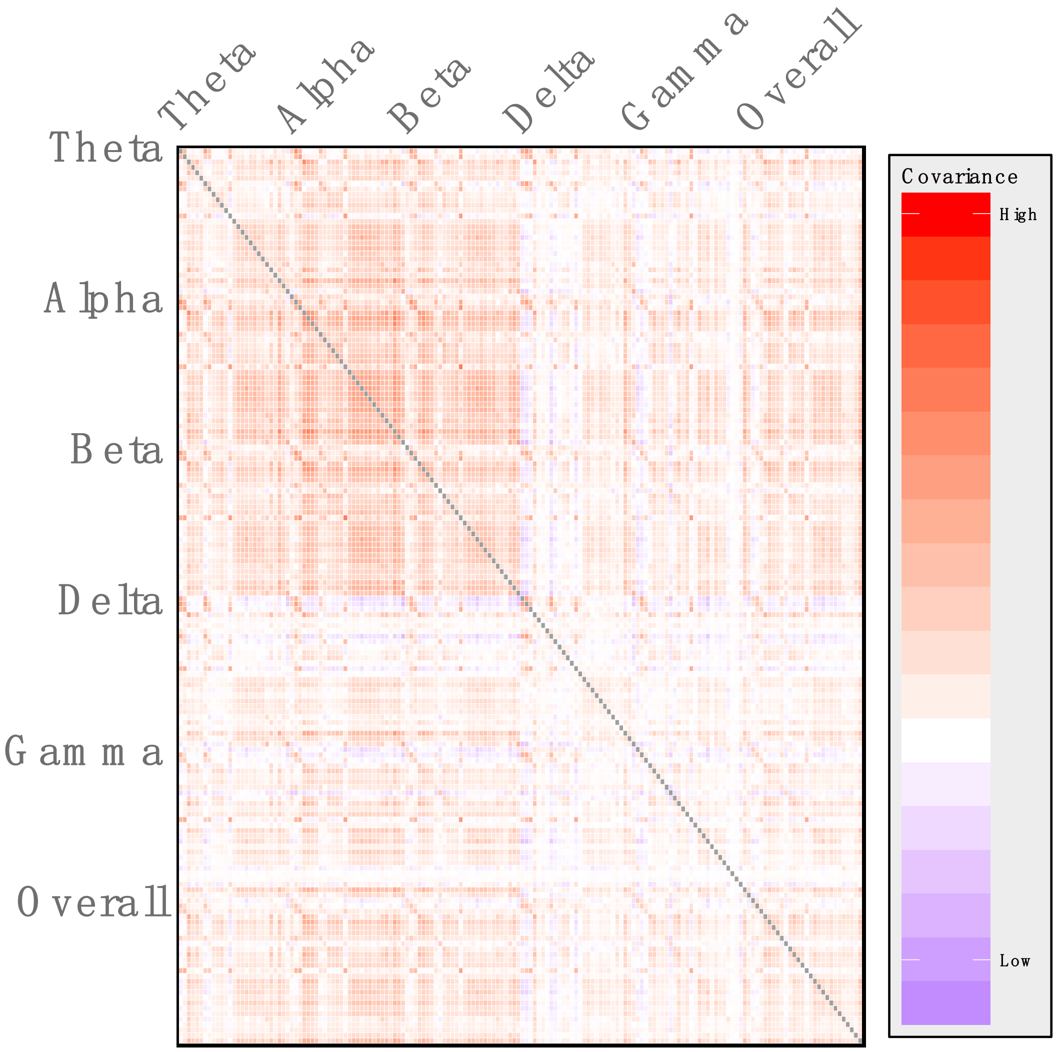

Figure S7. Covariance matrix of network measures, untreated patients at 12 months. There was a statistically significant effect of treatment group & time on network loadings, suggesting that treatment with IGF-1 caused alterations in cortical electrophysiology. This underlines the emerging importance of network electrophysiology in Rett Syndrome and its treatment and may have implications for targeting treatment based on electrophysiological biomarkers.

.

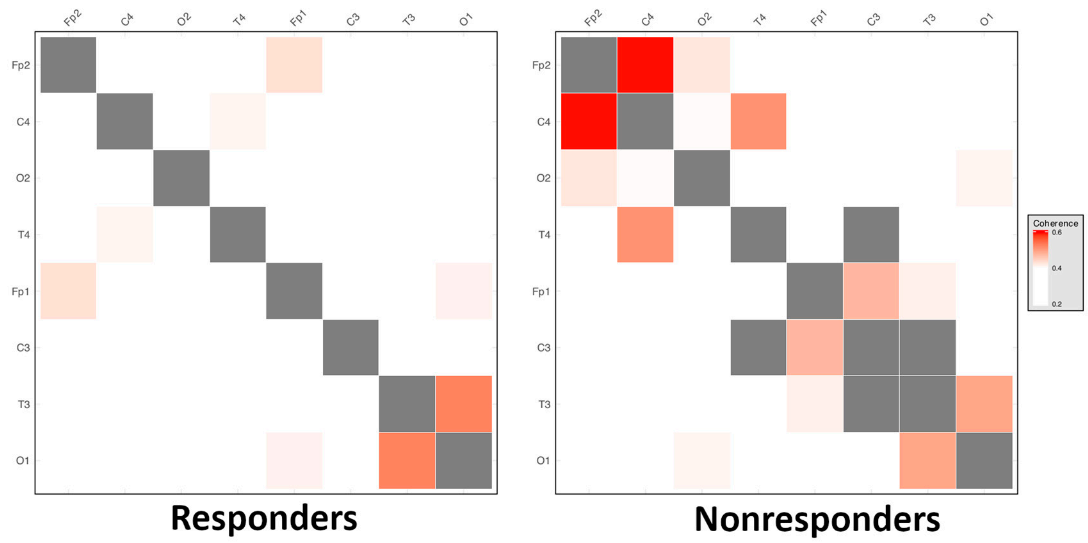

Figure S8. Heatmap demonstrating the distribution of coherence measures at individual electrode pairs, responders vs. nonresponders at twelve months. There were no statistically significant differences in coherence between individual electrodes, suggesting that observed differences in overall network architecture are due to an overall pattern change, rather than differences at individual channel pairs.

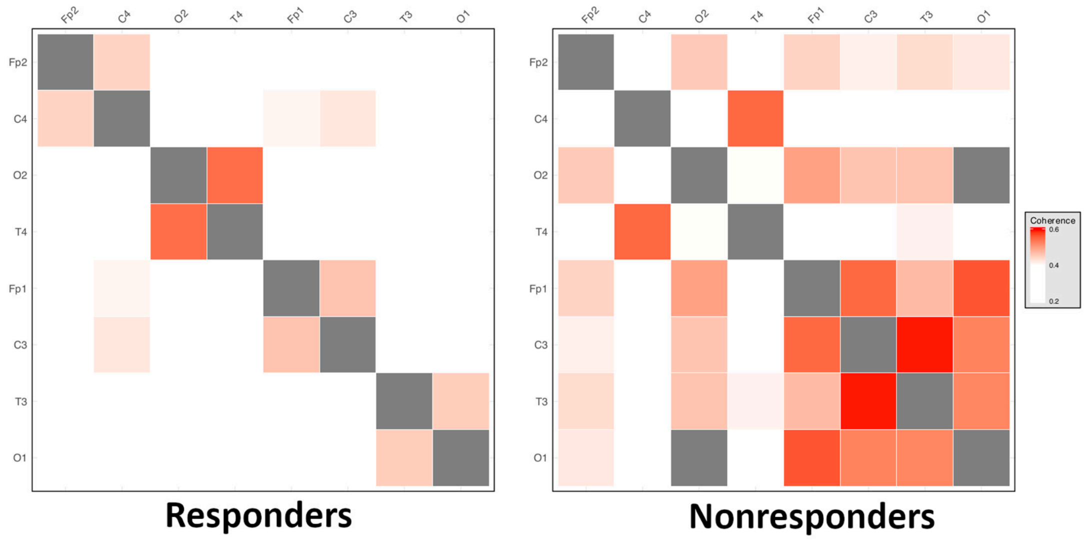

Figure S9. Heatmap demonstrating coherence measures at individual electrode pairs, responders vs. nonresponders at baseline. There are a number of statistically significant differences between measures at baseline, suggesting network-level differences between responders and non-responders prior to treatment. These are predominantly long-range pairs.

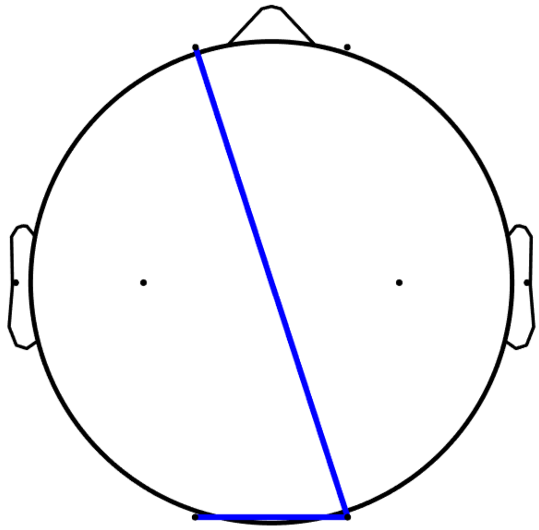

Figure S10. Schematic of differences network architecture at baseline, responders vs. nonresponders. Lines join channels with a statistically significant difference in coherence between groups at baseline ($p < 0.05$). The differences in network features were predominantly driven by long-range and occipital interhemispheric connections.

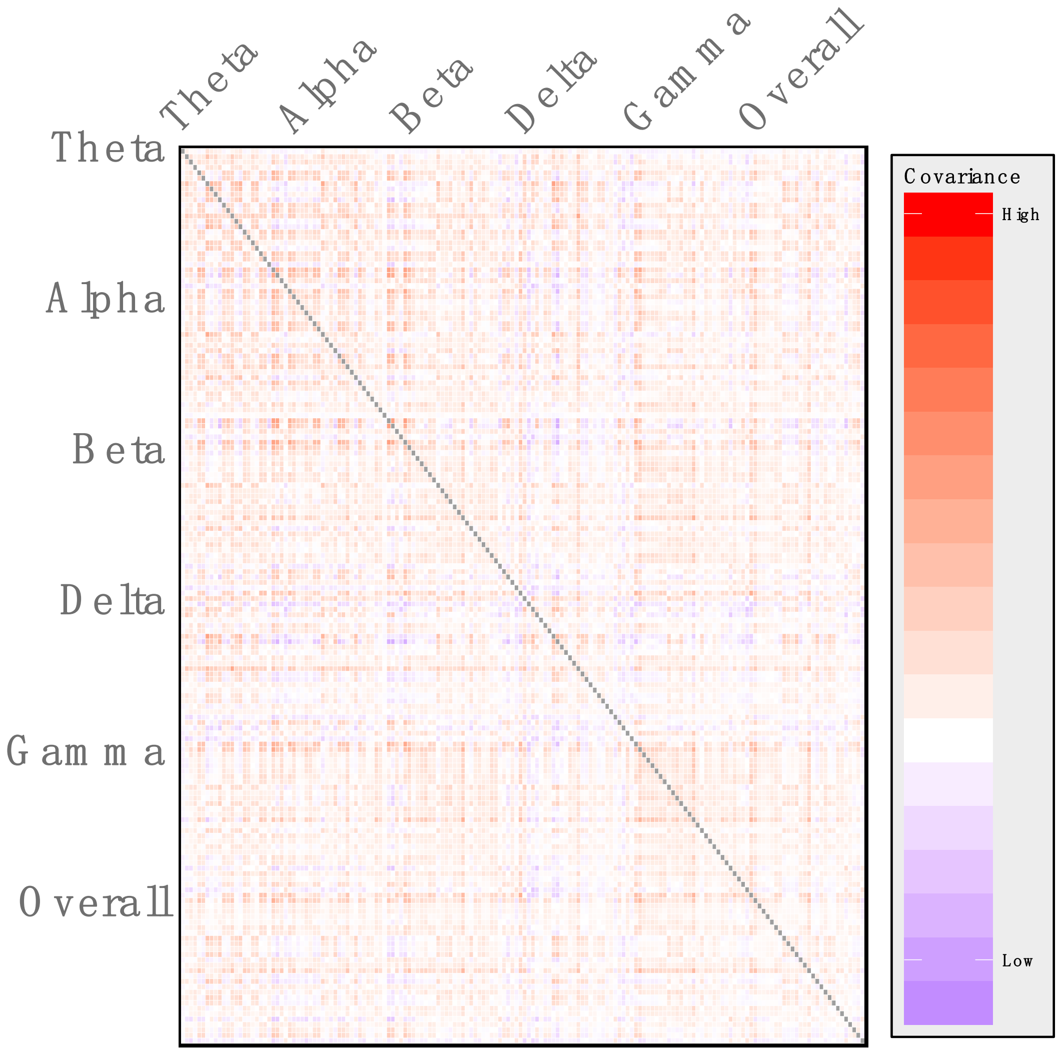

Figure S11. Covariance matrix of network measures, responders at baseline. There were statistically significant differences in network loadings at baseline between responders and nonresponders, suggesting that network-level differences may identify those likely to benefit before treatment is initiated. This may have implications for targeting treatment.

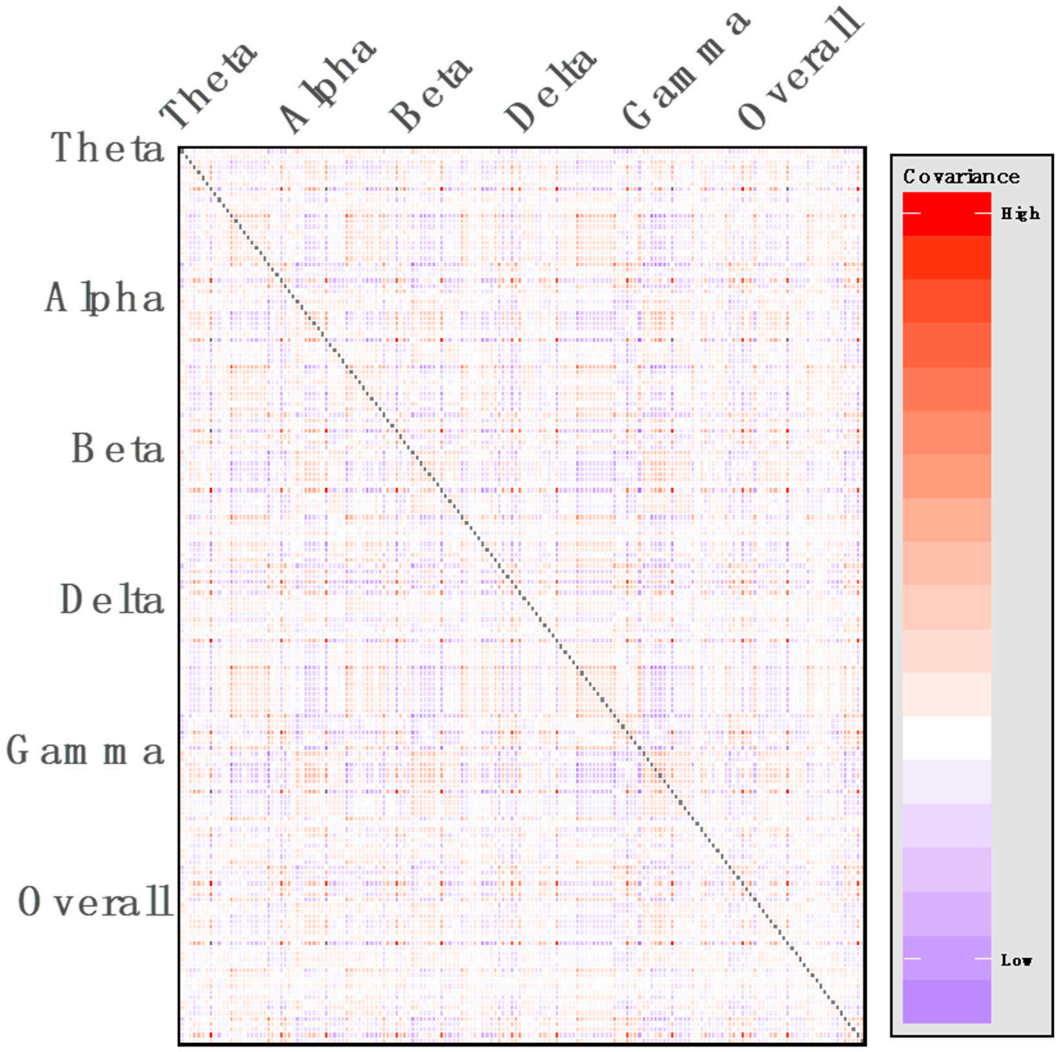

Figure 12. Covariance matrix of network measures, nonresponders at baseline. There were statistically significant differences in network loadings at baseline between responders and nonresponders, suggesting that network-level differences may identify those likely to benefit before treatment is initiated. This may have implications for targeting treatment.

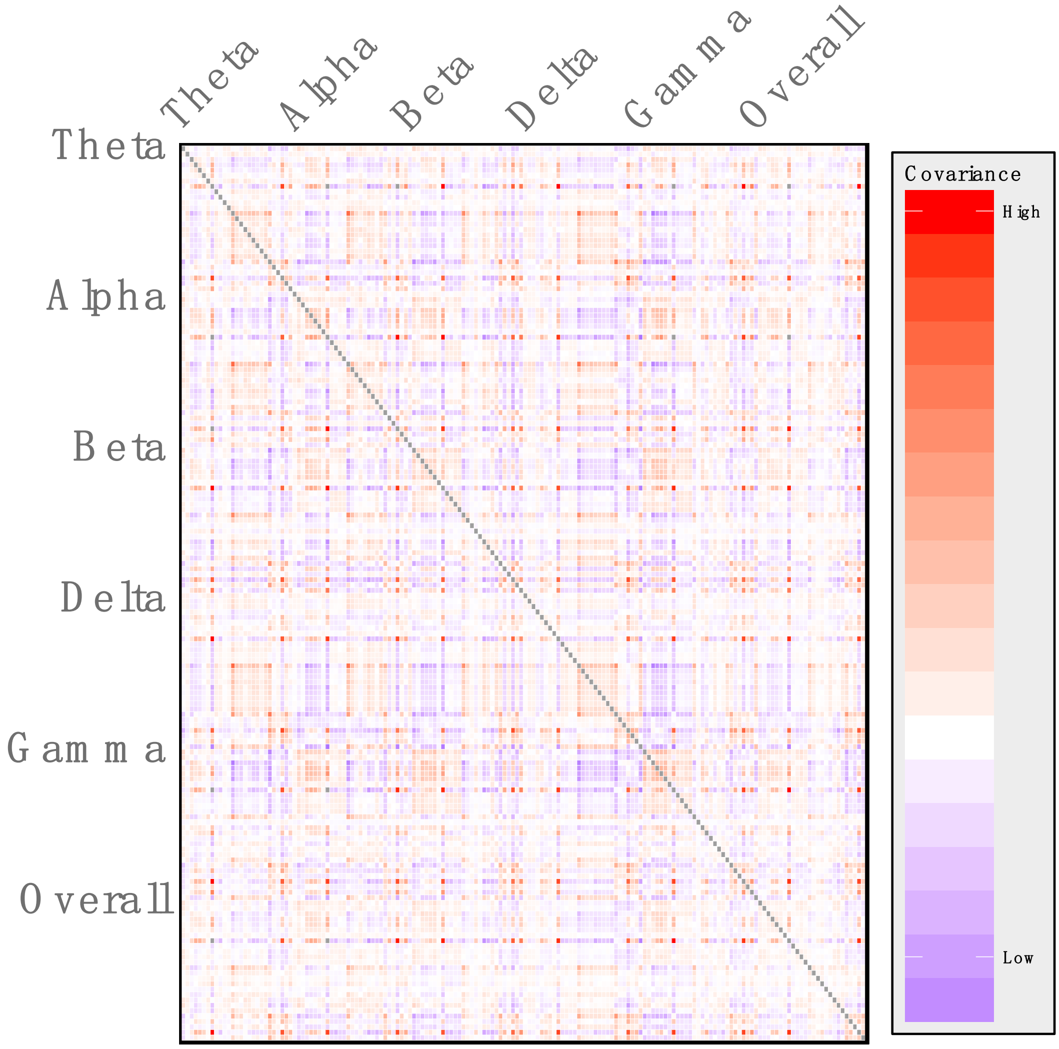
Theta
Alpha
Beta
Delta
Gamma
Overall
Theta
Alpha
Beta
Delta
Gamma
Overall
Covariance
High
Low

.

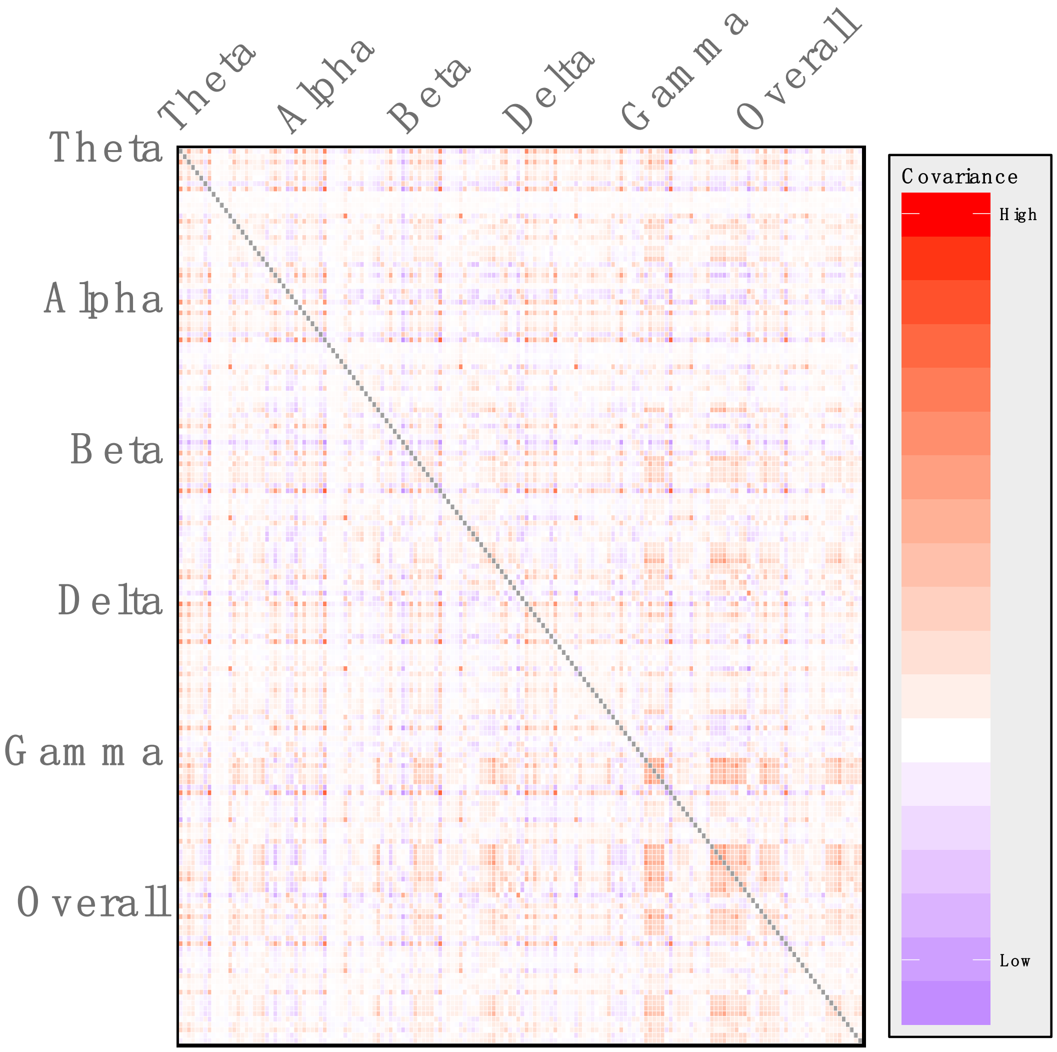

Figure S13. Covariance matrix of network measures, responders at 12 months. There was a statistically significant effect of treatment group & time on network loadings, suggesting that there were specific alterations in cortical electrophysiology in those that responded clinically to treatment with IGF-1. This underlines the emerging importance of network electrophysiology in Rett Syndrome and its treatment and may have implications for targeting treatment based on electrophysiological biomarkers.

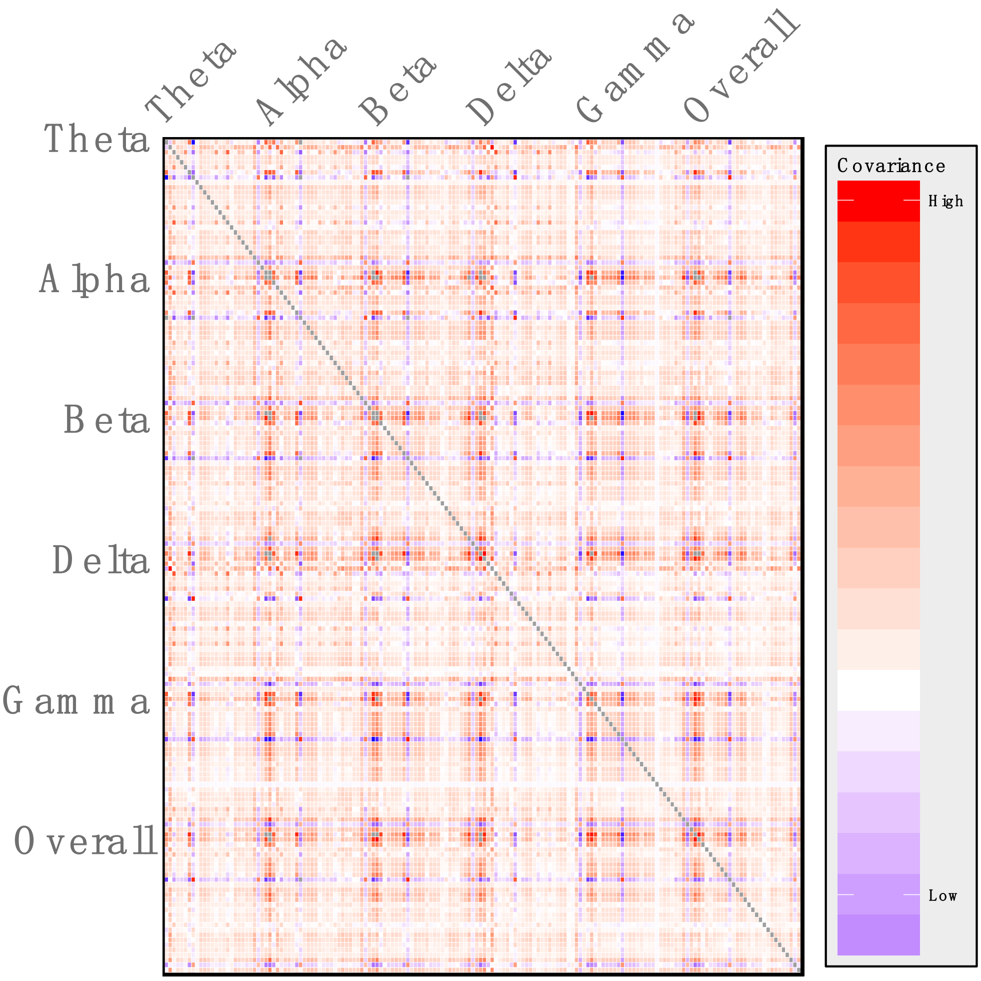

Figure S14. Covariance matrix of network measures, nonresponders at 12 months. There was a statistically significant effect of treatment group & time on network loadings, suggesting that there were specific alterations in cortical electrophysiology in those that responded clinically to treatment with IGF-1. This underlines the emerging importance of network electrophysiology in Rett Syndrome and its treatment and may have implications for targeting treatment based on electrophysiological biomarkers

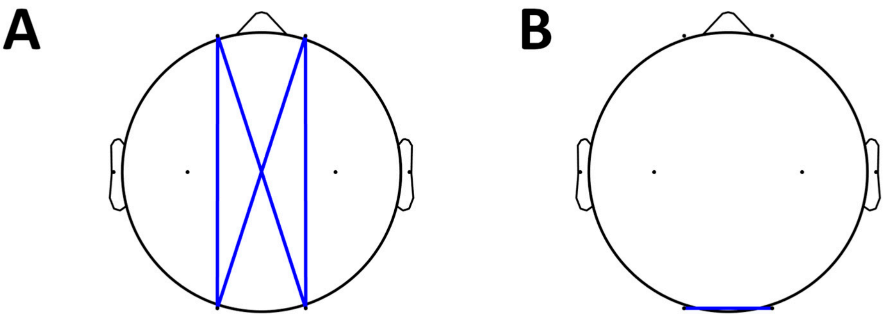

Figure S15. Schematic of channels included in prediction model. (A) shows channel pairs included in long-range coherence measure, (B) demonstrates the occipital interhemispheric channel. These channels showed a promising ability to predict treatment response to IGF-1.

Table S1. Overall power and power within each band at each electrode location, treated vs. untreated at baseline.

Band	Treated	Untreated	*p*
Fp2 Theta	-47.5507 +/- 143.8417'	-1.6147 +/- 6.5254'	0.9047619048
Fp2 Alpha	-41.4767 +/- 127.0681'	-1.1731 +/- 5.0021'	0.9047619048
Fp2 Beta	-19.384 +/- 62.4975'	-1.4207 +/- 4.1829'	1
Fp2 Delta	-51.7872 +/- 158.3624'	-2.4492 +/- 8.624'	0.9047619048
Fp2 Gamma	13.649 +/- 34.4742'	0.73436 +/- 0.55074'	0.5555555556
Fp2 Overall	-1.9291 +/- 4.3741'	-3.0949 +/- 4.1132'	0.5555555556
C4 Theta	-5.2122 +/- 49.3782'	-2.1315 +/- 16.3853'	0.5555555556
C4 Alpha	-5.6285 +/- 41.5282'	-1.382 +/- 12.4684'	0.4126984127
C4 Beta	-3.1068 +/- 24.7323'	-1.2585 +/- 9.5609'	0.5555555556
C4 Delta	-3.8606 +/- 60.0226'	-3.0252 +/- 20.1411'	0.5555555556
C4 Gamma	2.2582 +/- 10.6184'	1.1893 +/- 1.09'	0.1904761905
C4 Overall	1.0284 +/- 5.1993'	-3.2215 +/- 4.8507'	0.4126984127
T4 Theta	-0.92801 +/- 9.2063'	2.5536 +/- 13.1519'	0.5555555556
T4 Alpha	-0.6639 +/- 7.2729'	1.9504 +/- 10.1534'	0.5555555556
T4 Beta	0.029378 +/- 4.6261'	1.4846 +/- 8.3281'	0.5555555556
T4 Delta	-1.0907 +/- 11.2634'	2.7077 +/- 17.2825'	0.5555555556
T4 Gamma	1.3624 +/- 1.6974'	0.98807 +/- 0.5479'	0.7301587302
T4 Overall	3.0692 +/- 6.5926'	-0.54291 +/- 3.8246'	0.4126984127
O2 Theta	-13.2068 +/- 24.1104'	4.0086 +/- 8.1257'	0.06349206349
O2 Alpha	-10.7554 +/- 20.4886'	3.1333 +/- 6.0662'	0.06349206349
O2 Beta	-6.3833 +/- 12.8202'	2.3972 +/- 4.4152'	0.06349206349
O2 Delta	-15.3746 +/- 26.8835'	4.6939 +/- 10.4838'	0.06349206349
O2 Gamma	3.1233 +/- 4.8017'	0.93962 +/- 0.45299'	0.5555555556
O2 Overall	1.1752 +/- 6.4051'	-2.5319 +/- 4.6894'	0.9047619048
Fp1 Theta	-6.4613 +/- 11.7724'	4.2463 +/- 18.4689'	0.9047619048
Fp1 Alpha	-5.2494 +/- 9.6817'	3.7944 +/- 14.4406'	0.9047619048
Fp1 Beta	-2.5847 +/- 6.0835'	3.5336 +/- 13.0959'	0.9047619048
Fp1 Delta	-7.7167 +/- 14.3276'	5.173 +/- 25.3602'	0.9047619048
Fp1 Gamma	2.6713 +/- 2.3099'	1.5986 +/- 2.0775'	1
Fp1 Overall	-1.752 +/- 4.421'	-2.5234 +/- 3.632'	0.7301587302
C3 Theta	9.6325 +/- 21.8476'	-17.3424 +/- 27.7711'	1
C3 Alpha	7.3295 +/- 15.4487'	-12.9029 +/- 20.9959'	1
C3 Beta	6.7814 +/- 14.3278'	-11.1367 +/- 18.3301'	1
C3 Delta	11.1757 +/- 26.4174'	-25.7377 +/- 40.7283'	1
C3 Gamma	1.1272 +/- 1.6614'	0.35489 +/- 1.2033'	1
C3 Overall	1.4238 +/- 4.2144'	-2.354 +/- 4.9664'	0.1111111111
T3 Theta	20.8459 +/- 54.9947'	9.3832 +/- 17.7964'	0.7301587302
T3 Alpha	16.5229 +/- 42.9367'	7.8688 +/- 14.4523'	0.7301587302
T3 Beta	11.5948 +/- 28.738'	7.0127 +/- 12.8476'	0.7301587302
T3 Delta	25.4483 +/- 68.4991'	12.5056 +/- 25.2996'	0.9047619048

T3 Gamma	-0.94198 +/- 6.1223'	1.0116 +/- 0.68677'	1
T3 Overall	3.3901 +/- 4.859'	-0.87694 +/- 4.5616'	0.2857142857
O1 Theta	-2.7495 +/- 17.1427'	-4.4477 +/- 12.5136'	0.1111111111
O1 Alpha	-2.0957 +/- 13.9568'	-3.2576 +/- 9.8101'	0.1111111111
O1 Beta	-1.226 +/- 9.5359'	-2.4238 +/- 7.511'	0.1111111111
O1 Delta	-3.1572 +/- 18.874'	-6.4055 +/- 15.8787'	0.1111111111
O1 Gamma	1.084 +/- 1.2943'	1.0067 +/- 0.37125'	1
O1 Overall	1.353 +/- 4.6689'	-3.0215 +/- 4.7714'	1
R hemi Theta	-16.7244 +/- 53.0667'	0.70404 +/- 9.1051'	0.9047619048
R hemi Alpha	-14.6311 +/- 46.6077'	0.6322 +/- 6.8814'	0.9047619048
R hemi Beta	-7.2112 +/- 24.3645'	0.30059 +/- 5.3654'	0.9047619048
R hemi Delta	-18.0279 +/- 59.2178'	0.48182 +/- 11.7749'	0.7301587302
R hemi Gamma	5.0982 +/- 12.4531'	0.96284 +/- 0.4775'	0.1111111111
R hemi Overall	0.83584 +/- 5.2025'	-2.3478 +/- 4.093'	0.7301587302
L hemi Theta	5.317 +/- 16.0133'	-2.0402 +/- 9.5772'	0.2857142857
L hemi Alpha	4.1268 +/- 12.4357'	-1.1244 +/- 7.1953'	0.2857142857
L hemi Beta	3.6414 +/- 8.6745'	-0.75354 +/- 5.8077'	0.2857142857
L hemi Delta	6.4374 +/- 19.631'	-3.616 +/- 13.4631'	0.4126984127
L hemi Gamma	0.98516 +/- 1.7924'	0.99295 +/- 0.65462'	0.5555555556
L hemi Overall	1.1037 +/- 4.0083'	-2.194 +/- 4.1027'	0.4126984127
Overall Theta	-5.7037 +/- 28.2429'	-0.66814 +/- 5.6427'	0.1904761905
Overall Alpha	-5.2521 +/- 24.6106'	-0.24609 +/- 4.183'	0.1904761905
Overall Beta	-1.7849 +/- 13.3215'	-0.22646 +/- 3.1596'	0.1111111111
Overall Delta	-5.7954 +/- 31.7381'	-1.5671 +/- 8.115'	0.2857142857
Overall Gamma	3.0417 +/- 6.3182'	0.97789 +/- 0.45065'	0.5555555556
Overall Overall	0.96979 +/- 4.4172'	-2.2709 +/- 4.0148'	0.7301587302

Table S2. Hemispheric asymmetry, overall and within each band, untreated vs. treated at baseline.

Band	Treated	Untreated	*p*
Frontal Theta	41.0888 +/- 137.9382'	5.8608 +/- 21.203'	0.9047619048
Frontal Alpha	36.2265 +/- 121.5779'	4.9675 +/- 16.5359'	0.9047619048
Frontal Beta	16.7993 +/- 60.2644'	4.9543 +/- 14.8929'	0.9047619048
Frontal Delta	44.0713 +/- 152.0418'	7.6225 +/- 28.7263'	0.7301587302
Frontal Gamma	-10.9776 +/- 32.5008'	0.86421 +/- 2.1103'	0.7301587302
Frontal Overall	0.17714 +/- 2.2727'	0.57149 +/- 2.4073'	1
Parietal Theta	14.8442 +/- 59.4537'	-15.2109 +/- 36.0135'	0.7301587302
Parietal Alpha	12.957 +/- 48.2804'	-11.5209 +/- 27.2849'	0.5555555556
Parietal Beta	9.8882 +/- 30.8952'	-9.8781 +/- 22.7982'	0.7301587302
Parietal Delta	15.0364 +/- 72.5403'	-22.7124 +/- 49.1711'	0.7301587302
Parietal Gamma	-1.1309 +/- 11.8212'	-0.83442 +/- 1.4105'	0.9047619048
Parietal Overall	0.39547 +/- 3.4069'	0.8676 +/- 2.445'	0.4126984127
Temporal Theta	21.7736 +/- 57.8009'	6.8296 +/- 16.1171'	0.2857142857
Temporal Alpha	17.1863 +/- 45.0771'	5.9183 +/- 13.4189'	0.2857142857

Temporal Beta	11.5654 +/- 30.2151'	5.5281 +/- 12.6068'	0.2857142857
Temporal Delta	26.5389 +/- 72.192'	9.7977 +/- 23.7658'	0.2857142857
Temporal Gamma	-2.3044 +/- 6.4987'	0.023562 +/- 0.53226'	1
Temporal Overall	0.32091 +/- 4.4997'	-0.33404 +/- 2.4852'	0.4126984127
Occipital Theta	10.4573 +/- 23.8274'	-8.4563 +/- 17.5338'	0.9047619048
Occipital Alpha	8.6597 +/- 20.5463'	-6.3909 +/- 13.3715'	1
Occipital Beta	5.1574 +/- 11.471'	-4.821 +/- 10.1408'	0.7301587302
Occipital Delta	12.2173 +/- 26.5856'	-11.0994 +/- 22.6268'	0.9047619048
Occipital Gamma	-2.0393 +/- 5.6575'	0.067086 +/- 0.3357'	0.9047619048
Occipital Overall	0.17781 +/- 3.3702'	-0.48964 +/- 2.4748'	0.7301587302
Overall Theta	88.1648 +/- 217.4111'	-10.9771 +/- 59.585'	0.5555555556
Overall Alpha	75.0309 +/- 188.9366'	-7.026 +/- 45.3005'	0.5555555556
Overall Beta	43.4108 +/- 100.2333'	-4.2166 +/- 36.9007'	0.5555555556
Overall Delta	97.8631 +/- 245.1246'	-16.3921 +/- 77.6038'	0.5555555556
Overall Gamma	-16.4525 +/- 50.1066'	0.12043 +/- 2.8304'	0.2857142857
Overall Overall	1.0714 +/- 11.4666'	0.61544 +/- 6.5684'	0.9047619048

Table S3. Inter-electrode coherence measures in the overall spectrum, untreated vs. treated at baseline.

Band	Treated	Untreated	*p*
Fp2-C4 Theta	0.39488 +/- 0.19929'	0.27081 +/- 0.13146'	0.5555555556
Fp2-O2 Theta	0.43856 +/- 0.11658'	0.2562 +/- 0.11246'	0.7301587302
Fp2-T4 Theta	0.3787 +/- 0.15994'	0.21967 +/- 0.10383'	0.7301587302
Fp2-Fp1 Theta	0.46204 +/- 0.16753'	0.32464 +/- 0.21667'	0.5555555556
Fp2-C3 Theta	0.34263 +/- 0.14834'	0.22099 +/- 0.11575'	0.9047619048
Fp2-T3 Theta	0.37034 +/- 0.15419'	0.22343 +/- 0.11094'	0.4126984127
Fp2-O1 Theta	0.35844 +/- 0.13733'	0.22965 +/- 0.11807'	0.2857142857
C4-O2 Theta	0.38617 +/- 0.22401'	0.24777 +/- 0.16256'	0.9047619048
C4-T4 Theta	0.47847 +/- 0.28006'	0.36583 +/- 0.27638'	0.5555555556
C4-Fp1 Theta	0.37821 +/- 0.17156'	0.22879 +/- 0.20095'	0.03174603175
C4-C3 Theta	0.37856 +/- 0.1808'	0.26036 +/- 0.22457'	0.4126984127
C4-T3 Theta	0.3059 +/- 0.12841'	0.24425 +/- 0.23585'	0.9047619048
C4-O1 Theta	0.31504 +/- 0.12947'	0.23397 +/- 0.24084'	0.2857142857
O2-T4 Theta	0.52955 +/- 0.27429'	0.48706 +/- 0.28564'	0.4126984127
O2-Fp1 Theta	0.39486 +/- 0.14794'	0.23392 +/- 0.17623'	0.7301587302
O2-C3 Theta	0.35104 +/- 0.17727'	0.25166 +/- 0.23694'	0.9047619048
O2-T3 Theta	0.33102 +/- 0.15356'	0.25506 +/- 0.2'	0.7301587302
O2-O1 Theta	0.3838 +/- 0.14064'	0.29043 +/- 0.21475'	1
T4-Fp1 Theta	0.37197 +/- 0.15796'	0.21943 +/- 0.20157'	0.1904761905
T4-C3 Theta	0.33391 +/- 0.14912'	0.24151 +/- 0.24867'	0.4126984127
T4-T3 Theta	0.34288 +/- 0.1397'	0.25456 +/- 0.25251'	0.9047619048
T4-O1 Theta	0.33074 +/- 0.15777'	0.23896 +/- 0.24031'	0.9047619048
Fp1-C3 Theta	0.46992 +/- 0.20448'	0.40263 +/- 0.23171'	1

Fp1-T3 Theta	0.41061 +/- 0.19929'	0.30963 +/- 0.18596'	0.2857142857
Fp1-O1 Theta	0.45349 +/- 0.16876'	0.35818 +/- 0.18605'	0.7301587302
C3-T3 Theta	0.3873 +/- 0.24969'	0.41834 +/- 0.24009'	0.06349206349
C3-O1 Theta	0.37202 +/- 0.16157'	0.35346 +/- 0.23653'	0.9047619048
T3-O1 Theta	0.56078 +/- 0.20873'	0.39065 +/- 0.2298'	0.5555555556
Fp2-C4 Alpha	0.36294 +/- 0.16378'	0.30604 +/- 0.1634'	0.5555555556
Fp2-O2 Alpha	0.39244 +/- 0.13891'	0.31621 +/- 0.15267'	0.7301587302
Fp2-T4 Alpha	0.33948 +/- 0.11008'	0.26776 +/- 0.15927'	0.9047619048
Fp2-Fp1 Alpha	0.45438 +/- 0.18'	0.36291 +/- 0.19476'	0.5555555556
Fp2-C3 Alpha	0.3905 +/- 0.14723'	0.26308 +/- 0.17089'	0.7301587302
Fp2-T3 Alpha	0.3966 +/- 0.16994'	0.26215 +/- 0.17566'	0.4126984127
Fp2-O1 Alpha	0.37884 +/- 0.16095'	0.25868 +/- 0.17987'	0.2857142857
C4-O2 Alpha	0.36323 +/- 0.18056'	0.28159 +/- 0.20222'	0.9047619048
C4-T4 Alpha	0.42842 +/- 0.26768'	0.39223 +/- 0.25501'	1
C4-Fp1 Alpha	0.38936 +/- 0.1364'	0.26002 +/- 0.23575'	0.5555555556
C4-C3 Alpha	0.40439 +/- 0.15915'	0.29576 +/- 0.24432'	0.4126984127
C4-T3 Alpha	0.32015 +/- 0.152'	0.25324 +/- 0.24129'	0.9047619048
C4-O1 Alpha	0.3411 +/- 0.15067'	0.24384 +/- 0.25411'	0.7301587302
O2-T4 Alpha	0.4869 +/- 0.2249'	0.43358 +/- 0.29428'	0.9047619048
O2-Fp1 Alpha	0.41211 +/- 0.13224'	0.28589 +/- 0.21357'	0.7301587302
O2-C3 Alpha	0.40199 +/- 0.15325'	0.28256 +/- 0.24391'	0.7301587302
O2-T3 Alpha	0.35822 +/- 0.15412'	0.26456 +/- 0.2127'	0.7301587302
O2-O1 Alpha	0.44852 +/- 0.13407'	0.3124 +/- 0.23773'	0.9047619048
T4-Fp1 Alpha	0.36959 +/- 0.13917'	0.26505 +/- 0.24066'	1
T4-C3 Alpha	0.34685 +/- 0.10509'	0.2505 +/- 0.25472'	0.2857142857
T4-T3 Alpha	0.33789 +/- 0.13989'	0.25983 +/- 0.25129'	0.9047619048
T4-O1 Alpha	0.34458 +/- 0.13552'	0.24715 +/- 0.23395'	0.9047619048
Fp1-C3 Alpha	0.52185 +/- 0.14435'	0.36035 +/- 0.24447'	0.7301587302
Fp1-T3 Alpha	0.44769 +/- 0.20491'	0.30951 +/- 0.22575'	0.1904761905
Fp1-O1 Alpha	0.55033 +/- 0.19656'	0.34291 +/- 0.23205'	0.9047619048
C3-T3 Alpha	0.39806 +/- 0.27138'	0.44316 +/- 0.20254'	0.5555555556
C3-O1 Alpha	0.46125 +/- 0.14901'	0.33634 +/- 0.25694'	0.7301587302
T3-O1 Alpha	0.54853 +/- 0.21676'	0.42092 +/- 0.24519'	0.9047619048
Fp2-C4 Beta	0.39884 +/- 0.16502'	0.3003 +/- 0.15513'	0.9047619048
Fp2-O2 Beta	0.41875 +/- 0.15661'	0.3244 +/- 0.17342'	0.9047619048
Fp2-T4 Beta	0.31545 +/- 0.10425'	0.27461 +/- 0.18194'	0.1904761905
Fp2-Fp1 Beta	0.41227 +/- 0.15275'	0.34777 +/- 0.23594'	0.9047619048
Fp2-C3 Beta	0.37481 +/- 0.15004'	0.25122 +/- 0.1555'	0.9047619048
Fp2-T3 Beta	0.34644 +/- 0.15193'	0.2361 +/- 0.16127'	1
Fp2-O1 Beta	0.37369 +/- 0.15999'	0.23949 +/- 0.16607'	1
C4-O2 Beta	0.35361 +/- 0.15575'	0.31047 +/- 0.19734'	1
C4-T4 Beta	0.4194 +/- 0.25351'	0.42289 +/- 0.23603'	0.9047619048
C4-Fp1 Beta	0.36532 +/- 0.1396'	0.24778 +/- 0.2208'	0.7301587302

C4-C3 Beta	0.39957 +/- 0.15548'	0.28995 +/- 0.22747'	0.5555555556
C4-T3 Beta	0.29162 +/- 0.11246'	0.23729 +/- 0.22478'	0.7301587302
C4-O1 Beta	0.32332 +/- 0.11309'	0.23973 +/- 0.23961'	1
O2-T4 Beta	0.46368 +/- 0.21275'	0.39607 +/- 0.29202'	0.9047619048
O2-Fp1 Beta	0.4436 +/- 0.13561'	0.31543 +/- 0.21805'	1
O2-C3 Beta	0.41894 +/- 0.14467'	0.30244 +/- 0.22779'	0.7301587302
O2-T3 Beta	0.36537 +/- 0.11986'	0.26022 +/- 0.1949'	0.7301587302
O2-O1 Beta	0.48129 +/- 0.16853'	0.32854 +/- 0.24132'	0.9047619048
T4-Fp1 Beta	0.38297 +/- 0.17006'	0.27069 +/- 0.23652'	0.4126984127
T4-C3 Beta	0.33485 +/- 0.11546'	0.24353 +/- 0.23416'	0.4126984127
T4-T3 Beta	0.34829 +/- 0.14201'	0.25883 +/- 0.23453'	0.5555555556
T4-O1 Beta	0.3633 +/- 0.16246'	0.24362 +/- 0.21351'	0.4126984127
Fp1-C3 Beta	0.53148 +/- 0.19572'	0.30873 +/- 0.23812'	0.2857142857
Fp1-T3 Beta	0.4125 +/- 0.18844'	0.26898 +/- 0.20735'	0.1904761905
Fp1-O1 Beta	0.60532 +/- 0.18987'	0.29022 +/- 0.22298'	1
C3-T3 Beta	0.36811 +/- 0.25124'	0.42704 +/- 0.23481'	0.1111111111
C3-O1 Beta	0.49044 +/- 0.19706'	0.33661 +/- 0.27029'	0.9047619048
T3-O1 Beta	0.48776 +/- 0.20844'	0.44111 +/- 0.27799'	0.9047619048
Fp2-C4 Delta	0.38658 +/- 0.20659'	0.25384 +/- 0.10911'	0.4126984127
Fp2-O2 Delta	0.35762 +/- 0.11589'	0.22134 +/- 0.063843'	0.7301587302
Fp2-T4 Delta	0.3575 +/- 0.1671'	0.20871 +/- 0.081154'	0.5555555556
Fp2-Fp1 Delta	0.37929 +/- 0.15468'	0.33567 +/- 0.21601'	0.5555555556
Fp2-C3 Delta	0.32077 +/- 0.098154'	0.19466 +/- 0.069298'	0.4126984127
Fp2-T3 Delta	0.3494 +/- 0.11964'	0.19499 +/- 0.068755'	0.1904761905
Fp2-O1 Delta	0.32954 +/- 0.10037'	0.21736 +/- 0.070945'	0.4126984127
C4-O2 Delta	0.31018 +/- 0.18546'	0.27375 +/- 0.198'	0.9047619048
C4-T4 Delta	0.49648 +/- 0.26963'	0.38064 +/- 0.27231'	0.1904761905
C4-Fp1 Delta	0.32161 +/- 0.14282'	0.25119 +/- 0.18678'	0.5555555556
C4-C3 Delta	0.3273 +/- 0.10663'	0.27845 +/- 0.24598'	0.4126984127
C4-T3 Delta	0.27787 +/- 0.097859'	0.25597 +/- 0.24111'	0.4126984127
C4-O1 Delta	0.27945 +/- 0.090114'	0.24757 +/- 0.24862'	0.5555555556
O2-T4 Delta	0.49744 +/- 0.27169'	0.49469 +/- 0.26824'	1
O2-Fp1 Delta	0.29066 +/- 0.13282'	0.25313 +/- 0.14974'	1
O2-C3 Delta	0.29857 +/- 0.11478'	0.25584 +/- 0.23974'	0.7301587302
O2-T3 Delta	0.26809 +/- 0.10749'	0.26421 +/- 0.21681'	0.5555555556
O2-O1 Delta	0.31934 +/- 0.12508'	0.30735 +/- 0.21853'	0.7301587302
T4-Fp1 Delta	0.29179 +/- 0.11469'	0.23313 +/- 0.16714'	0.4126984127
T4-C3 Delta	0.28371 +/- 0.10929'	0.24692 +/- 0.24394'	0.5555555556
T4-T3 Delta	0.29063 +/- 0.091744'	0.2704 +/- 0.23163'	0.9047619048
T4-O1 Delta	0.27892 +/- 0.11494'	0.24945 +/- 0.2363'	0.4126984127
Fp1-C3 Delta	0.4286 +/- 0.19024'	0.39746 +/- 0.20359'	0.5555555556
Fp1-T3 Delta	0.35673 +/- 0.14212'	0.28757 +/- 0.16172'	0.2857142857
Fp1-O1 Delta	0.40079 +/- 0.12875'	0.33271 +/- 0.17065'	1

C3-T3 Delta	0.3742 +/- 0.25903'	0.39404 +/- 0.24395'	0.2857142857
C3-O1 Delta	0.35831 +/- 0.13268'	0.38998 +/- 0.26474'	0.9047619048
T3-O1 Delta	0.48392 +/- 0.1947'	0.39759 +/- 0.23761'	0.7301587302
Fp2-C4 Gamma	0.44381 +/- 0.21927'	0.29701 +/- 0.14952'	0.2857142857
Fp2-O2 Gamma	0.42621 +/- 0.18964'	0.30599 +/- 0.18219'	0.9047619048
Fp2-T4 Gamma	0.3143 +/- 0.13286'	0.26523 +/- 0.18757'	0.7301587302
Fp2-Fp1 Gamma	0.38161 +/- 0.18441'	0.31348 +/- 0.23787'	0.7301587302
Fp2-C3 Gamma	0.393 +/- 0.17849'	0.24117 +/- 0.16529'	1
Fp2-T3 Gamma	0.34346 +/- 0.20277'	0.20881 +/- 0.14586'	0.9047619048
Fp2-O1 Gamma	0.3382 +/- 0.18652'	0.23032 +/- 0.14994'	1
C4-O2 Gamma	0.35171 +/- 0.1409'	0.32693 +/- 0.20578'	0.9047619048
C4-T4 Gamma	0.40289 +/- 0.25446'	0.47014 +/- 0.25557'	0.7301587302
C4-Fp1 Gamma	0.31622 +/- 0.15506'	0.25291 +/- 0.20727'	0.5555555556
C4-C3 Gamma	0.36325 +/- 0.13675'	0.29807 +/- 0.22248'	0.9047619048
C4-T3 Gamma	0.28422 +/- 0.11551'	0.23451 +/- 0.20803'	0.7301587302
C4-O1 Gamma	0.28338 +/- 0.12576'	0.27034 +/- 0.22834'	0.5555555556
O2-T4 Gamma	0.46823 +/- 0.21849'	0.37653 +/- 0.29106'	0.9047619048
O2-Fp1 Gamma	0.39842 +/- 0.13399'	0.29704 +/- 0.21436'	0.7301587302
O2-C3 Gamma	0.42637 +/- 0.14948'	0.2937 +/- 0.22059'	1
O2-T3 Gamma	0.38408 +/- 0.17961'	0.24197 +/- 0.18273'	1
O2-O1 Gamma	0.45333 +/- 0.19057'	0.3526 +/- 0.22675'	0.2857142857
T4-Fp1 Gamma	0.34652 +/- 0.1068'	0.26349 +/- 0.22198'	0.4126984127
T4-C3 Gamma	0.33107 +/- 0.085006'	0.24723 +/- 0.22796'	0.4126984127
T4-T3 Gamma	0.37 +/- 0.13339'	0.25569 +/- 0.2226'	0.4126984127
T4-O1 Gamma	0.3347 +/- 0.11965'	0.2546 +/- 0.19428'	0.5555555556
Fp1-C3 Gamma	0.50211 +/- 0.20083'	0.27814 +/- 0.19572'	0.1111111111
Fp1-T3 Gamma	0.37815 +/- 0.20485'	0.23976 +/- 0.18735'	0.2857142857
Fp1-O1 Gamma	0.47778 +/- 0.16781'	0.2709 +/- 0.19947'	1
C3-T3 Gamma	0.44613 +/- 0.27008'	0.45628 +/- 0.26072'	0.06349206349
C3-O1 Gamma	0.44283 +/- 0.16621'	0.37106 +/- 0.23455'	1
T3-O1 Gamma	0.46218 +/- 0.20809'	0.40525 +/- 0.18309'	0.7301587302
Fp2-C4 Overall	0.40873 +/- 0.1817'	0.32037 +/- 0.13853'	0.2857142857
Fp2-O2 Overall	0.40186 +/- 0.11018'	0.3057 +/- 0.14717'	0.9047619048
Fp2-T4 Overall	0.31385 +/- 0.095162'	0.28277 +/- 0.17061'	0.5555555556
Fp2-Fp1 Overall	0.41377 +/- 0.10126'	0.34569 +/- 0.19498'	0.5555555556
Fp2-C3 Overall	0.37304 +/- 0.11035'	0.26211 +/- 0.15499'	0.5555555556
Fp2-T3 Overall	0.36194 +/- 0.14128'	0.24555 +/- 0.12504'	1
Fp2-O1 Overall	0.35476 +/- 0.11247'	0.25406 +/- 0.12752'	1
C4-O2 Overall	0.34112 +/- 0.1397'	0.33461 +/- 0.1799'	0.9047619048
C4-T4 Overall	0.43081 +/- 0.25955'	0.44938 +/- 0.20759'	0.5555555556
C4-Fp1 Overall	0.33701 +/- 0.11294'	0.28808 +/- 0.19071'	0.9047619048
C4-C3 Overall	0.37598 +/- 0.13841'	0.33706 +/- 0.22075'	0.9047619048
C4-T3 Overall	0.30556 +/- 0.10446'	0.28804 +/- 0.20593'	1

C4-O1 Overall	0.29068 +/- 0.10042'	0.30301 +/- 0.21602'	1
O2-T4 Overall	0.47982 +/- 0.20184'	0.44992 +/- 0.25961'	0.7301587302
O2-Fp1 Overall	0.40458 +/- 0.11861'	0.3215 +/- 0.20927'	0.5555555556
O2-C3 Overall	0.40377 +/- 0.12793'	0.32003 +/- 0.22882'	0.4126984127
O2-T3 Overall	0.37888 +/- 0.1255'	0.28861 +/- 0.18524'	0.5555555556
O2-O1 Overall	0.46893 +/- 0.16667'	0.37464 +/- 0.22163'	0.9047619048
T4-Fp1 Overall	0.3335 +/- 0.11186'	0.29601 +/- 0.22197'	0.1904761905
T4-C3 Overall	0.33531 +/- 0.10693'	0.27987 +/- 0.25224'	0.1904761905
T4-T3 Overall	0.35537 +/- 0.12808'	0.29054 +/- 0.22619'	0.7301587302
T4-O1 Overall	0.34664 +/- 0.095155'	0.28004 +/- 0.19697'	0.9047619048
Fp1-C3 Overall	0.49978 +/- 0.18802'	0.36781 +/- 0.18441'	0.1904761905
Fp1-T3 Overall	0.38379 +/- 0.13499'	0.2958 +/- 0.18691'	0.2857142857
Fp1-O1 Overall	0.47094 +/- 0.13709'	0.33653 +/- 0.19107'	0.7301587302
C3-T3 Overall	0.45252 +/- 0.2586'	0.49174 +/- 0.19806'	0.06349206349
C3-O1 Overall	0.43662 +/- 0.15782'	0.40603 +/- 0.20104'	0.9047619048
T3-O1 Overall	0.48316 +/- 0.17354'	0.44955 +/- 0.15809'	0.7301587302

Table S4. Overall power and power within each band at each electrode location, treated vs. untreated at 12 months.

Band	Treated	Untreated	*p*
Fp2 Theta	4.346 +/- 11.2209'	-4.5294 +/- 4.7287'	0.2224187577
Fp2 Alpha	3.5206 +/- 8.4127'	-3.0898 +/- 3.2086'	0.2224187577
Fp2 Beta	2.8459 +/- 5.7358'	-2.3148 +/- 2.8251'	0.03146853147
Fp2 Delta	5.0624 +/- 13.4924'	-6.321 +/- 6.182'	0.09391197038
Fp2 Gamma	1.1447 +/- 0.73124'	0.97169 +/- 0.34955'	0.1902509255
Fp2 Overall	-1.5346 +/- 4.561'	-4.604 +/- 1.9025'	0.2224187577
C4 Theta	3.7585 +/- 11.2755'	-12.6099 +/- 14.725'	0.03146853147
C4 Alpha	3.1006 +/- 8.5459'	-9.3185 +/- 11.1534'	0.03146853147
C4 Beta	2.0937 +/- 5.7793'	-8.5623 +/- 10.4676'	0.02443438914
C4 Delta	4.2109 +/- 13.4868'	-17.2557 +/- 20.5873'	0.03998354587
C4 Gamma	0.82813 +/- 0.93321'	-0.42995 +/- 2.0711'	0.11349239
C4 Overall	0.27311 +/- 5.4459'	-3.9997 +/- 3.5107'	0.09391197038
T4 Theta	0.8291 +/- 8.1605'	109.694 +/- 315.0896'	0.7304401481
T4 Alpha	0.74567 +/- 6.4396'	94.4001 +/- 273.0846'	0.7304401481
T4 Beta	0.67549 +/- 5.1402'	93.7959 +/- 270.4052'	0.7961744138
T4 Delta	0.76267 +/- 9.902'	114.8333 +/- 324.1421'	0.7304401481
T4 Gamma	0.9266 +/- 0.52462'	18.8093 +/- 51.5697'	0.2973262032
T4 Overall	0.20284 +/- 5.7367'	-2.0923 +/- 3.254'	0.2580830934
O2 Theta	1.2135 +/- 19.7864'	-41.3183 +/- 107.6733'	0.09391197038
O2 Alpha	0.96673 +/- 15.307'	-29.2041 +/- 75.0885'	0.11349239
O2 Beta	0.67809 +/- 11.4798'	-25.2616 +/- 66.1963'	0.09391197038
O2 Delta	1.3924 +/- 21.7912'	-51.6634 +/- 135.8023'	0.11349239
O2 Gamma	1.0434 +/- 0.24661'	-0.40508 +/- 3.6966'	0.9314273961

O2 Overall	-0.93669 +/- 5.6042'	-4.2626 +/- 3.1'	0.1359111477
Fp1 Theta	-5.6436 +/- 14.5087'	-2.8628 +/- 1.7287'	0.2973262032
Fp1 Alpha	-4.0319 +/- 10.8357'	-1.8982 +/- 1.2077'	0.1614973262
Fp1 Beta	-2.2663 +/- 7.8564'	-1.2907 +/- 1.0829'	0.2973262032
Fp1 Delta	-6.6069 +/- 17.7835'	-3.9248 +/- 2.417'	0.2580830934
Fp1 Gamma	1.3564 +/- 0.39207'	1.0446 +/- 0.16285'	0.09391197038
Fp1 Overall	-2.0483 +/- 2.7908'	-5.8652 +/- 2.1439'	0.007774578363
C3 Theta	-1.5415 +/- 15.1929'	5.1215 +/- 21.1862'	0.6664747018
C3 Alpha	-0.93597 +/- 11.5032'	3.8199 +/- 15.3817'	0.7304401481
C3 Beta	-0.15355 +/- 8.3969'	4.0642 +/- 15.2576'	0.6664747018
C3 Delta	-1.6323 +/- 17.5461'	4.5737 +/- 22.6244'	0.6664747018
C3 Gamma	1.6898 +/- 0.6791'	2.5605 +/- 4.9571'	0.06252570958
C3 Overall	0.34711 +/- 4.9042'	-4.0909 +/- 4.3529'	0.09391197038
T3 Theta	4.6367 +/- 12.0363'	3.8169 +/- 10.7411'	0.6664747018
T3 Alpha	3.7768 +/- 9.1438'	3.0475 +/- 8.3598'	0.7304401481
T3 Beta	3.6211 +/- 7.2616'	3.4279 +/- 9.124'	0.5457013575
T3 Delta	5.3024 +/- 14.2394'	5.6136 +/- 15.966'	0.6048128342
T3 Gamma	1.6364 +/- 0.46674'	1.6016 +/- 1.3299'	0.2973262032
T3 Overall	1.909 +/- 6.9675'	-1.6894 +/- 4.1151'	0.38650761
O1 Theta	0.65271 +/- 28.8086'	-3.0936 +/- 6.9851'	0.4362813657
O1 Alpha	0.64892 +/- 21.4488'	-2.0775 +/- 5.319'	0.5457013575
O1 Beta	0.97378 +/- 16.1414'	-1.3274 +/- 4.3119'	0.4362813657
O1 Delta	0.21728 +/- 35.0435'	-4.4437 +/- 8.4833'	0.4894282188
O1 Gamma	1.6256 +/- 1.0923'	0.92084 +/- 0.41037'	0.03998354587
O1 Overall	0.044024 +/- 6.3288'	-4.0077 +/- 3.1238'	0.1614973262
R hemi Theta	2.5368 +/- 7.5549'	12.8093 +/- 85.5026'	0.05030851501
R hemi Alpha	2.0833 +/- 5.6825'	13.1967 +/- 72.1623'	0.05030851501
R hemi Beta	1.5733 +/- 4.2176'	14.4142 +/- 70.5478'	0.06252570958
R hemi Delta	2.8571 +/- 8.8378'	9.8981 +/- 91.0119'	0.05030851501
R hemi Gamma	0.98572 +/- 0.47304'	4.7365 +/- 12.6305'	0.4894282188
R hemi Overall	-0.4988 +/- 4.7612'	-3.7396 +/- 2.5473'	0.09391197038
L hemi Theta	-0.4739 +/- 8.4564'	0.74554 +/- 7.1952'	0.863307281
L hemi Alpha	-0.13556 +/- 6.4339'	0.72299 +/- 5.3994'	0.9314273961
L hemi Beta	0.54377 +/- 4.7383'	1.2185 +/- 5.1383'	0.7304401481
L hemi Delta	-0.67983 +/- 10.201'	0.4547 +/- 7.9624'	0.7304401481
L hemi Gamma	1.5771 +/- 0.35924'	1.5319 +/- 1.2919'	0.07700534759
L hemi Overall	0.062953 +/- 5.0083'	-3.9133 +/- 2.9704'	0.09391197038
Overall Theta	1.0314 +/- 5.9443'	6.7769 +/- 46.0782'	0.3401069519
Overall Alpha	0.97391 +/- 4.5747'	6.9598 +/- 38.5928'	0.38650761
Overall Beta	1.0585 +/- 3.4403'	7.8161 +/- 37.6269'	0.2973262032
Overall Delta	1.0886 +/- 6.8119'	5.1762 +/- 49.0286'	0.2580830934
Overall Gamma	1.2814 +/- 0.28797'	3.1342 +/- 6.9404'	0.07700534759
Overall Overall	-0.21794 +/- 4.709'	-3.8265 +/- 2.7047'	0.06252570958

Table S5. Hemispheric asymmetry, overall and within each band, untreated vs. treated at 12 months.

Band	Treated	Untreated	*p*
Frontal Theta	-9.9898 +/- 21.7163'	1.6665 +/- 3.4764'	0.07700534759
Frontal Alpha	-7.5524 +/- 16.1438'	1.1917 +/- 2.4171'	0.07700534759
Frontal Beta	-5.1122 +/- 11.3833'	1.0241 +/- 2.2472'	0.07700534759
Frontal Delta	-11.6691 +/- 26.2558'	2.3962 +/- 4.4571'	0.07700534759
Frontal Gamma	0.2117 +/- 0.73106'	0.07288 +/- 0.32701'	0.9314273961
Frontal Overall	-0.51373 +/- 2.5535'	-1.2613 +/- 1.3139'	0.4362813657
Parietal Theta	-5.3001 +/- 19.11'	17.7314 +/- 24.4903'	0.1614973262
Parietal Alpha	-4.0366 +/- 14.3672'	13.1384 +/- 18.2628'	0.1614973262
Parietal Beta	-2.2473 +/- 10.2565'	12.6266 +/- 17.7858'	0.1614973262
Parietal Delta	-5.8433 +/- 22.5219'	21.8295 +/- 27.9365'	0.1902509255
Parietal Gamma	0.8617 +/- 1.2591'	2.9905 +/- 5.6949'	0.7961744138
Parietal Overall	0.073989 +/- 3.5537'	-0.091209 +/- 2.4644'	0.9314273961
Temporal Theta	3.8076 +/- 14.8732'	-105.8772 +/- 314.0665'	0.38650761
Temporal Alpha	3.0311 +/- 11.436'	-91.3527 +/- 272.1884'	0.4894282188
Temporal Beta	2.9456 +/- 9.1562'	-90.3684 +/- 269.7723'	0.5457013575
Temporal Delta	4.5398 +/- 18.0382'	-109.2194 +/- 323.4772'	0.4894282188
Temporal Gamma	0.70986 +/- 0.88492'	-17.2078 +/- 51.544'	0.01419169066
Temporal Overall	1.7062 +/- 5.4437'	0.40283 +/- 1.8606'	0.6048128342
Occipital Theta	-0.56087 +/- 27.8406'	38.2249 +/- 103.0521'	0.06252570958
Occipital Alpha	-0.3178 +/- 20.7374'	27.1274 +/- 71.6988'	0.07700534759
Occipital Beta	0.29551 +/- 15.665'	23.9342 +/- 63.2968'	0.07700534759
Occipital Delta	-1.175 +/- 33.3575'	47.2197 +/- 130.0555'	0.09391197038
Occipital Gamma	0.58221 +/- 1.1827'	1.3258 +/- 3.6129'	0.2224187577
Occipital Overall	0.9807 +/- 3.5504'	0.2549 +/- 1.8129'	0.5457013575
Overall Theta	-12.0427 +/- 43.05'	-48.2534 +/- 315.753'	0.5457013575
Overall Alpha	-8.8759 +/- 31.9156'	-49.8963 +/- 268.7922'	0.5457013575
Overall Beta	-4.1182 +/- 23.0267'	-52.7828 +/- 263.6175'	0.6048128342
Overall Delta	-14.1473 +/- 53.4749'	-37.7745 +/- 336.5284'	0.4894282188
Overall Gamma	2.3654 +/- 2.446'	-12.8188 +/- 45.5572'	0.1359111477
Overall Overall	2.2472 +/- 10.4338'	-0.69472 +/- 4.6668'	0.7961744138

Table S6. Inter-electrode coherence measures in the overall spectrum, untreated vs. treated at 12 months.

Band	Treated	Untreated	*p*
Fp2-C4 Theta	0.4877 +/- 0.30732'	0.36682 +/- 0.24738'	0.38650761
Fp2-O2 Theta	0.47656 +/- 0.27582'	0.3651 +/- 0.24987'	0.3401069519
Fp2-T4 Theta	0.3127 +/- 0.21923'	0.29357 +/- 0.23141'	0.4362813657
Fp2-Fp1 Theta	0.40403 +/- 0.26857'	0.34769 +/- 0.26482'	0.7304401481
Fp2-C3 Theta	0.26735 +/- 0.10601'	0.28589 +/- 0.1489'	1
Fp2-T3 Theta	0.25104 +/- 0.10249'	0.29253 +/- 0.17014'	0.7961744138

Fp2-O1 Theta	0.31389 +/- 0.17544'	0.2656 +/- 0.13721'	0.6048128342
C4-O2 Theta	0.40502 +/- 0.23058'	0.36346 +/- 0.236'	0.5457013575
C4-T4 Theta	0.46569 +/- 0.32496'	0.32334 +/- 0.21726'	0.2973262032
C4-Fp1 Theta	0.31488 +/- 0.057842'	0.26451 +/- 0.13775'	0.05030851501
C4-C3 Theta	0.26776 +/- 0.1138'	0.33329 +/- 0.13861'	0.6048128342
C4-T3 Theta	0.23185 +/- 0.1016'	0.29444 +/- 0.17917'	0.863307281
C4-O1 Theta	0.24027 +/- 0.088439'	0.22884 +/- 0.10157'	0.3401069519
O2-T4 Theta	0.41396 +/- 0.20807'	0.44796 +/- 0.23942'	0.7961744138
O2-Fp1 Theta	0.37292 +/- 0.17404'	0.26743 +/- 0.11093'	0.1902509255
O2-C3 Theta	0.27888 +/- 0.10961'	0.29048 +/- 0.17897'	0.6664747018
O2-T3 Theta	0.31042 +/- 0.10167'	0.28452 +/- 0.17145'	0.4362813657
O2-O1 Theta	0.3835 +/- 0.20224'	0.36102 +/- 0.2122'	0.863307281
T4-Fp1 Theta	0.25123 +/- 0.084639'	0.26789 +/- 0.1569'	0.863307281
T4-C3 Theta	0.23466 +/- 0.12234'	0.27096 +/- 0.18388'	0.863307281
T4-T3 Theta	0.26663 +/- 0.1249'	0.3258 +/- 0.16093'	0.6048128342
T4-O1 Theta	0.28291 +/- 0.14088'	0.26737 +/- 0.16943'	0.4362813657
Fp1-C3 Theta	0.37484 +/- 0.15863'	0.39525 +/- 0.21459'	1
Fp1-T3 Theta	0.32069 +/- 0.10722'	0.3134 +/- 0.19248'	0.4362813657
Fp1-O1 Theta	0.40555 +/- 0.25097'	0.33325 +/- 0.13769'	0.7304401481
C3-T3 Theta	0.3911 +/- 0.19017'	0.365 +/- 0.24501'	0.3401069519
C3-O1 Theta	0.28291 +/- 0.11877'	0.33251 +/- 0.18279'	0.863307281
T3-O1 Theta	0.50558 +/- 0.24393'	0.41087 +/- 0.21321'	0.4894282188
Fp2-C4 Alpha	0.45278 +/- 0.31255'	0.38901 +/- 0.19245'	1
Fp2-O2 Alpha	0.45398 +/- 0.25289'	0.3991 +/- 0.27182'	0.6048128342
Fp2-T4 Alpha	0.29749 +/- 0.17816'	0.33401 +/- 0.26466'	0.7304401481
Fp2-Fp1 Alpha	0.3756 +/- 0.23692'	0.3616 +/- 0.27758'	1
Fp2-C3 Alpha	0.23675 +/- 0.11282'	0.32741 +/- 0.26468'	1
Fp2-T3 Alpha	0.25453 +/- 0.11872'	0.31572 +/- 0.25222'	1
Fp2-O1 Alpha	0.30513 +/- 0.13063'	0.29144 +/- 0.19382'	0.6664747018
C4-O2 Alpha	0.40335 +/- 0.22984'	0.35822 +/- 0.21868'	0.5457013575
C4-T4 Alpha	0.4317 +/- 0.33232'	0.35371 +/- 0.21468'	1
C4-Fp1 Alpha	0.28109 +/- 0.10645'	0.31927 +/- 0.21564'	1
C4-C3 Alpha	0.25158 +/- 0.12137'	0.34969 +/- 0.17888'	0.3401069519
C4-T3 Alpha	0.22474 +/- 0.12916'	0.29939 +/- 0.20752'	0.863307281
C4-O1 Alpha	0.2491 +/- 0.12196'	0.28717 +/- 0.2005'	0.9314273961
O2-T4 Alpha	0.3966 +/- 0.22393'	0.47503 +/- 0.28516'	0.9314273961
O2-Fp1 Alpha	0.33405 +/- 0.11998'	0.30109 +/- 0.19959'	0.2580830934
O2-C3 Alpha	0.25965 +/- 0.097567'	0.3076 +/- 0.24042'	0.6664747018
O2-T3 Alpha	0.29004 +/- 0.10088'	0.29354 +/- 0.23201'	0.38650761
O2-O1 Alpha	0.38776 +/- 0.16487'	0.38545 +/- 0.25847'	0.7304401481
T4-Fp1 Alpha	0.24944 +/- 0.11065'	0.30578 +/- 0.23906'	1
T4-C3 Alpha	0.22425 +/- 0.13115'	0.29726 +/- 0.24995'	0.9314273961
T4-T3 Alpha	0.26807 +/- 0.15089'	0.33271 +/- 0.21011'	0.7961744138

T4-O1 Alpha	0.31002 +/- 0.18669'	0.29087 +/- 0.23666'	0.863307281
Fp1-C3 Alpha	0.40276 +/- 0.14175'	0.39945 +/- 0.22008'	0.6664747018
Fp1-T3 Alpha	0.33438 +/- 0.13156'	0.33129 +/- 0.22615'	0.3401069519
Fp1-O1 Alpha	0.36868 +/- 0.22699'	0.39871 +/- 0.1834'	0.7961744138
C3-T3 Alpha	0.42965 +/- 0.2014'	0.40546 +/- 0.29913'	0.38650761
C3-O1 Alpha	0.31452 +/- 0.16321'	0.35477 +/- 0.23514'	1
T3-O1 Alpha	0.53567 +/- 0.27481'	0.42569 +/- 0.25578'	0.5457013575
Fp2-C4 Beta	0.41586 +/- 0.27326'	0.3984 +/- 0.17589'	0.6664747018
Fp2-O2 Beta	0.4244 +/- 0.22727'	0.3848 +/- 0.238'	0.4362813657
Fp2-T4 Beta	0.27303 +/- 0.15414'	0.32683 +/- 0.2301'	0.863307281
Fp2-Fp1 Beta	0.362 +/- 0.15392'	0.32204 +/- 0.22687'	0.6048128342
Fp2-C3 Beta	0.26387 +/- 0.12876'	0.31446 +/- 0.24829'	1
Fp2-T3 Beta	0.29067 +/- 0.14085'	0.27122 +/- 0.20212'	0.6048128342
Fp2-O1 Beta	0.31111 +/- 0.14105'	0.27238 +/- 0.18265'	0.38650761
C4-O2 Beta	0.39189 +/- 0.21512'	0.36309 +/- 0.17577'	0.7961744138
C4-T4 Beta	0.44795 +/- 0.30815'	0.41282 +/- 0.23412'	1
C4-Fp1 Beta	0.28816 +/- 0.096975'	0.31525 +/- 0.20217'	1
C4-C3 Beta	0.25297 +/- 0.13445'	0.32063 +/- 0.16803'	0.6664747018
C4-T3 Beta	0.237 +/- 0.1267'	0.24947 +/- 0.14502'	0.863307281
C4-O1 Beta	0.27018 +/- 0.12294'	0.29541 +/- 0.19087'	0.9314273961
O2-T4 Beta	0.40622 +/- 0.21194'	0.42046 +/- 0.28987'	0.9314273961
O2-Fp1 Beta	0.29991 +/- 0.12251'	0.27607 +/- 0.15506'	0.5457013575
O2-C3 Beta	0.29353 +/- 0.12566'	0.29178 +/- 0.20122'	0.4362813657
O2-T3 Beta	0.32615 +/- 0.15418'	0.26518 +/- 0.18819'	0.2580830934
O2-O1 Beta	0.39907 +/- 0.13943'	0.37253 +/- 0.22341'	0.5457013575
T4-Fp1 Beta	0.25048 +/- 0.11366'	0.27274 +/- 0.20269'	0.6048128342
T4-C3 Beta	0.24062 +/- 0.15353'	0.25719 +/- 0.21168'	0.9314273961
T4-T3 Beta	0.2704 +/- 0.16635'	0.29604 +/- 0.18457'	0.9314273961
T4-O1 Beta	0.29772 +/- 0.19626'	0.26359 +/- 0.19106'	0.6664747018
Fp1-C3 Beta	0.41887 +/- 0.14621'	0.33896 +/- 0.17125'	0.2580830934
Fp1-T3 Beta	0.37072 +/- 0.17346'	0.27811 +/- 0.14343'	0.11349239
Fp1-O1 Beta	0.39611 +/- 0.21986'	0.3348 +/- 0.13533'	0.4894282188
C3-T3 Beta	0.49482 +/- 0.23227'	0.43683 +/- 0.23529'	0.7304401481
C3-O1 Beta	0.34199 +/- 0.16235'	0.32869 +/- 0.21663'	0.5457013575
T3-O1 Beta	0.52323 +/- 0.29956'	0.37922 +/- 0.22598'	0.3401069519
Fp2-C4 Delta	0.48156 +/- 0.26819'	0.35407 +/- 0.2463'	0.2973262032
Fp2-O2 Delta	0.46589 +/- 0.24109'	0.36229 +/- 0.25946'	0.3401069519
Fp2-T4 Delta	0.31687 +/- 0.25126'	0.2607 +/- 0.22575'	0.2973262032
Fp2-Fp1 Delta	0.39657 +/- 0.24208'	0.35301 +/- 0.23469'	0.6048128342
Fp2-C3 Delta	0.24948 +/- 0.092452'	0.275 +/- 0.099888'	0.5457013575
Fp2-T3 Delta	0.24738 +/- 0.10231'	0.23817 +/- 0.10332'	0.7304401481
Fp2-O1 Delta	0.31842 +/- 0.17022'	0.25555 +/- 0.10617'	0.6221719457
C4-O2 Delta	0.40914 +/- 0.15797'	0.36913 +/- 0.23217'	0.3401069519

C4-T4 Delta	0.48818 +/- 0.34848'	0.35356 +/- 0.19108'	0.5457013575
C4-Fp1 Delta	0.31715 +/- 0.082133'	0.29053 +/- 0.10659'	0.6664747018
C4-C3 Delta	0.25443 +/- 0.10737'	0.34162 +/- 0.13116'	0.2224187577
C4-T3 Delta	0.24383 +/- 0.12012'	0.28874 +/- 0.15294'	0.9314273961
C4-O1 Delta	0.26491 +/- 0.13018'	0.23867 +/- 0.09093'	0.6664747018
O2-T4 Delta	0.40139 +/- 0.21178'	0.35067 +/- 0.24107'	0.4362813657
O2-Fp1 Delta	0.37567 +/- 0.1812'	0.26183 +/- 0.092159'	0.1614973262
O2-C3 Delta	0.25398 +/- 0.067362'	0.31377 +/- 0.13597'	0.3401069519
O2-T3 Delta	0.26585 +/- 0.092402'	0.25199 +/- 0.14335'	0.4362813657
O2-O1 Delta	0.33294 +/- 0.18492'	0.36149 +/- 0.16203'	0.7304401481
T4-Fp1 Delta	0.24025 +/- 0.090275'	0.25588 +/- 0.098832'	0.7304401481
T4-C3 Delta	0.23078 +/- 0.10605'	0.28282 +/- 0.13776'	0.6048128342
T4-T3 Delta	0.23033 +/- 0.12613'	0.28229 +/- 0.12105'	0.6664747018
T4-O1 Delta	0.29488 +/- 0.16025'	0.23857 +/- 0.1166'	0.38650761
Fp1-C3 Delta	0.35674 +/- 0.12247'	0.33413 +/- 0.12178'	0.7961744138
Fp1-T3 Delta	0.2805 +/- 0.090775'	0.25534 +/- 0.11661'	0.4894282188
Fp1-O1 Delta	0.40638 +/- 0.24933'	0.25521 +/- 0.0924'	0.2580830934
C3-T3 Delta	0.38972 +/- 0.20041'	0.35617 +/- 0.21558'	0.5457013575
C3-O1 Delta	0.28972 +/- 0.09552'	0.29129 +/- 0.15857'	0.5457013575
T3-O1 Delta	0.43364 +/- 0.22177'	0.35889 +/- 0.23647'	0.38650761
Fp2-C4 Gamma	0.4505 +/- 0.27769'	0.38127 +/- 0.20789'	0.6664747018
Fp2-O2 Gamma	0.39154 +/- 0.2013'	0.34242 +/- 0.19967'	0.4362813657
Fp2-T4 Gamma	0.30502 +/- 0.19941'	0.27039 +/- 0.16307'	0.7304401481
Fp2-Fp1 Gamma	0.38662 +/- 0.2254'	0.23171 +/- 0.15786'	0.1359111477
Fp2-C3 Gamma	0.3128 +/- 0.16806'	0.25352 +/- 0.1683'	0.3401069519
Fp2-T3 Gamma	0.31592 +/- 0.18116'	0.18563 +/- 0.084138'	0.11349239
Fp2-O1 Gamma	0.34019 +/- 0.20613'	0.21739 +/- 0.12311'	0.11349239
C4-O2 Gamma	0.39939 +/- 0.18859'	0.33671 +/- 0.14095'	0.5457013575
C4-T4 Gamma	0.47538 +/- 0.30121'	0.40544 +/- 0.20904'	0.5457013575
C4-Fp1 Gamma	0.31188 +/- 0.11897'	0.24208 +/- 0.15621'	0.1902509255
C4-C3 Gamma	0.28802 +/- 0.15349'	0.29427 +/- 0.19081'	0.7961744138
C4-T3 Gamma	0.26743 +/- 0.13315'	0.19322 +/- 0.071853'	0.1614973262
C4-O1 Gamma	0.28411 +/- 0.11631'	0.25586 +/- 0.12924'	0.5457013575
O2-T4 Gamma	0.38681 +/- 0.17538'	0.35851 +/- 0.24281'	0.6048128342
O2-Fp1 Gamma	0.2989 +/- 0.11947'	0.2099 +/- 0.065715'	0.11349239
O2-C3 Gamma	0.27463 +/- 0.094455'	0.27039 +/- 0.17969'	0.6048128342
O2-T3 Gamma	0.28047 +/- 0.093754'	0.22625 +/- 0.12394'	0.2224187577
O2-O1 Gamma	0.38401 +/- 0.1255'	0.36763 +/- 0.19165'	0.7961744138
T4-Fp1 Gamma	0.26777 +/- 0.19241'	0.17655 +/- 0.051623'	0.2973262032
T4-C3 Gamma	0.25548 +/- 0.19067'	0.21022 +/- 0.14394'	0.6664747018
T4-T3 Gamma	0.26703 +/- 0.18753'	0.21486 +/- 0.10027'	0.6664747018
T4-O1 Gamma	0.3001 +/- 0.21919'	0.22623 +/- 0.10765'	0.6664747018
Fp1-C3 Gamma	0.46708 +/- 0.23113'	0.27847 +/- 0.14423'	0.06252570958

Fp1-T3 Gamma	0.44288 +/- 0.18082'	0.1983 +/- 0.031997'	0.0002879473468
Fp1-O1 Gamma	0.45199 +/- 0.23509'	0.22734 +/- 0.040902'	0.06252570958
C3-T3 Gamma	0.51391 +/- 0.26541'	0.5295 +/- 0.16844'	0.7961744138
C3-O1 Gamma	0.403 +/- 0.21339'	0.33676 +/- 0.20308'	0.6048128342
T3-O1 Gamma	0.5187 +/- 0.31328'	0.33567 +/- 0.1484'	0.2973262032
Fp2-C4 Overall	0.45691 +/- 0.25707'	0.37512 +/- 0.17018'	0.6048128342
Fp2-O2 Overall	0.40711 +/- 0.17137'	0.3595 +/- 0.16046'	0.5457013575
Fp2-T4 Overall	0.29806 +/- 0.15306'	0.28749 +/- 0.16097'	0.6664747018
Fp2-Fp1 Overall	0.39969 +/- 0.19445'	0.31501 +/- 0.2122'	0.38650761
Fp2-C3 Overall	0.29405 +/- 0.13555'	0.3057 +/- 0.20421'	0.7961744138
Fp2-T3 Overall	0.29902 +/- 0.14535'	0.26276 +/- 0.14748'	0.4894282188
Fp2-O1 Overall	0.34769 +/- 0.13918'	0.2834 +/- 0.14961'	0.2973262032
C4-O2 Overall	0.38407 +/- 0.16788'	0.37566 +/- 0.15939'	1
C4-T4 Overall	0.45379 +/- 0.27232'	0.41274 +/- 0.14204'	0.7961744138
C4-Fp1 Overall	0.31492 +/- 0.086371'	0.2757 +/- 0.16829'	0.2973262032
C4-C3 Overall	0.2887 +/- 0.13999'	0.32477 +/- 0.19284'	1
C4-T3 Overall	0.27426 +/- 0.13591'	0.25515 +/- 0.13333'	0.4362813657
C4-O1 Overall	0.29915 +/- 0.13311'	0.28093 +/- 0.13939'	0.7961744138
O2-T4 Overall	0.39028 +/- 0.14332'	0.39641 +/- 0.2121'	0.7961744138
O2-Fp1 Overall	0.30889 +/- 0.094482'	0.27378 +/- 0.099554'	0.5457013575
O2-C3 Overall	0.26681 +/- 0.099421'	0.30848 +/- 0.20679'	0.9314273961
O2-T3 Overall	0.27703 +/- 0.081127'	0.2839 +/- 0.159'	0.9314273961
O2-O1 Overall	0.39276 +/- 0.12981'	0.40015 +/- 0.1752'	1
T4-Fp1 Overall	0.25466 +/- 0.11214'	0.24623 +/- 0.11419'	0.6048128342
T4-C3 Overall	0.24504 +/- 0.15191'	0.25229 +/- 0.17248'	1
T4-T3 Overall	0.26191 +/- 0.15273'	0.26896 +/- 0.12821'	0.6664747018
T4-O1 Overall	0.29614 +/- 0.17026'	0.2784 +/- 0.14173'	0.7961744138
Fp1-C3 Overall	0.41227 +/- 0.12452'	0.33954 +/- 0.14978'	0.2973262032
Fp1-T3 Overall	0.37269 +/- 0.10512'	0.26092 +/- 0.096722'	0.02443438914
Fp1-O1 Overall	0.40335 +/- 0.17371'	0.30422 +/- 0.1042'	0.1902509255
C3-T3 Overall	0.44756 +/- 0.21416'	0.50115 +/- 0.12092'	0.38650761
C3-O1 Overall	0.35549 +/- 0.1491'	0.35397 +/- 0.20924'	0.5457013575
T3-O1 Overall	0.51082 +/- 0.29561'	0.41007 +/- 0.15415'	0.6048128342

Table S7. Overall power and power within each band at each electrode location, treated at baseline vs. at 12 months.

Band	Baseline	12 Months	*p*
Fp2 Theta	-47.5507 +/- 143.8417'	4.346 +/- 11.2209'	0.09765625
Fp2 Alpha	-41.4767 +/- 127.0681'	3.5206 +/- 8.4127'	0.09765625
Fp2 Beta	-19.384 +/- 62.4975'	2.8459 +/- 5.7358'	0.09765625
Fp2 Delta	-51.7872 +/- 158.3624'	5.0624 +/- 13.4924'	0.09765625
Fp2 Gamma	13.649 +/- 34.4742'	1.1447 +/- 0.73124'	0.01171875
Fp2 Overall	-1.9291 +/- 4.3741'	-1.5346 +/- 4.561'	1

C4 Theta	-5.2122 +/- 49.3782'	3.7585 +/- 11.2755'	0.359375
C4 Alpha	-5.6285 +/- 41.5282'	3.1006 +/- 8.5459'	0.42578125
C4 Beta	-3.1068 +/- 24.7323'	2.0937 +/- 5.7793'	0.5703125
C4 Delta	-3.8606 +/- 60.0226'	4.2109 +/- 13.4868'	0.359375
C4 Gamma	2.2582 +/- 10.6184'	0.82813 +/- 0.93321'	0.203125
C4 Overall	1.0284 +/- 5.1993'	0.27311 +/- 5.4459'	0.91015625
T4 Theta	-0.92801 +/- 9.2063'	0.8291 +/- 8.1605'	0.734375
T4 Alpha	-0.6639 +/- 7.2729'	0.74567 +/- 6.4396'	0.734375
T4 Beta	0.029378 +/- 4.6261'	0.67549 +/- 5.1402'	0.8203125
T4 Delta	-1.0907 +/- 11.2634'	0.76267 +/- 9.902'	0.734375
T4 Gamma	1.3624 +/- 1.6974'	0.9266 +/- 0.52462'	0.203125
T4 Overall	3.0692 +/- 6.5926'	0.20284 +/- 5.7367'	0.30078125
O2 Theta	-13.2068 +/- 24.1104'	1.2135 +/- 19.7864'	0.203125
O2 Alpha	-10.7554 +/- 20.4886'	0.96673 +/- 15.307'	0.203125
O2 Beta	-6.3833 +/- 12.8202'	0.67809 +/- 11.4798'	0.203125
O2 Delta	-15.3746 +/- 26.8835'	1.3924 +/- 21.7912'	0.203125
O2 Gamma	3.1233 +/- 4.8017'	1.0434 +/- 0.24661'	0.00390625
O2 Overall	1.1752 +/- 6.4051'	-0.93669 +/- 5.6042'	0.359375
Fp1 Theta	-6.4613 +/- 11.7724'	-5.6436 +/- 14.5087'	0.8203125
Fp1 Alpha	-5.2494 +/- 9.6817'	-4.0319 +/- 10.8357'	0.8203125
Fp1 Beta	-2.5847 +/- 6.0835'	-2.2663 +/- 7.8564'	1
Fp1 Delta	-7.7167 +/- 14.3276'	-6.6069 +/- 17.7835'	0.91015625
Fp1 Gamma	2.6713 +/- 2.3099'	1.3564 +/- 0.39207'	0.09765625
Fp1 Overall	-1.752 +/- 4.421'	-2.0483 +/- 2.7908'	0.91015625
C3 Theta	9.6325 +/- 21.8476'	-1.5415 +/- 15.1929'	0.359375
C3 Alpha	7.3295 +/- 15.4487'	-0.93597 +/- 11.5032'	0.359375
C3 Beta	6.7814 +/- 14.3278'	-0.15355 +/- 8.3969'	0.42578125
C3 Delta	11.1757 +/- 26.4174'	-1.6323 +/- 17.5461'	0.49609375
C3 Gamma	1.1272 +/- 1.6614'	1.6898 +/- 0.6791'	0.734375
C3 Overall	1.4238 +/- 4.2144'	0.34711 +/- 4.9042'	0.49609375
T3 Theta	20.8459 +/- 54.9947'	4.6367 +/- 12.0363'	0.91015625
T3 Alpha	16.5229 +/- 42.9367'	3.7768 +/- 9.1438'	0.8203125
T3 Beta	11.5948 +/- 28.738'	3.6211 +/- 7.2616'	0.91015625
T3 Delta	25.4483 +/- 68.4991'	5.3024 +/- 14.2394'	0.8203125
T3 Gamma	-0.94198 +/- 6.1223'	1.6364 +/- 0.46674'	0.09765625
T3 Overall	3.3901 +/- 4.859'	1.909 +/- 6.9675'	0.359375
O1 Theta	-2.7495 +/- 17.1427'	0.65271 +/- 28.8086'	1
O1 Alpha	-2.0957 +/- 13.9568'	0.64892 +/- 21.4488'	0.8203125
O1 Beta	-1.226 +/- 9.5359'	0.97378 +/- 16.1414'	0.91015625
O1 Delta	-3.1572 +/- 18.874'	0.21728 +/- 35.0435'	0.91015625
O1 Gamma	1.084 +/- 1.2943'	1.6256 +/- 1.0923'	0.734375
O1 Overall	1.353 +/- 4.6689'	0.044024 +/- 6.3288'	0.42578125
R hemi Theta	-16.7244 +/- 53.0667'	2.5368 +/- 7.5549'	0.30078125

R hemi Alpha	-14.6311 +/- 46.6077'	2.0833 +/- 5.6825'	0.359375
R hemi Beta	-7.2112 +/- 24.3645'	1.5733 +/- 4.2176'	0.30078125
R hemi Delta	-18.0279 +/- 59.2178'	2.8571 +/- 8.8378'	0.42578125
R hemi Gamma	5.0982 +/- 12.4531'	0.98572 +/- 0.47304'	0.09765625
R hemi Overall	0.83584 +/- 5.2025'	-0.4988 +/- 4.7612'	0.49609375
L hemi Theta	5.317 +/- 16.0133'	-0.4739 +/- 8.4564'	0.49609375
L hemi Alpha	4.1268 +/- 12.4357'	-0.13556 +/- 6.4339'	0.5703125
L hemi Beta	3.6414 +/- 8.6745'	0.54377 +/- 4.7383'	0.42578125
L hemi Delta	6.4374 +/- 19.631'	-0.67983 +/- 10.201'	0.42578125
L hemi Gamma	0.98516 +/- 1.7924'	1.5771 +/- 0.35924'	0.30078125
L hemi Overall	1.1037 +/- 4.0083'	0.062953 +/- 5.0083'	0.359375
Overall Theta	-5.7037 +/- 28.2429'	1.0314 +/- 5.9443'	0.734375
Overall Alpha	-5.2521 +/- 24.6106'	0.97391 +/- 4.5747'	0.734375
Overall Beta	-1.7849 +/- 13.3215'	1.0585 +/- 3.4403'	0.49609375
Overall Delta	-5.7954 +/- 31.7381'	1.0886 +/- 6.8119'	0.8203125
Overall Gamma	3.0417 +/- 6.3182'	1.2814 +/- 0.28797'	0.734375
Overall Overall	0.96979 +/- 4.4172'	-0.21794 +/- 4.709'	0.359375

Table S8. Hemispheric asymmetry, overall and within each band, treated at baseline vs. at 12 months.

Band	Baseline	12 Months	*p*
Frontal Theta	41.0888 +/- 137.9382'	-9.9898 +/- 21.7163'	0.49609375
Frontal Alpha	36.2265 +/- 121.5779'	-7.5524 +/- 16.1438'	0.49609375
Frontal Beta	16.7993 +/- 60.2644'	-5.1122 +/- 11.3833'	0.49609375
Frontal Delta	44.0713 +/- 152.0418'	-11.6691 +/- 26.2558'	0.49609375
Frontal Gamma	-10.9776 +/- 32.5008'	0.2117 +/- 0.73106'	0.25
Frontal Overall	0.17714 +/- 2.2727'	-0.51373 +/- 2.5535'	0.65234375
Parietal Theta	14.8442 +/- 59.4537'	-5.3001 +/- 19.11'	0.359375
Parietal Alpha	12.957 +/- 48.2804'	-4.0366 +/- 14.3672'	0.359375
Parietal Beta	9.8882 +/- 30.8952'	-2.2473 +/- 10.2565'	0.30078125
Parietal Delta	15.0364 +/- 72.5403'	-5.8433 +/- 22.5219'	0.42578125
Parietal Gamma	-1.1309 +/- 11.8212'	0.8617 +/- 1.2591'	0.42578125
Parietal Overall	0.39547 +/- 3.4069'	0.073989 +/- 3.5537'	0.91015625
Temporal Theta	21.7736 +/- 57.8009'	3.8076 +/- 14.8732'	0.734375
Temporal Alpha	17.1863 +/- 45.0771'	3.0311 +/- 11.436'	0.734375
Temporal Beta	11.5654 +/- 30.2151'	2.9456 +/- 9.1562'	1
Temporal Delta	26.5389 +/- 72.192'	4.5398 +/- 18.0382'	0.8203125
Temporal Gamma	-2.3044 +/- 6.4987'	0.70986 +/- 0.88492'	0.07421875
Temporal Overall	0.32091 +/- 4.4997'	1.7062 +/- 5.4437'	0.91015625
Occipital Theta	10.4573 +/- 23.8274'	-0.56087 +/- 27.8406'	0.09765625
Occipital Alpha	8.6597 +/- 20.5463'	-0.3178 +/- 20.7374'	0.09765625
Occipital Beta	5.1574 +/- 11.471'	0.29551 +/- 15.665'	0.12890625
Occipital Delta	12.2173 +/- 26.5856'	-1.175 +/- 33.3575'	0.09765625

Occipital Gamma	-2.0393 +/- 5.6575'	0.58221 +/- 1.1827'	0.09765625
Occipital Overall	0.17781 +/- 3.3702'	0.9807 +/- 3.5504'	0.734375
Overall Theta	88.1648 +/- 217.4111'	-12.0427 +/- 43.05'	0.1640625
Overall Alpha	75.0309 +/- 188.9366'	-8.8759 +/- 31.9156'	0.1640625
Overall Beta	43.4108 +/- 100.2333'	-4.1182 +/- 23.0267'	0.203125
Overall Delta	97.8631 +/- 245.1246'	-14.1473 +/- 53.4749'	0.203125
Overall Gamma	-16.4525 +/- 50.1066'	2.3654 +/- 2.446'	0.09765625
Overall Overall	1.0714 +/- 11.4666'	2.2472 +/- 10.4338'	1

Table S9. Inter-electrode coherence measures in the overall spectrum, treated at baseline vs. at 12 months.

Band	Baseline	12 Months	*p*
Fp2-C4 Theta	0.39488 +/- 0.19929'	0.4877 +/- 0.30732'	0.734375
Fp2-O2 Theta	0.43856 +/- 0.11658'	0.47656 +/- 0.27582'	0.91015625
Fp2-T4 Theta	0.3787 +/- 0.15994'	0.3127 +/- 0.21923'	0.12890625
Fp2-Fp1 Theta	0.46204 +/- 0.16753'	0.40403 +/- 0.26857'	0.25
Fp2-C3 Theta	0.34263 +/- 0.14834'	0.26735 +/- 0.10601'	0.42578125
Fp2-T3 Theta	0.37034 +/- 0.15419'	0.25104 +/- 0.10249'	0.203125
Fp2-O1 Theta	0.35844 +/- 0.13733'	0.31389 +/- 0.17544'	0.5703125
C4-O2 Theta	0.38617 +/- 0.22401'	0.40502 +/- 0.23058'	0.8203125
C4-T4 Theta	0.47847 +/- 0.28006'	0.46569 +/- 0.32496'	1
C4-Fp1 Theta	0.37821 +/- 0.17156'	0.31488 +/- 0.057842'	0.42578125
C4-C3 Theta	0.37856 +/- 0.1808'	0.26776 +/- 0.1138'	0.25
C4-T3 Theta	0.3059 +/- 0.12841'	0.23185 +/- 0.1016'	0.203125
C4-O1 Theta	0.31504 +/- 0.12947'	0.24027 +/- 0.088439'	0.203125
O2-T4 Theta	0.52955 +/- 0.27429'	0.41396 +/- 0.20807'	0.359375
O2-Fp1 Theta	0.39486 +/- 0.14794'	0.37292 +/- 0.17404'	0.65234375
O2-C3 Theta	0.35104 +/- 0.17727'	0.27888 +/- 0.10961'	0.25
O2-T3 Theta	0.33102 +/- 0.15356'	0.31042 +/- 0.10167'	0.8203125
O2-O1 Theta	0.3838 +/- 0.14064'	0.3835 +/- 0.20224'	0.91015625
T4-Fp1 Theta	0.37197 +/- 0.15796'	0.25123 +/- 0.084639'	0.1640625
T4-C3 Theta	0.33391 +/- 0.14912'	0.23466 +/- 0.12234'	0.1640625
T4-T3 Theta	0.34288 +/- 0.1397'	0.26663 +/- 0.1249'	0.359375
T4-O1 Theta	0.33074 +/- 0.15777'	0.28291 +/- 0.14088'	0.49609375
Fp1-C3 Theta	0.46992 +/- 0.20448'	0.37484 +/- 0.15863'	0.25
Fp1-T3 Theta	0.41061 +/- 0.19929'	0.32069 +/- 0.10722'	0.42578125
Fp1-O1 Theta	0.45349 +/- 0.16876'	0.40555 +/- 0.25097'	0.49609375
C3-T3 Theta	0.3873 +/- 0.24969'	0.3911 +/- 0.19017'	0.91015625
C3-O1 Theta	0.37202 +/- 0.16157'	0.28291 +/- 0.11877'	0.49609375
T3-O1 Theta	0.56078 +/- 0.20873'	0.50558 +/- 0.24393'	0.30078125
Fp2-C4 Alpha	0.36294 +/- 0.16378'	0.45278 +/- 0.31255'	1
Fp2-O2 Alpha	0.39244 +/- 0.13891'	0.45398 +/- 0.25289'	0.49609375
Fp2-T4 Alpha	0.33948 +/- 0.11008'	0.29749 +/- 0.17816'	0.42578125

Fp2-Fp1 Alpha	0.45438 +/- 0.18'	0.3756 +/- 0.23692'	0.203125
Fp2-C3 Alpha	0.3905 +/- 0.14723'	0.23675 +/- 0.11282'	0.0546875
Fp2-T3 Alpha	0.3966 +/- 0.16994'	0.25453 +/- 0.11872'	0.1640625
Fp2-O1 Alpha	0.37884 +/- 0.16095'	0.30513 +/- 0.13063'	0.30078125
C4-O2 Alpha	0.36323 +/- 0.18056'	0.40335 +/- 0.22984'	0.65234375
C4-T4 Alpha	0.42842 +/- 0.26768'	0.4317 +/- 0.33232'	0.91015625
C4-Fp1 Alpha	0.38936 +/- 0.1364'	0.28109 +/- 0.10645'	0.25
C4-C3 Alpha	0.40439 +/- 0.15915'	0.25158 +/- 0.12137'	0.01953125
C4-T3 Alpha	0.32015 +/- 0.152'	0.22474 +/- 0.12916'	0.25
C4-O1 Alpha	0.3411 +/- 0.15067'	0.2491 +/- 0.12196'	0.1640625
O2-T4 Alpha	0.4869 +/- 0.2249'	0.3966 +/- 0.22393'	0.49609375
O2-Fp1 Alpha	0.41211 +/- 0.13224'	0.33405 +/- 0.11998'	0.30078125
O2-C3 Alpha	0.40199 +/- 0.15325'	0.25965 +/- 0.097567'	0.0546875
O2-T3 Alpha	0.35822 +/- 0.15412'	0.29004 +/- 0.10088'	0.359375
O2-O1 Alpha	0.44852 +/- 0.13407'	0.38776 +/- 0.16487'	0.30078125
T4-Fp1 Alpha	0.36959 +/- 0.13917'	0.24944 +/- 0.11065'	0.09765625
T4-C3 Alpha	0.34685 +/- 0.10509'	0.22425 +/- 0.13115'	0.0546875
T4-T3 Alpha	0.33789 +/- 0.13989'	0.26807 +/- 0.15089'	0.359375
T4-O1 Alpha	0.34458 +/- 0.13552'	0.31002 +/- 0.18669'	0.65234375
Fp1-C3 Alpha	0.52185 +/- 0.14435'	0.40276 +/- 0.14175'	0.203125
Fp1-T3 Alpha	0.44769 +/- 0.20491'	0.33438 +/- 0.13156'	0.30078125
Fp1-O1 Alpha	0.55033 +/- 0.19656'	0.36868 +/- 0.22699'	0.09765625
C3-T3 Alpha	0.39806 +/- 0.27138'	0.42965 +/- 0.2014'	0.8203125
C3-O1 Alpha	0.46125 +/- 0.14901'	0.31452 +/- 0.16321'	0.12890625
T3-O1 Alpha	0.54853 +/- 0.21676'	0.53567 +/- 0.27481'	0.91015625
Fp2-C4 Beta	0.39884 +/- 0.16502'	0.41586 +/- 0.27326'	0.91015625
Fp2-O2 Beta	0.41875 +/- 0.15661'	0.4244 +/- 0.22727'	0.91015625
Fp2-T4 Beta	0.31545 +/- 0.10425'	0.27303 +/- 0.15414'	0.5703125
Fp2-Fp1 Beta	0.41227 +/- 0.15275'	0.362 +/- 0.15392'	0.49609375
Fp2-C3 Beta	0.37481 +/- 0.15004'	0.26387 +/- 0.12876'	0.203125
Fp2-T3 Beta	0.34644 +/- 0.15193'	0.29067 +/- 0.14085'	0.65234375
Fp2-O1 Beta	0.37369 +/- 0.15999'	0.31111 +/- 0.14105'	0.30078125
C4-O2 Beta	0.35361 +/- 0.15575'	0.39189 +/- 0.21512'	0.8203125
C4-T4 Beta	0.4194 +/- 0.25351'	0.44795 +/- 0.30815'	0.5703125
C4-Fp1 Beta	0.36532 +/- 0.1396'	0.28816 +/- 0.096975'	0.359375
C4-C3 Beta	0.39957 +/- 0.15548'	0.25297 +/- 0.13445'	0.0390625
C4-T3 Beta	0.29162 +/- 0.11246'	0.237 +/- 0.1267'	0.49609375
C4-O1 Beta	0.32332 +/- 0.11309'	0.27018 +/- 0.12294'	0.42578125
O2-T4 Beta	0.46368 +/- 0.21275'	0.40622 +/- 0.21194'	0.65234375
O2-Fp1 Beta	0.4436 +/- 0.13561'	0.29991 +/- 0.12251'	0.07421875
O2-C3 Beta	0.41894 +/- 0.14467'	0.29353 +/- 0.12566'	0.1640625
O2-T3 Beta	0.36537 +/- 0.11986'	0.32615 +/- 0.15418'	0.49609375
O2-O1 Beta	0.48129 +/- 0.16853'	0.39907 +/- 0.13943'	0.359375

T4-Fp1 Beta	0.38297 +/- 0.17006'	0.25048 +/- 0.11366'	0.12890625
T4-C3 Beta	0.33485 +/- 0.11546'	0.24062 +/- 0.15353'	0.12890625
T4-T3 Beta	0.34829 +/- 0.14201'	0.2704 +/- 0.16635'	0.42578125
T4-O1 Beta	0.3633 +/- 0.16246'	0.29772 +/- 0.19626'	0.42578125
Fp1-C3 Beta	0.53148 +/- 0.19572'	0.41887 +/- 0.14621'	0.359375
Fp1-T3 Beta	0.4125 +/- 0.18844'	0.37072 +/- 0.17346'	0.734375
Fp1-O1 Beta	0.60532 +/- 0.18987'	0.39611 +/- 0.21986'	0.09765625
C3-T3 Beta	0.36811 +/- 0.25124'	0.49482 +/- 0.23227'	0.12890625
C3-O1 Beta	0.49044 +/- 0.19706'	0.34199 +/- 0.16235'	0.42578125
T3-O1 Beta	0.48776 +/- 0.20844'	0.52323 +/- 0.29956'	0.734375
Fp2-C4 Delta	0.38658 +/- 0.20659'	0.48156 +/- 0.26819'	0.359375
Fp2-O2 Delta	0.35762 +/- 0.11589'	0.46589 +/- 0.24109'	0.203125
Fp2-T4 Delta	0.3575 +/- 0.1671'	0.31687 +/- 0.25126'	0.25
Fp2-Fp1 Delta	0.37929 +/- 0.15468'	0.39657 +/- 0.24208'	0.734375
Fp2-C3 Delta	0.32077 +/- 0.098154'	0.24948 +/- 0.092452'	0.25
Fp2-T3 Delta	0.3494 +/- 0.11964'	0.24738 +/- 0.10231'	0.0546875
Fp2-O1 Delta	0.32954 +/- 0.10037'	0.31842 +/- 0.17022'	0.91015625
C4-O2 Delta	0.31018 +/- 0.18546'	0.40914 +/- 0.15797'	0.12890625
C4-T4 Delta	0.49648 +/- 0.26963'	0.48818 +/- 0.34848'	0.91015625
C4-Fp1 Delta	0.32161 +/- 0.14282'	0.31715 +/- 0.082133'	0.5703125
C4-C3 Delta	0.3273 +/- 0.10663'	0.25443 +/- 0.10737'	0.07421875
C4-T3 Delta	0.27787 +/- 0.097859'	0.24383 +/- 0.12012'	0.49609375
C4-O1 Delta	0.27945 +/- 0.090114'	0.26491 +/- 0.13018'	0.65234375
O2-T4 Delta	0.49744 +/- 0.27169'	0.40139 +/- 0.21178'	0.65234375
O2-Fp1 Delta	0.29066 +/- 0.13282'	0.37567 +/- 0.1812'	0.09765625
O2-C3 Delta	0.29857 +/- 0.11478'	0.25398 +/- 0.067362'	0.30078125
O2-T3 Delta	0.26809 +/- 0.10749'	0.26585 +/- 0.092402'	0.91015625
O2-O1 Delta	0.31934 +/- 0.12508'	0.33294 +/- 0.18492'	0.8203125
T4-Fp1 Delta	0.29179 +/- 0.11469'	0.24025 +/- 0.090275'	0.359375
T4-C3 Delta	0.28371 +/- 0.10929'	0.23078 +/- 0.10605'	0.25
T4-T3 Delta	0.29063 +/- 0.091744'	0.23033 +/- 0.12613'	0.25
T4-O1 Delta	0.27892 +/- 0.11494'	0.29488 +/- 0.16025'	0.8203125
Fp1-C3 Delta	0.4286 +/- 0.19024'	0.35674 +/- 0.12247'	0.359375
Fp1-T3 Delta	0.35673 +/- 0.14212'	0.2805 +/- 0.090775'	0.25
Fp1-O1 Delta	0.40079 +/- 0.12875'	0.40638 +/- 0.24933'	0.91015625
C3-T3 Delta	0.3742 +/- 0.25903'	0.38972 +/- 0.20041'	0.734375
C3-O1 Delta	0.35831 +/- 0.13268'	0.28972 +/- 0.09552'	0.42578125
T3-O1 Delta	0.48392 +/- 0.1947'	0.43364 +/- 0.22177'	0.49609375
Fp2-C4 Gamma	0.44381 +/- 0.21927'	0.4505 +/- 0.27769'	0.91015625
Fp2-O2 Gamma	0.42621 +/- 0.18964'	0.39154 +/- 0.2013'	0.65234375
Fp2-T4 Gamma	0.3143 +/- 0.13286'	0.30502 +/- 0.19941'	0.734375
Fp2-Fp1 Gamma	0.38161 +/- 0.18441'	0.38662 +/- 0.2254'	1
Fp2-C3 Gamma	0.393 +/- 0.17849'	0.3128 +/- 0.16806'	0.30078125

Fp2-T3 Gamma	0.34346 +/- 0.20277'	0.31592 +/- 0.18116'	1
Fp2-O1 Gamma	0.3382 +/- 0.18652'	0.34019 +/- 0.20613'	1
C4-O2 Gamma	0.35171 +/- 0.1409'	0.39939 +/- 0.18859'	0.359375
C4-T4 Gamma	0.40289 +/- 0.25446'	0.47538 +/- 0.30121'	0.42578125
C4-Fp1 Gamma	0.31622 +/- 0.15506'	0.31188 +/- 0.11897'	1
C4-C3 Gamma	0.36325 +/- 0.13675'	0.28802 +/- 0.15349'	0.25
C4-T3 Gamma	0.28422 +/- 0.11551'	0.26743 +/- 0.13315'	1
C4-O1 Gamma	0.28338 +/- 0.12576'	0.28411 +/- 0.11631'	0.65234375
O2-T4 Gamma	0.46823 +/- 0.21849'	0.38681 +/- 0.17538'	0.65234375
O2-Fp1 Gamma	0.39842 +/- 0.13399'	0.2989 +/- 0.11947'	0.203125
O2-C3 Gamma	0.42637 +/- 0.14948'	0.27463 +/- 0.094455'	0.02734375
O2-T3 Gamma	0.38408 +/- 0.17961'	0.28047 +/- 0.093754'	0.30078125
O2-O1 Gamma	0.45333 +/- 0.19057'	0.38401 +/- 0.1255'	0.49609375
T4-Fp1 Gamma	0.34652 +/- 0.1068'	0.26777 +/- 0.19241'	0.25
T4-C3 Gamma	0.33107 +/- 0.085006'	0.25548 +/- 0.19067'	0.359375
T4-T3 Gamma	0.37 +/- 0.13339'	0.26703 +/- 0.18753'	0.203125
T4-O1 Gamma	0.3347 +/- 0.11965'	0.3001 +/- 0.21919'	0.49609375
Fp1-C3 Gamma	0.50211 +/- 0.20083'	0.46708 +/- 0.23113'	0.8203125
Fp1-T3 Gamma	0.37815 +/- 0.20485'	0.44288 +/- 0.18082'	0.49609375
Fp1-O1 Gamma	0.47778 +/- 0.16781'	0.45199 +/- 0.23509'	0.8203125
C3-T3 Gamma	0.44613 +/- 0.27008'	0.51391 +/- 0.26541'	0.49609375
C3-O1 Gamma	0.44283 +/- 0.16621'	0.403 +/- 0.21339'	1
T3-O1 Gamma	0.46218 +/- 0.20809'	0.5187 +/- 0.31328'	0.734375
Fp2-C4 Overall	0.40873 +/- 0.1817'	0.45691 +/- 0.25707'	1
Fp2-O2 Overall	0.40186 +/- 0.11018'	0.40711 +/- 0.17137'	0.91015625
Fp2-T4 Overall	0.31385 +/- 0.095162'	0.29806 +/- 0.15306'	0.8203125
Fp2-Fp1 Overall	0.41377 +/- 0.10126'	0.39969 +/- 0.19445'	0.8203125
Fp2-C3 Overall	0.37304 +/- 0.11035'	0.29405 +/- 0.13555'	0.359375
Fp2-T3 Overall	0.36194 +/- 0.14128'	0.29902 +/- 0.14535'	0.5703125
Fp2-O1 Overall	0.35476 +/- 0.11247'	0.34769 +/- 0.13918'	1
C4-O2 Overall	0.34112 +/- 0.1397'	0.38407 +/- 0.16788'	0.42578125
C4-T4 Overall	0.43081 +/- 0.25955'	0.45379 +/- 0.27232'	0.91015625
C4-Fp1 Overall	0.33701 +/- 0.11294'	0.31492 +/- 0.086371'	1
C4-C3 Overall	0.37598 +/- 0.13841'	0.2887 +/- 0.13999'	0.25
C4-T3 Overall	0.30556 +/- 0.10446'	0.27426 +/- 0.13591'	0.49609375
C4-O1 Overall	0.29068 +/- 0.10042'	0.29915 +/- 0.13311'	0.734375
O2-T4 Overall	0.47982 +/- 0.20184'	0.39028 +/- 0.14332'	0.49609375
O2-Fp1 Overall	0.40458 +/- 0.11861'	0.30889 +/- 0.094482'	0.203125
O2-C3 Overall	0.40377 +/- 0.12793'	0.26681 +/- 0.099421'	0.09765625
O2-T3 Overall	0.37888 +/- 0.1255'	0.27703 +/- 0.081127'	0.203125
O2-O1 Overall	0.46893 +/- 0.16667'	0.39276 +/- 0.12981'	0.25
T4-Fp1 Overall	0.3335 +/- 0.11186'	0.25466 +/- 0.11214'	0.12890625
T4-C3 Overall	0.33531 +/- 0.10693'	0.24504 +/- 0.15191'	0.203125

T4-T3 Overall	0.35537 +/- 0.12808'	0.26191 +/- 0.15273'	0.203125
T4-O1 Overall	0.34664 +/- 0.095155'	0.29614 +/- 0.17026'	0.5703125
Fp1-C3 Overall	0.49978 +/- 0.18802'	0.41227 +/- 0.12452'	0.25
Fp1-T3 Overall	0.38379 +/- 0.13499'	0.37269 +/- 0.10512'	0.91015625
Fp1-O1 Overall	0.47094 +/- 0.13709'	0.40335 +/- 0.17371'	0.734375
C3-T3 Overall	0.45252 +/- 0.2586'	0.44756 +/- 0.21416'	1
C3-O1 Overall	0.43662 +/- 0.15782'	0.35549 +/- 0.1491'	0.42578125
T3-O1 Overall	0.48316 +/- 0.17354'	0.51082 +/- 0.29561'	0.8203125

Table S10. Overall power and power within each band at each electrode location, untreated at baseline vs. at 12 months.

Band	Baseline	12 Months	*p*
Fp2 Theta	-1.6147 +/- 6.5254'	-4.5294 +/- 4.7287'	0.07421875
Fp2 Alpha	-1.1731 +/- 5.0021'	-3.0898 +/- 3.2086'	0.07421875
Fp2 Beta	-1.4207 +/- 4.1829'	-2.3148 +/- 2.8251'	0.09765625
Fp2 Delta	-2.4492 +/- 8.624'	-6.321 +/- 6.182'	0.07421875
Fp2 Gamma	0.73436 +/- 0.55074'	0.97169 +/- 0.34955'	0.25
Fp2 Overall	-3.0949 +/- 4.1132'	-4.604 +/- 1.9025'	0.91015625
C4 Theta	-2.1315 +/- 16.3853'	-12.6099 +/- 14.725'	0.25
C4 Alpha	-1.382 +/- 12.4684'	-9.3185 +/- 11.1534'	0.30078125
C4 Beta	-1.2585 +/- 9.5609'	-8.5623 +/- 10.4676'	0.42578125
C4 Delta	-3.0252 +/- 20.1411'	-17.2557 +/- 20.5873'	0.5703125
C4 Gamma	1.1893 +/- 1.09'	-0.42995 +/- 2.0711'	0.42578125
C4 Overall	-3.2215 +/- 4.8507'	-3.9997 +/- 3.5107'	0.359375
T4 Theta	2.5536 +/- 13.1519'	109.694 +/- 315.0896'	0.07421875
T4 Alpha	1.9504 +/- 10.1534'	94.4001 +/- 273.0846'	0.07421875
T4 Beta	1.4846 +/- 8.3281'	93.7959 +/- 270.4052'	0.09765625
T4 Delta	2.7077 +/- 17.2825'	114.8333 +/- 324.1421'	0.0390625
T4 Gamma	0.98807 +/- 0.5479'	18.8093 +/- 51.5697'	0.09765625
T4 Overall	-0.54291 +/- 3.8246'	-2.0923 +/- 3.254'	0.09765625
O2 Theta	4.0086 +/- 8.1257'	-41.3183 +/- 107.6733'	0.02734375
O2 Alpha	3.1333 +/- 6.0662'	-29.2041 +/- 75.0885'	0.02734375
O2 Beta	2.3972 +/- 4.4152'	-25.2616 +/- 66.1963'	0.0390625
O2 Delta	4.6939 +/- 10.4838'	-51.6634 +/- 135.8023'	0.0390625
O2 Gamma	0.93962 +/- 0.45299'	-0.40508 +/- 3.6966'	0.8203125
O2 Overall	-2.5319 +/- 4.6894'	-4.2626 +/- 3.1'	0.1640625
Fp1 Theta	4.2463 +/- 18.4689'	-2.8628 +/- 1.7287'	0.734375
Fp1 Alpha	3.7944 +/- 14.4406'	-1.8982 +/- 1.2077'	0.359375
Fp1 Beta	3.5336 +/- 13.0959'	-1.2907 +/- 1.0829'	0.734375
Fp1 Delta	5.173 +/- 25.3602'	-3.9248 +/- 2.417'	0.8203125
Fp1 Gamma	1.5986 +/- 2.0775'	1.0446 +/- 0.16285'	0.8203125
Fp1 Overall	-2.5234 +/- 3.632'	-5.8652 +/- 2.1439'	0.00390625
C3 Theta	-17.3424 +/- 27.7711'	5.1215 +/- 21.1862'	0.30078125

C3 Alpha	-12.9029 +/- 20.9959'	3.8199 +/- 15.3817'	0.30078125
C3 Beta	-11.1367 +/- 18.3301'	4.0642 +/- 15.2576'	0.49609375
C3 Delta	-25.7377 +/- 40.7283'	4.5737 +/- 22.6244'	0.25
C3 Gamma	0.35489 +/- 1.2033'	2.5605 +/- 4.9571'	0.09765625
C3 Overall	-2.354 +/- 4.9664'	-4.0909 +/- 4.3529'	0.25
T3 Theta	9.3832 +/- 17.7964'	3.8169 +/- 10.7411'	0.91015625
T3 Alpha	7.8688 +/- 14.4523'	3.0475 +/- 8.3598'	1
T3 Beta	7.0127 +/- 12.8476'	3.4279 +/- 9.124'	1
T3 Delta	12.5056 +/- 25.2996'	5.6136 +/- 15.966'	0.91015625
T3 Gamma	1.0116 +/- 0.68677'	1.6016 +/- 1.3299'	0.49609375
T3 Overall	-0.87694 +/- 4.5616'	-1.6894 +/- 4.1151'	0.42578125
O1 Theta	-4.4477 +/- 12.5136'	-3.0936 +/- 6.9851'	1
O1 Alpha	-3.2576 +/- 9.8101'	-2.0775 +/- 5.319'	1
O1 Beta	-2.4238 +/- 7.511'	-1.3274 +/- 4.3119'	1
O1 Delta	-6.4055 +/- 15.8787'	-4.4437 +/- 8.4833'	0.91015625
O1 Gamma	1.0067 +/- 0.37125'	0.92084 +/- 0.41037'	0.42578125
O1 Overall	-3.0215 +/- 4.7714'	-4.0077 +/- 3.1238'	0.65234375
R hemi Theta	0.70404 +/- 9.1051'	12.8093 +/- 85.5026'	0.42578125
R hemi Alpha	0.6322 +/- 6.8814'	13.1967 +/- 72.1623'	0.49609375
R hemi Beta	0.30059 +/- 5.3654'	14.4142 +/- 70.5478'	0.49609375
R hemi Delta	0.48182 +/- 11.7749'	9.8981 +/- 91.0119'	0.5703125
R hemi Gamma	0.96284 +/- 0.4775'	4.7365 +/- 12.6305'	0.91015625
R hemi Overall	-2.3478 +/- 4.093'	-3.7396 +/- 2.5473'	0.12890625
L hemi Theta	-2.0402 +/- 9.5772'	0.74554 +/- 7.1952'	0.91015625
L hemi Alpha	-1.1244 +/- 7.1953'	0.72299 +/- 5.3994'	0.91015625
L hemi Beta	-0.75354 +/- 5.8077'	1.2185 +/- 5.1383'	0.734375
L hemi Delta	-3.616 +/- 13.4631'	0.4547 +/- 7.9624'	0.8203125
L hemi Gamma	0.99295 +/- 0.65462'	1.5319 +/- 1.2919'	0.203125
L hemi Overall	-2.194 +/- 4.1027'	-3.9133 +/- 2.9704'	0.12890625
Overall Theta	-0.66814 +/- 5.6427'	6.7769 +/- 46.0782'	0.91015625
Overall Alpha	-0.24609 +/- 4.183'	6.9598 +/- 38.5928'	0.91015625
Overall Beta	-0.22646 +/- 3.1596'	7.8161 +/- 37.6269'	1
Overall Delta	-1.5671 +/- 8.115'	5.1762 +/- 49.0286'	0.91015625
Overall Gamma	0.97789 +/- 0.45065'	3.1342 +/- 6.9404'	0.91015625
Overall Overall	-2.2709 +/- 4.0148'	-3.8265 +/- 2.7047'	0.09765625

Table S11. Hemispheric asymmetry, overall and within each band, untreated at baseline vs. at 12 months.

Band	Baseline	12 Months	*p*
Frontal Theta	5.8608 +/- 21.203'	1.6665 +/- 3.4764'	0.91015625
Frontal Alpha	4.9675 +/- 16.5359'	1.1917 +/- 2.4171'	0.91015625
Frontal Beta	4.9543 +/- 14.8929'	1.0241 +/- 2.2472'	1
Frontal Delta	7.6225 +/- 28.7263'	2.3962 +/- 4.4571'	0.91015625

Frontal Gamma	0.86421 +/- 2.1103'	0.07288 +/- 0.32701'	0.359375
Frontal Overall	0.57149 +/- 2.4073'	-1.2613 +/- 1.3139'	0.12890625
Parietal Theta	-15.2109 +/- 36.0135'	17.7314 +/- 24.4903'	0.12890625
Parietal Alpha	-11.5209 +/- 27.2849'	13.1384 +/- 18.2628'	0.07421875
Parietal Beta	-9.8781 +/- 22.7982'	12.6266 +/- 17.7858'	0.07421875
Parietal Delta	-22.7124 +/- 49.1711'	21.8295 +/- 27.9365'	0.12890625
Parietal Gamma	-0.83442 +/- 1.4105'	2.9905 +/- 5.6949'	0.00390625
Parietal Overall	0.8676 +/- 2.445'	-0.091209 +/- 2.4644'	0.30078125
Temporal Theta	6.8296 +/- 16.1171'	-105.8772 +/- 314.0665'	0.203125
Temporal Alpha	5.9183 +/- 13.4189'	-91.3527 +/- 272.1884'	0.203125
Temporal Beta	5.5281 +/- 12.6068'	-90.3684 +/- 269.7723'	0.203125
Temporal Delta	9.7977 +/- 23.7658'	-109.2194 +/- 323.4772'	0.203125
Temporal Gamma	0.023562 +/- 0.53226'	-17.2078 +/- 51.544'	0.30078125
Temporal Overall	-0.33404 +/- 2.4852'	0.40283 +/- 1.8606'	0.42578125
Occipital Theta	-8.4563 +/- 17.5338'	38.2249 +/- 103.0521'	0.01953125
Occipital Alpha	-6.3909 +/- 13.3715'	27.1274 +/- 71.6988'	0.01953125
Occipital Beta	-4.821 +/- 10.1408'	23.9342 +/- 63.2968'	0.01171875
Occipital Delta	-11.0994 +/- 22.6268'	47.2197 +/- 130.0555'	0.0546875
Occipital Gamma	0.067086 +/- 0.3357'	1.3258 +/- 3.6129'	1
Occipital Overall	-0.48964 +/- 2.4748'	0.2549 +/- 1.8129'	0.42578125
Overall Theta	-10.9771 +/- 59.585'	-48.2534 +/- 315.753'	0.42578125
Overall Alpha	-7.026 +/- 45.3005'	-49.8963 +/- 268.7922'	0.42578125
Overall Beta	-4.2166 +/- 36.9007'	-52.7828 +/- 263.6175'	0.42578125
Overall Delta	-16.3921 +/- 77.6038'	-37.7745 +/- 336.5284'	0.42578125
Overall Gamma	0.12043 +/- 2.8304'	-12.8188 +/- 45.5572'	0.8203125
Overall Overall	0.61544 +/- 6.5684'	-0.69472 +/- 4.6668'	0.42578125

Table S12. Inter-electrode coherence measures in the overall spectrum, untreated at baseline vs. at 12 months.

Band	Baseline	12 Months	*p*
Fp2-C4 Theta	0.27081 +/- 0.13146'	0.36682 +/- 0.24738'	0.359375
Fp2-O2 Theta	0.2562 +/- 0.11246'	0.3651 +/- 0.24987'	0.734375
Fp2-T4 Theta	0.21967 +/- 0.10383'	0.29357 +/- 0.23141'	1
Fp2-Fp1 Theta	0.32464 +/- 0.21667'	0.34769 +/- 0.26482'	0.734375
Fp2-C3 Theta	0.22099 +/- 0.11575'	0.28589 +/- 0.1489'	0.359375
Fp2-T3 Theta	0.22343 +/- 0.11094'	0.29253 +/- 0.17014'	0.42578125
Fp2-O1 Theta	0.22965 +/- 0.11807'	0.2656 +/- 0.13721'	0.5703125
C4-O2 Theta	0.24777 +/- 0.16256'	0.36346 +/- 0.236'	0.203125
C4-T4 Theta	0.36583 +/- 0.27638'	0.32334 +/- 0.21726'	0.65234375
C4-Fp1 Theta	0.22879 +/- 0.20095'	0.26451 +/- 0.13775'	0.25
C4-C3 Theta	0.26036 +/- 0.22457'	0.33329 +/- 0.13861'	0.203125
C4-T3 Theta	0.24425 +/- 0.23585'	0.29444 +/- 0.17917'	0.25
C4-O1 Theta	0.23397 +/- 0.24084'	0.22884 +/- 0.10157'	0.203125

O2-T4 Theta	0.48706 +/- 0.28564'	0.44796 +/- 0.23942'	0.734375
O2-Fp1 Theta	0.23392 +/- 0.17623'	0.26743 +/- 0.11093'	0.359375
O2-C3 Theta	0.25166 +/- 0.23694'	0.29048 +/- 0.17897'	0.30078125
O2-T3 Theta	0.25506 +/- 0.2'	0.28452 +/- 0.17145'	0.5703125
O2-O1 Theta	0.29043 +/- 0.21475'	0.36102 +/- 0.2122'	0.359375
T4-Fp1 Theta	0.21943 +/- 0.20157'	0.26789 +/- 0.1569'	0.25
T4-C3 Theta	0.24151 +/- 0.24867'	0.27096 +/- 0.18388'	0.359375
T4-T3 Theta	0.25456 +/- 0.25251'	0.3258 +/- 0.16093'	0.12890625
T4-O1 Theta	0.23896 +/- 0.24031'	0.26737 +/- 0.16943'	0.25
Fp1-C3 Theta	0.40263 +/- 0.23171'	0.39525 +/- 0.21459'	0.91015625
Fp1-T3 Theta	0.30963 +/- 0.18596'	0.3134 +/- 0.19248'	0.91015625
Fp1-O1 Theta	0.35818 +/- 0.18605'	0.33325 +/- 0.13769'	0.91015625
C3-T3 Theta	0.41834 +/- 0.24009'	0.365 +/- 0.24501'	0.65234375
C3-O1 Theta	0.35346 +/- 0.23653'	0.33251 +/- 0.18279'	1
T3-O1 Theta	0.39065 +/- 0.2298'	0.41087 +/- 0.21321'	0.91015625
Fp2-C4 Alpha	0.30604 +/- 0.1634'	0.38901 +/- 0.19245'	0.49609375
Fp2-O2 Alpha	0.31621 +/- 0.15267'	0.3991 +/- 0.27182'	0.734375
Fp2-T4 Alpha	0.26776 +/- 0.15927'	0.33401 +/- 0.26466'	0.91015625
Fp2-Fp1 Alpha	0.36291 +/- 0.19476'	0.3616 +/- 0.27758'	1
Fp2-C3 Alpha	0.26308 +/- 0.17089'	0.32741 +/- 0.26468'	0.5703125
Fp2-T3 Alpha	0.26215 +/- 0.17566'	0.31572 +/- 0.25222'	0.49609375
Fp2-O1 Alpha	0.25868 +/- 0.17987'	0.29144 +/- 0.19382'	0.65234375
C4-O2 Alpha	0.28159 +/- 0.20222'	0.35822 +/- 0.21868'	0.359375
C4-T4 Alpha	0.39223 +/- 0.25501'	0.35371 +/- 0.21468'	0.8203125
C4-Fp1 Alpha	0.26002 +/- 0.23575'	0.31927 +/- 0.21564'	0.49609375
C4-C3 Alpha	0.29576 +/- 0.24432'	0.34969 +/- 0.17888'	0.359375
C4-T3 Alpha	0.25324 +/- 0.24129'	0.29939 +/- 0.20752'	0.42578125
C4-O1 Alpha	0.24384 +/- 0.25411'	0.28717 +/- 0.2005'	0.42578125
O2-T4 Alpha	0.43358 +/- 0.29428'	0.47503 +/- 0.28516'	0.91015625
O2-Fp1 Alpha	0.28589 +/- 0.21357'	0.30109 +/- 0.19959'	0.734375
O2-C3 Alpha	0.28256 +/- 0.24391'	0.3076 +/- 0.24042'	0.42578125
O2-T3 Alpha	0.26456 +/- 0.2127'	0.29354 +/- 0.23201'	1
O2-O1 Alpha	0.3124 +/- 0.23773'	0.38545 +/- 0.25847'	0.5703125
T4-Fp1 Alpha	0.26505 +/- 0.24066'	0.30578 +/- 0.23906'	0.8203125
T4-C3 Alpha	0.2505 +/- 0.25472'	0.29726 +/- 0.24995'	0.65234375
T4-T3 Alpha	0.25983 +/- 0.25129'	0.33271 +/- 0.21011'	0.359375
T4-O1 Alpha	0.24715 +/- 0.23395'	0.29087 +/- 0.23666'	0.8203125
Fp1-C3 Alpha	0.36035 +/- 0.24447'	0.39945 +/- 0.22008'	0.8203125
Fp1-T3 Alpha	0.30951 +/- 0.22575'	0.33129 +/- 0.22615'	0.65234375
Fp1-O1 Alpha	0.34291 +/- 0.23205'	0.39871 +/- 0.1834'	0.42578125
C3-T3 Alpha	0.44316 +/- 0.20254'	0.40546 +/- 0.29913'	0.734375
C3-O1 Alpha	0.33634 +/- 0.25694'	0.35477 +/- 0.23514'	0.8203125
T3-O1 Alpha	0.42092 +/- 0.24519'	0.42569 +/- 0.25578'	0.91015625

Fp2-C4 Beta	0.3003 +/- 0.15513'	0.3984 +/- 0.17589'	0.30078125
Fp2-O2 Beta	0.3244 +/- 0.17342'	0.3848 +/- 0.238'	0.734375
Fp2-T4 Beta	0.27461 +/- 0.18194'	0.32683 +/- 0.2301'	0.91015625
Fp2-Fp1 Beta	0.34777 +/- 0.23594'	0.32204 +/- 0.22687'	0.91015625
Fp2-C3 Beta	0.25122 +/- 0.1555'	0.31446 +/- 0.24829'	0.65234375
Fp2-T3 Beta	0.2361 +/- 0.16127'	0.27122 +/- 0.20212'	0.49609375
Fp2-O1 Beta	0.23949 +/- 0.16607'	0.27238 +/- 0.18265'	0.5703125
C4-O2 Beta	0.31047 +/- 0.19734'	0.36309 +/- 0.17577'	0.42578125
C4-T4 Beta	0.42289 +/- 0.23603'	0.41282 +/- 0.23412'	0.8203125
C4-Fp1 Beta	0.24778 +/- 0.2208'	0.31525 +/- 0.20217'	0.5703125
C4-C3 Beta	0.28995 +/- 0.22747'	0.32063 +/- 0.16803'	0.49609375
C4-T3 Beta	0.23729 +/- 0.22478'	0.24947 +/- 0.14502'	0.49609375
C4-O1 Beta	0.23973 +/- 0.23961'	0.29541 +/- 0.19087'	0.30078125
O2-T4 Beta	0.39607 +/- 0.29202'	0.42046 +/- 0.28987'	1
O2-Fp1 Beta	0.31543 +/- 0.21805'	0.27607 +/- 0.15506'	0.91015625
O2-C3 Beta	0.30244 +/- 0.22779'	0.29178 +/- 0.20122'	0.5703125
O2-T3 Beta	0.26022 +/- 0.1949'	0.26518 +/- 0.18819'	0.91015625
O2-O1 Beta	0.32854 +/- 0.24132'	0.37253 +/- 0.22341'	0.5703125
T4-Fp1 Beta	0.27069 +/- 0.23652'	0.27274 +/- 0.20269'	0.91015625
T4-C3 Beta	0.24353 +/- 0.23416'	0.25719 +/- 0.21168'	0.91015625
T4-T3 Beta	0.25883 +/- 0.23453'	0.29604 +/- 0.18457'	0.8203125
T4-O1 Beta	0.24362 +/- 0.21351'	0.26359 +/- 0.19106'	0.49609375
Fp1-C3 Beta	0.30873 +/- 0.23812'	0.33896 +/- 0.17125'	0.734375
Fp1-T3 Beta	0.26898 +/- 0.20735'	0.27811 +/- 0.14343'	0.734375
Fp1-O1 Beta	0.29022 +/- 0.22298'	0.3348 +/- 0.13533'	0.359375
C3-T3 Beta	0.42704 +/- 0.23481'	0.43683 +/- 0.23529'	1
C3-O1 Beta	0.33661 +/- 0.27029'	0.32869 +/- 0.21663'	0.734375
T3-O1 Beta	0.44111 +/- 0.27799'	0.37922 +/- 0.22598'	0.65234375
Fp2-C4 Delta	0.25384 +/- 0.10911'	0.35407 +/- 0.2463'	0.42578125
Fp2-O2 Delta	0.22134 +/- 0.063843'	0.36229 +/- 0.25946'	0.25
Fp2-T4 Delta	0.20871 +/- 0.081154'	0.2607 +/- 0.22575'	0.734375
Fp2-Fp1 Delta	0.33567 +/- 0.21601'	0.35301 +/- 0.23469'	0.8203125
Fp2-C3 Delta	0.19466 +/- 0.069298'	0.275 +/- 0.099888'	0.09765625
Fp2-T3 Delta	0.19499 +/- 0.068755'	0.23817 +/- 0.10332'	0.30078125
Fp2-O1 Delta	0.21736 +/- 0.070945'	0.25555 +/- 0.10617'	0.359375
C4-O2 Delta	0.27375 +/- 0.198'	0.36913 +/- 0.23217'	0.49609375
C4-T4 Delta	0.38064 +/- 0.27231'	0.35356 +/- 0.19108'	1
C4-Fp1 Delta	0.25119 +/- 0.18678'	0.29053 +/- 0.10659'	0.30078125
C4-C3 Delta	0.27845 +/- 0.24598'	0.34162 +/- 0.13116'	0.203125
C4-T3 Delta	0.25597 +/- 0.24111'	0.28874 +/- 0.15294'	0.25
C4-O1 Delta	0.24757 +/- 0.24862'	0.23867 +/- 0.09093'	0.30078125
O2-T4 Delta	0.49469 +/- 0.26824'	0.35067 +/- 0.24107'	0.203125
O2-Fp1 Delta	0.25313 +/- 0.14974'	0.26183 +/- 0.092159'	0.30078125

O2-C3 Delta	0.25584 +/- 0.23974'	0.31377 +/- 0.13597'	0.12890625
O2-T3 Delta	0.26421 +/- 0.21681'	0.25199 +/- 0.14335'	0.8203125
O2-O1 Delta	0.30735 +/- 0.21853'	0.36149 +/- 0.16203'	0.49609375
T4-Fp1 Delta	0.23313 +/- 0.16714'	0.25588 +/- 0.098832'	0.42578125
T4-C3 Delta	0.24692 +/- 0.24394'	0.28282 +/- 0.13776'	0.203125
T4-T3 Delta	0.2704 +/- 0.23163'	0.28229 +/- 0.12105'	0.30078125
T4-O1 Delta	0.24945 +/- 0.2363'	0.23857 +/- 0.1166'	0.42578125
Fp1-C3 Delta	0.39746 +/- 0.20359'	0.33413 +/- 0.12178'	0.5703125
Fp1-T3 Delta	0.28757 +/- 0.16172'	0.25534 +/- 0.11661'	1
Fp1-O1 Delta	0.33271 +/- 0.17065'	0.25521 +/- 0.0924'	0.49609375
C3-T3 Delta	0.39404 +/- 0.24395'	0.35617 +/- 0.21558'	0.91015625
C3-O1 Delta	0.38998 +/- 0.26474'	0.29129 +/- 0.15857'	0.49609375
T3-O1 Delta	0.39759 +/- 0.23761'	0.35889 +/- 0.23647'	0.65234375
Fp2-C4 Gamma	0.29701 +/- 0.14952'	0.38127 +/- 0.20789'	0.42578125
Fp2-O2 Gamma	0.30599 +/- 0.18219'	0.34242 +/- 0.19967'	0.734375
Fp2-T4 Gamma	0.26523 +/- 0.18757'	0.27039 +/- 0.16307'	0.8203125
Fp2-Fp1 Gamma	0.31348 +/- 0.23787'	0.23171 +/- 0.15786'	1
Fp2-C3 Gamma	0.24117 +/- 0.16529'	0.25352 +/- 0.1683'	1
Fp2-T3 Gamma	0.20881 +/- 0.14586'	0.18563 +/- 0.084138'	0.91015625
Fp2-O1 Gamma	0.23032 +/- 0.14994'	0.21739 +/- 0.12311'	0.91015625
C4-O2 Gamma	0.32693 +/- 0.20578'	0.33671 +/- 0.14095'	0.91015625
C4-T4 Gamma	0.47014 +/- 0.25557'	0.40544 +/- 0.20904'	0.65234375
C4-Fp1 Gamma	0.25291 +/- 0.20727'	0.24208 +/- 0.15621'	0.8203125
C4-C3 Gamma	0.29807 +/- 0.22248'	0.29427 +/- 0.19081'	0.734375
C4-T3 Gamma	0.23451 +/- 0.20803'	0.19322 +/- 0.071853'	0.91015625
C4-O1 Gamma	0.27034 +/- 0.22834'	0.25586 +/- 0.12924'	0.359375
O2-T4 Gamma	0.37653 +/- 0.29106'	0.35851 +/- 0.24281'	1
O2-Fp1 Gamma	0.29704 +/- 0.21436'	0.2099 +/- 0.065715'	0.49609375
O2-C3 Gamma	0.2937 +/- 0.22059'	0.27039 +/- 0.17969'	1
O2-T3 Gamma	0.24197 +/- 0.18273'	0.22625 +/- 0.12394'	0.8203125
O2-O1 Gamma	0.3526 +/- 0.22675'	0.36763 +/- 0.19165'	0.65234375
T4-Fp1 Gamma	0.26349 +/- 0.22198'	0.17655 +/- 0.051623'	0.65234375
T4-C3 Gamma	0.24723 +/- 0.22796'	0.21022 +/- 0.14394'	0.8203125
T4-T3 Gamma	0.25569 +/- 0.2226'	0.21486 +/- 0.10027'	1
T4-O1 Gamma	0.2546 +/- 0.19428'	0.22623 +/- 0.10765'	0.65234375
Fp1-C3 Gamma	0.27814 +/- 0.19572'	0.27847 +/- 0.14423'	0.91015625
Fp1-T3 Gamma	0.23976 +/- 0.18735'	0.1983 +/- 0.031997'	0.91015625
Fp1-O1 Gamma	0.2709 +/- 0.19947'	0.22734 +/- 0.040902'	0.91015625
C3-T3 Gamma	0.45628 +/- 0.26072'	0.5295 +/- 0.16844'	0.42578125
C3-O1 Gamma	0.37106 +/- 0.23455'	0.33676 +/- 0.20308'	0.65234375
T3-O1 Gamma	0.40525 +/- 0.18309'	0.33567 +/- 0.1484'	0.49609375
Fp2-C4 Overall	0.32037 +/- 0.13853'	0.37512 +/- 0.17018'	0.8203125
Fp2-O2 Overall	0.3057 +/- 0.14717'	0.3595 +/- 0.16046'	0.65234375

Fp2-T4 Overall	0.28277 +/- 0.17061'	0.28749 +/- 0.16097'	1
Fp2-Fp1 Overall	0.34569 +/- 0.19498'	0.31501 +/- 0.2122'	1
Fp2-C3 Overall	0.26211 +/- 0.15499'	0.3057 +/- 0.20421'	0.65234375
Fp2-T3 Overall	0.24555 +/- 0.12504'	0.26276 +/- 0.14748'	0.734375
Fp2-O1 Overall	0.25406 +/- 0.12752'	0.2834 +/- 0.14961'	0.42578125
C4-O2 Overall	0.33461 +/- 0.1799'	0.37566 +/- 0.15939'	0.734375
C4-T4 Overall	0.44938 +/- 0.20759'	0.41274 +/- 0.14204'	0.8203125
C4-Fp1 Overall	0.28808 +/- 0.19071'	0.2757 +/- 0.16829'	1
C4-C3 Overall	0.33706 +/- 0.22075'	0.32477 +/- 0.19284'	1
C4-T3 Overall	0.28804 +/- 0.20593'	0.25515 +/- 0.13333'	1
C4-O1 Overall	0.30301 +/- 0.21602'	0.28093 +/- 0.13939'	0.65234375
O2-T4 Overall	0.44992 +/- 0.25961'	0.39641 +/- 0.2121'	0.8203125
O2-Fp1 Overall	0.3215 +/- 0.20927'	0.27378 +/- 0.099554'	0.91015625
O2-C3 Overall	0.32003 +/- 0.22882'	0.30848 +/- 0.20679'	0.91015625
O2-T3 Overall	0.28861 +/- 0.18524'	0.2839 +/- 0.159'	0.65234375
O2-O1 Overall	0.37464 +/- 0.22163'	0.40015 +/- 0.1752'	0.203125
T4-Fp1 Overall	0.29601 +/- 0.22197'	0.24623 +/- 0.11419'	0.734375
T4-C3 Overall	0.27987 +/- 0.25224'	0.25229 +/- 0.17248'	0.734375
T4-T3 Overall	0.29054 +/- 0.22619'	0.26896 +/- 0.12821'	0.91015625
T4-O1 Overall	0.28004 +/- 0.19697'	0.2784 +/- 0.14173'	1
Fp1-C3 Overall	0.36781 +/- 0.18441'	0.33954 +/- 0.14978'	0.5703125
Fp1-T3 Overall	0.2958 +/- 0.18691'	0.26092 +/- 0.096722'	0.734375
Fp1-O1 Overall	0.33653 +/- 0.19107'	0.30422 +/- 0.1042'	0.8203125
C3-T3 Overall	0.49174 +/- 0.19806'	0.50115 +/- 0.12092'	0.91015625
C3-O1 Overall	0.40603 +/- 0.20104'	0.35397 +/- 0.20924'	0.49609375
T3-O1 Overall	0.44955 +/- 0.15809'	0.41007 +/- 0.15415'	0.5703125

Table S13. Overall power and power within each band at each electrode location, responders vs. nonresponders at 12 months.

Band	Responder	Nonresponder	*p*
Fp2 Theta	3.8657 +/- 11.3529	4.9465 +/- 12.7688	0.9047619048
Fp2 Alpha	3.104 +/- 8.3952	4.0413 +/- 9.7008	0.9047619048
Fp2 Beta	2.596 +/- 5.9794	3.1583 +/- 6.3109	1
Fp2 Delta	4.3727 +/- 13.546	5.9244 +/- 15.46	0.9047619048
Fp2 Gamma	1.3789 +/- 0.34836	0.85194 +/- 1.0288	0.5555555556
Fp2 Overall	-2.2322 +/- 4.4446	-0.66245 +/- 5.2259	0.5555555556
C4 Theta	-0.34696 +/- 6.0526	8.8903 +/- 15.0659	0.5555555556
C4 Alpha	-0.1919 +/- 4.9954	7.2162 +/- 10.9923	0.4126984127
C4 Beta	0.14758 +/- 3.4796	4.5264 +/- 7.663	0.5555555556
C4 Delta	-0.5074 +/- 6.8441	10.1087 +/- 18.4151	0.5555555556
C4 Gamma	1.2924 +/- 0.37144	0.24782 +/- 1.1533	0.1904761905
C4 Overall	-1.3641 +/- 6.0767	2.3196 +/- 4.4499	0.4126984127
T4 Theta	-0.60906 +/- 6.3015	2.6268 +/- 10.8113	0.5555555556

T4 Alpha	-0.37583 +/- 4.99	2.1475 +/- 8.5243	0.5555555556
T4 Beta	0.16218 +/- 3.5644	1.3171 +/- 7.2477	0.5555555556
T4 Delta	-0.98192 +/- 7.3908	2.9434 +/- 13.3124	0.5555555556
T4 Gamma	1.0889 +/- 0.24756	0.7237 +/- 0.74393	0.7301587302
T4 Overall	-1.8243 +/- 6.6757	2.7367 +/- 3.5957	0.4126984127
O2 Theta	10.9437 +/- 18.4557	-10.9494 +/- 15.3236	0.06349206349
O2 Alpha	8.5616 +/- 13.9509	-8.5268 +/- 12.2066	0.06349206349
O2 Beta	6.4333 +/- 10.0916	-6.5159 +/- 9.562	0.06349206349
O2 Delta	12.1761 +/- 20.5025	-12.0872 +/- 16.4235	0.06349206349
O2 Gamma	1.0838 +/- 0.2601	0.99295 +/- 0.25663	0.5555555556
O2 Overall	-2.1098 +/- 6.5643	0.5297 +/- 4.5975	0.9047619048
Fp1 Theta	-7.3688 +/- 16.9548	-3.4871 +/- 12.9186	0.9047619048
Fp1 Alpha	-5.2204 +/- 12.6937	-2.5462 +/- 9.6418	0.9047619048
Fp1 Beta	-3.2896 +/- 8.97	-0.98705 +/- 7.3066	0.9047619048
Fp1 Delta	-9.16 +/- 20.5449	-3.4155 +/- 16.0033	0.9047619048
Fp1 Gamma	1.3407 +/- 0.48565	1.3761 +/- 0.30743	1
Fp1 Overall	-2.5223 +/- 3.4678	-1.4558 +/- 1.9731	0.7301587302
C3 Theta	-2.3408 +/- 19.5204	-0.54243 +/- 10.2505	1
C3 Alpha	-1.4812 +/- 14.7974	-0.2544 +/- 7.733	1
C3 Beta	-0.97314 +/- 10.6706	0.87095 +/- 5.804	1
C3 Delta	-2.8848 +/- 22.3382	-0.0668 +/- 12.2378	1
C3 Gamma	1.644 +/- 0.78412	1.7471 +/- 0.63414	1
C3 Overall	-2.159 +/- 4.504	3.4797 +/- 3.6792	0.1111111111
T3 Theta	8.6691 +/- 12.834	-0.4037 +/- 10.2829	0.7301587302
T3 Alpha	6.8175 +/- 9.8663	-0.024125 +/- 7.648	0.7301587302
T3 Beta	5.9278 +/- 7.8818	0.7377 +/- 6.1507	0.7301587302
T3 Delta	9.848 +/- 15.0373	-0.37955 +/- 12.7168	0.9047619048
T3 Gamma	1.6113 +/- 0.47253	1.6679 +/- 0.52993	1
T3 Overall	-0.36397 +/- 7.7151	4.7502 +/- 5.5424	0.2857142857
O1 Theta	14.8945 +/- 25.5207	-17.1496 +/- 24.1692	0.1111111111
O1 Alpha	11.2409 +/- 18.7955	-12.5911 +/- 18.3045	0.1111111111
O1 Beta	8.9828 +/- 14.4332	-9.0374 +/- 13.285	0.1111111111
O1 Delta	17.287 +/- 30.8961	-21.1198 +/- 30.156	0.1111111111
O1 Gamma	1.3979 +/- 0.36646	1.9103 +/- 1.6757	1
O1 Overall	-0.52584 +/- 7.7886	0.75636 +/- 4.971	1
R hemi Theta	3.4634 +/- 8.9092	1.3786 +/- 6.5691	0.9047619048
R hemi Alpha	2.7743 +/- 6.7552	1.2196 +/- 4.845	0.9047619048
R hemi Beta	2.3347 +/- 4.8785	0.6215 +/- 3.678	0.9047619048
R hemi Delta	3.7649 +/- 10.0688	1.7222 +/- 8.3678	0.7301587302
R hemi Gamma	1.211 +/- 0.22515	0.70408 +/- 0.58203	0.1111111111
R hemi Overall	-1.8826 +/- 5.0924	1.2309 +/- 4.3236	0.7301587302
L hemi Theta	3.4635 +/- 5.7659	-5.3956 +/- 9.3932	0.2857142857
L hemi Alpha	2.8392 +/- 4.4764	-3.854 +/- 7.1053	0.2857142857

L hemi Beta	2.662 +/- 3.3407	-2.104 +/- 5.3071	0.2857142857
L hemi Delta	3.7725 +/- 7.1742	-6.2453 +/- 11.5987	0.4126984127
L hemi Gamma	1.4984 +/- 0.3399	1.6753 +/- 0.40856	0.5555555556
L hemi Overall	-1.3928 +/- 5.7513	1.8826 +/- 3.852	0.4126984127
Overall Theta	3.4633 +/- 6.0901	-2.0086 +/- 4.7534	0.1904761905
Overall Alpha	2.8068 +/- 4.7558	-1.3172 +/- 3.6127	0.1904761905
Overall Beta	2.4984 +/- 3.443	-0.74125 +/- 2.8251	0.1111111111
Overall Delta	3.7687 +/- 6.852	-2.2615 +/- 5.8483	0.2857142857
Overall Gamma	1.3547 +/- 0.24039	1.1897 +/- 0.35202	0.5555555556
Overall Overall	-1.6377 +/- 5.2614	1.5567 +/- 3.8295	0.7301587302

Table S14. Hemispheric asymmetry, overall and within each band, responders vs. nonresponders at 12 months.

Band	Responder	Nonresponder	*p*
Frontal Theta	-11.2346 +/- 26.1697	-8.4338 +/- 18.4022	0.9047619048
Frontal Alpha	-8.3244 +/- 19.3682	-6.5874 +/- 13.8776	0.9047619048
Frontal Beta	-5.8856 +/- 13.7635	-4.1454 +/- 9.5249	0.9047619048
Frontal Delta	-13.5328 +/- 31.4547	-9.3395 +/- 22.4963	0.7301587302
Frontal Gamma	-0.03826 +/- 0.52048	0.52416 +/- 0.91088	0.7301587302
Frontal Overall	-0.2901 +/- 1.5279	-0.79327 +/- 3.7533	1
Parietal Theta	-1.9938 +/- 16.026	-9.4331 +/- 24.2982	0.7301587302
Parietal Alpha	-1.2894 +/- 12.2404	-7.4707 +/- 17.9548	0.5555555556
Parietal Beta	-1.1207 +/- 9.1235	-3.6556 +/- 12.8365	0.7301587302
Parietal Delta	-2.3774 +/- 18.1687	-10.1756 +/- 29.4525	0.7301587302
Parietal Gamma	0.35159 +/- 0.49374	1.4993 +/- 1.7108	0.9047619048
Parietal Overall	-0.79492 +/- 3.6136	1.1601 +/- 3.6654	0.4126984127
Temporal Theta	9.2781 +/- 9.1192	-3.0304 +/- 19.152	0.2857142857
Temporal Alpha	7.1934 +/- 7.1135	-2.1717 +/- 14.7077	0.2857142857
Temporal Beta	5.7657 +/- 5.7858	-0.57939 +/- 12.2108	0.2857142857
Temporal Delta	10.8299 +/- 10.7306	-3.3228 +/- 23.7859	0.2857142857
Temporal Gamma	0.52237 +/- 0.51869	0.94422 +/- 1.264	1
Temporal Overall	1.4604 +/- 6.9764	2.0134 +/- 3.7288	0.4126984127
Occipital Theta	3.9507 +/- 29.0482	-6.2003 +/- 29.42	0.9047619048
Occipital Alpha	2.6794 +/- 21.3236	-4.0643 +/- 22.5128	1
Occipital Beta	2.5494 +/- 16.3704	-2.5218 +/- 16.6737	0.7301587302
Occipital Delta	5.1111 +/- 34.6302	-9.0326 +/- 34.9297	0.9047619048
Occipital Gamma	0.31409 +/- 0.47707	0.91735 +/- 1.7768	0.9047619048
Occipital Overall	1.584 +/- 4.6037	0.22664 +/- 1.9977	0.7301587302
Overall Theta	0.00076 +/- 35.0701	-27.097 +/- 52.5198	0.5555555556
Overall Alpha	0.25882 +/- 25.5736	-20.2942 +/- 39.1326	0.5555555556
Overall Beta	1.3089 +/- 18.976	-10.902 +/- 28.6945	0.5555555556
Overall Delta	0.03098 +/- 43.4317	-31.8702 +/- 66.0044	0.5555555556
Overall Gamma	1.1498 +/- 1.2729	3.885 +/- 2.8727	0.2857142857

Overall Overall	1.9592 +/- 10.8017	2.6071 +/- 11.5941	0.9047619048

Table S15. Inter-electrode coherence measures in the overall spectrum, responders vs. nonresponders at 12 months.

Band	Responder	Nonresponder	*p*
Fp2-C4 Theta	0.39189 +/- 0.20646	0.60747 +/- 0.40075	0.5555555556
Fp2-O2 Theta	0.43168 +/- 0.26609	0.53265 +/- 0.31766	0.7301587302
Fp2-T4 Theta	0.2564 +/- 0.089608	0.38307 +/- 0.32492	0.7301587302
Fp2-Fp1 Theta	0.34868 +/- 0.23182	0.47323 +/- 0.33044	0.5555555556
Fp2-C3 Theta	0.2517 +/- 0.10461	0.28692 +/- 0.12025	0.9047619048
Fp2-T3 Theta	0.21684 +/- 0.08327	0.29378 +/- 0.11993	0.4126984127
Fp2-O1 Theta	0.2555 +/- 0.1474	0.38688 +/- 0.20081	0.2857142857
C4-O2 Theta	0.39538 +/- 0.17656	0.41707 +/- 0.31601	0.9047619048
C4-T4 Theta	0.3817 +/- 0.28597	0.57069 +/- 0.38223	0.5555555556
C4-Fp1 Theta	0.2776 +/- 0.029325	0.36149 +/- 0.050619	0.03174603175
C4-C3 Theta	0.25935 +/- 0.040367	0.27828 +/- 0.17915	0.4126984127
C4-T3 Theta	0.23034 +/- 0.035664	0.23375 +/- 0.16069	0.9047619048
C4-O1 Theta	0.22474 +/- 0.031261	0.25968 +/- 0.13657	0.2857142857
O2-T4 Theta	0.47536 +/- 0.25877	0.3372 +/- 0.10966	0.4126984127
O2-Fp1 Theta	0.34829 +/- 0.17177	0.40371 +/- 0.19789	0.7301587302
O2-C3 Theta	0.29595 +/- 0.12489	0.25754 +/- 0.10075	0.9047619048
O2-T3 Theta	0.32423 +/- 0.097771	0.29314 +/- 0.11876	0.7301587302
O2-O1 Theta	0.35755 +/- 0.14161	0.41593 +/- 0.2825	1
T4-Fp1 Theta	0.21336 +/- 0.065209	0.29858 +/- 0.089746	0.1904761905
T4-C3 Theta	0.26677 +/- 0.087389	0.19454 +/- 0.16083	0.4126984127
T4-T3 Theta	0.29191 +/- 0.089614	0.23502 +/- 0.1688	0.9047619048
T4-O1 Theta	0.30234 +/- 0.14474	0.25861 +/- 0.15355	0.9047619048
Fp1-C3 Theta	0.36596 +/- 0.19502	0.38594 +/- 0.12689	1
Fp1-T3 Theta	0.28806 +/- 0.096195	0.36147 +/- 0.11969	0.2857142857
Fp1-O1 Theta	0.35047 +/- 0.23796	0.47441 +/- 0.28475	0.7301587302
C3-T3 Theta	0.3114 +/- 0.10374	0.49072 +/- 0.2414	0.06349206349
C3-O1 Theta	0.28603 +/- 0.063134	0.279 +/- 0.17962	0.9047619048
T3-O1 Theta	0.49489 +/- 0.1805	0.51894 +/- 0.33881	0.5555555556
Fp2-C4 Alpha	0.36625 +/- 0.18193	0.56095 +/- 0.43392	0.5555555556
Fp2-O2 Alpha	0.42029 +/- 0.25684	0.49609 +/- 0.27989	0.7301587302
Fp2-T4 Alpha	0.27659 +/- 0.09946	0.3236 +/- 0.26422	0.9047619048
Fp2-Fp1 Alpha	0.31441 +/- 0.20616	0.45209 +/- 0.28102	0.5555555556
Fp2-C3 Alpha	0.2099 +/- 0.066998	0.27031 +/- 0.15892	0.7301587302
Fp2-T3 Alpha	0.21585 +/- 0.083784	0.30288 +/- 0.15038	0.4126984127
Fp2-O1 Alpha	0.25831 +/- 0.13838	0.36366 +/- 0.1084	0.2857142857
C4-O2 Alpha	0.39172 +/- 0.19328	0.41789 +/- 0.30091	0.9047619048
C4-T4 Alpha	0.38881 +/- 0.3103	0.48532 +/- 0.39903	1
C4-Fp1 Alpha	0.24815 +/- 0.049935	0.32227 +/- 0.15108	0.5555555556

C4-C3 Alpha	0.23441 +/- 0.039579	0.27303 +/- 0.18997	0.4126984127
C4-T3 Alpha	0.20247 +/- 0.041688	0.25257 +/- 0.20077	0.9047619048
C4-O1 Alpha	0.22556 +/- 0.066994	0.27851 +/- 0.17778	0.7301587302
O2-T4 Alpha	0.4057 +/- 0.28415	0.38523 +/- 0.16048	0.9047619048
O2-Fp1 Alpha	0.31989 +/- 0.13481	0.35175 +/- 0.11578	0.7301587302
O2-C3 Alpha	0.27142 +/- 0.07489	0.24495 +/- 0.13186	0.7301587302
O2-T3 Alpha	0.28312 +/- 0.095894	0.29869 +/- 0.12124	0.7301587302
O2-O1 Alpha	0.37274 +/- 0.15969	0.40653 +/- 0.19401	0.9047619048
T4-Fp1 Alpha	0.23006 +/- 0.047833	0.27368 +/- 0.1679	1
T4-C3 Alpha	0.24659 +/- 0.064602	0.19632 +/- 0.19604	0.2857142857
T4-T3 Alpha	0.281 +/- 0.12437	0.25189 +/- 0.19865	0.9047619048
T4-O1 Alpha	0.33072 +/- 0.19631	0.28415 +/- 0.19988	0.9047619048
Fp1-C3 Alpha	0.3953 +/- 0.15485	0.41209 +/- 0.1463	0.7301587302
Fp1-T3 Alpha	0.26897 +/- 0.069314	0.41613 +/- 0.15398	0.1904761905
Fp1-O1 Alpha	0.33132 +/- 0.21731	0.41537 +/- 0.26305	0.9047619048
C3-T3 Alpha	0.35761 +/- 0.11242	0.51971 +/- 0.26805	0.5555555556
C3-O1 Alpha	0.3251 +/- 0.12057	0.30131 +/- 0.22635	0.7301587302
T3-O1 Alpha	0.52983 +/- 0.23275	0.54297 +/- 0.35921	0.9047619048
Fp2-C4 Beta	0.3223 +/- 0.10495	0.53282 +/- 0.38936	0.9047619048
Fp2-O2 Beta	0.39226 +/- 0.19332	0.46458 +/- 0.28989	0.9047619048
Fp2-T4 Beta	0.30891 +/- 0.16446	0.22819 +/- 0.1499	0.1904761905
Fp2-Fp1 Beta	0.35547 +/- 0.1765	0.37015 +/- 0.14657	0.9047619048
Fp2-C3 Beta	0.25681 +/- 0.10861	0.2727 +/- 0.16821	0.9047619048
Fp2-T3 Beta	0.29328 +/- 0.12637	0.28742 +/- 0.17773	1
Fp2-O1 Beta	0.3062 +/- 0.14688	0.31725 +/- 0.15555	1
C4-O2 Beta	0.37726 +/- 0.1685	0.41018 +/- 0.29112	1
C4-T4 Beta	0.44055 +/- 0.30728	0.45719 +/- 0.35653	0.9047619048
C4-Fp1 Beta	0.26304 +/- 0.070382	0.31956 +/- 0.12691	0.7301587302
C4-C3 Beta	0.23081 +/- 0.089332	0.28066 +/- 0.18901	0.5555555556
C4-T3 Beta	0.22386 +/- 0.059671	0.25343 +/- 0.19342	0.7301587302
C4-O1 Beta	0.25784 +/- 0.083178	0.28559 +/- 0.17468	1
O2-T4 Beta	0.41007 +/- 0.26326	0.4014 +/- 0.1653	0.9047619048
O2-Fp1 Beta	0.30325 +/- 0.13055	0.29575 +/- 0.13138	1
O2-C3 Beta	0.32203 +/- 0.12309	0.25791 +/- 0.13733	0.7301587302
O2-T3 Beta	0.35973 +/- 0.18253	0.28418 +/- 0.12141	0.7301587302
O2-O1 Beta	0.41423 +/- 0.17017	0.38011 +/- 0.11121	0.9047619048
T4-Fp1 Beta	0.27183 +/- 0.10335	0.22378 +/- 0.136	0.4126984127
T4-C3 Beta	0.28494 +/- 0.11859	0.18523 +/- 0.19167	0.4126984127
T4-T3 Beta	0.30965 +/- 0.1582	0.22134 +/- 0.18614	0.5555555556
T4-O1 Beta	0.33997 +/- 0.22492	0.24491 +/- 0.16904	0.4126984127
Fp1-C3 Beta	0.36891 +/- 0.14928	0.48132 +/- 0.13391	0.2857142857
Fp1-T3 Beta	0.3052 +/- 0.14246	0.4526 +/- 0.19256	0.1904761905
Fp1-O1 Beta	0.38928 +/- 0.19453	0.40465 +/- 0.27978	1

C3-T3 Beta	0.3713 +/- 0.18892	0.64923 +/- 0.19763	0.1111111111
C3-O1 Beta	0.33426 +/- 0.10967	0.35166 +/- 0.23244	0.9047619048
T3-O1 Beta	0.53416 +/- 0.23493	0.50958 +/- 0.40652	0.9047619048
Fp2-C4 Delta	0.39825 +/- 0.19215	0.58568 +/- 0.34139	0.4126984127
Fp2-O2 Delta	0.44475 +/- 0.27564	0.4923 +/- 0.22808	0.7301587302
Fp2-T4 Delta	0.24166 +/- 0.1002	0.41087 +/- 0.36572	0.5555555556
Fp2-Fp1 Delta	0.34554 +/- 0.2018	0.46035 +/- 0.30367	0.5555555556
Fp2-C3 Delta	0.22195 +/- 0.076651	0.28388 +/- 0.11008	0.4126984127
Fp2-T3 Delta	0.20314 +/- 0.084518	0.30269 +/- 0.10509	0.1904761905
Fp2-O1 Delta	0.26448 +/- 0.13318	0.38584 +/- 0.20665	0.4126984127
C4-O2 Delta	0.39198 +/- 0.18474	0.43058 +/- 0.14122	0.9047619048
C4-T4 Delta	0.31888 +/- 0.27356	0.69981 +/- 0.34142	0.1904761905
C4-Fp1 Delta	0.31084 +/- 0.079638	0.32504 +/- 0.096868	0.5555555556
C4-C3 Delta	0.24692 +/- 0.044864	0.26381 +/- 0.16688	0.4126984127
C4-T3 Delta	0.22235 +/- 0.065277	0.27068 +/- 0.17625	0.4126984127
C4-O1 Delta	0.22983 +/- 0.076457	0.30877 +/- 0.18105	0.5555555556
O2-T4 Delta	0.43481 +/- 0.28221	0.3596 +/- 0.09603	1
O2-Fp1 Delta	0.36625 +/- 0.15722	0.38745 +/- 0.23295	1
O2-C3 Delta	0.25367 +/- 0.046926	0.25437 +/- 0.095729	0.7301587302
O2-T3 Delta	0.24775 +/- 0.081256	0.28847 +/- 0.11286	0.5555555556
O2-O1 Delta	0.29125 +/- 0.14746	0.38505 +/- 0.23596	0.7301587302
T4-Fp1 Delta	0.21259 +/- 0.085294	0.27482 +/- 0.095729	0.4126984127
T4-C3 Delta	0.2622 +/- 0.08417	0.19151 +/- 0.12979	0.5555555556
T4-T3 Delta	0.23531 +/- 0.091507	0.22411 +/- 0.17654	0.9047619048
T4-O1 Delta	0.32813 +/- 0.15812	0.25332 +/- 0.17607	0.4126984127
Fp1-C3 Delta	0.33243 +/- 0.15891	0.38712 +/- 0.06413	0.5555555556
Fp1-T3 Delta	0.24717 +/- 0.091762	0.32216 +/- 0.081127	0.2857142857
Fp1-O1 Delta	0.4155 +/- 0.25055	0.39497 +/- 0.28594	1
C3-T3 Delta	0.29516 +/- 0.097694	0.50791 +/- 0.24669	0.2857142857
C3-O1 Delta	0.29824 +/- 0.099571	0.27907 +/- 0.10411	0.9047619048
T3-O1 Delta	0.41248 +/- 0.14233	0.46009 +/- 0.3201	0.7301587302
Fp2-C4 Gamma	0.31542 +/- 0.11528	0.61934 +/- 0.34567	0.2857142857
Fp2-O2 Gamma	0.35902 +/- 0.14631	0.4322 +/- 0.27486	0.9047619048
Fp2-T4 Gamma	0.34358 +/- 0.25162	0.25682 +/- 0.12665	0.7301587302
Fp2-Fp1 Gamma	0.42766 +/- 0.27252	0.33531 +/- 0.17359	0.7301587302
Fp2-C3 Gamma	0.33024 +/- 0.19229	0.291 +/- 0.15771	1
Fp2-T3 Gamma	0.33716 +/- 0.20653	0.28937 +/- 0.17015	0.9047619048
Fp2-O1 Gamma	0.37175 +/- 0.24694	0.30075 +/- 0.16812	1
C4-O2 Gamma	0.38104 +/- 0.16435	0.42233 +/- 0.23993	0.9047619048
C4-T4 Gamma	0.4496 +/- 0.30536	0.50761 +/- 0.33928	0.7301587302
C4-Fp1 Gamma	0.28689 +/- 0.092076	0.34313 +/- 0.15522	0.5555555556
C4-C3 Gamma	0.28662 +/- 0.14303	0.28977 +/- 0.18853	0.9047619048
C4-T3 Gamma	0.26079 +/- 0.089085	0.27574 +/- 0.19114	0.7301587302

C4-O1 Gamma	0.26634 +/- 0.093518	0.30632 +/- 0.15242	0.5555555556
O2-T4 Gamma	0.39952 +/- 0.22959	0.37093 +/- 0.10553	0.9047619048
O2-Fp1 Gamma	0.30669 +/- 0.10708	0.28917 +/- 0.15016	0.7301587302
O2-C3 Gamma	0.28336 +/- 0.063979	0.26372 +/- 0.13434	1
O2-T3 Gamma	0.28583 +/- 0.073525	0.27378 +/- 0.12698	1
O2-O1 Gamma	0.37192 +/- 0.17522	0.39912 +/- 0.022795	0.2857142857
T4-Fp1 Gamma	0.30667 +/- 0.23849	0.21915 +/- 0.13121	0.4126984127
T4-C3 Gamma	0.31642 +/- 0.2135	0.17931 +/- 0.14915	0.4126984127
T4-T3 Gamma	0.32307 +/- 0.21318	0.19698 +/- 0.14633	0.4126984127
T4-O1 Gamma	0.35248 +/- 0.27978	0.23462 +/- 0.116	0.5555555556
Fp1-C3 Gamma	0.34452 +/- 0.19501	0.62028 +/- 0.18821	0.1111111111
Fp1-T3 Gamma	0.36943 +/- 0.19638	0.5347 +/- 0.12464	0.2857142857
Fp1-O1 Gamma	0.44776 +/- 0.23673	0.45728 +/- 0.26944	1
C3-T3 Gamma	0.35793 +/- 0.1924	0.70889 +/- 0.21735	0.06349206349
C3-O1 Gamma	0.4019 +/- 0.22381	0.40437 +/- 0.23373	1
T3-O1 Gamma	0.5506 +/- 0.27072	0.47882 +/- 0.40023	0.7301587302
Fp2-C4 Overall	0.34574 +/- 0.13748	0.59586 +/- 0.32355	0.2857142857
Fp2-O2 Overall	0.39222 +/- 0.14556	0.42571 +/- 0.22189	0.9047619048
Fp2-T4 Overall	0.3087 +/- 0.16226	0.28476 +/- 0.16416	0.5555555556
Fp2-Fp1 Overall	0.43131 +/- 0.23594	0.36016 +/- 0.15118	0.5555555556
Fp2-C3 Overall	0.31802 +/- 0.14325	0.26409 +/- 0.13956	0.5555555556
Fp2-T3 Overall	0.2951 +/- 0.13719	0.30393 +/- 0.1766	1
Fp2-O1 Overall	0.35577 +/- 0.14651	0.33758 +/- 0.15097	1
C4-O2 Overall	0.36815 +/- 0.14191	0.40397 +/- 0.2176	0.9047619048
C4-T4 Overall	0.4088 +/- 0.25615	0.51003 +/- 0.32043	0.5555555556
C4-Fp1 Overall	0.30741 +/- 0.089919	0.32432 +/- 0.094344	0.9047619048
C4-C3 Overall	0.2928 +/- 0.11894	0.28358 +/- 0.18257	0.9047619048
C4-T3 Overall	0.26209 +/- 0.050105	0.28948 +/- 0.21297	1
C4-O1 Overall	0.28157 +/- 0.10003	0.32113 +/- 0.18097	1
O2-T4 Overall	0.3861 +/- 0.18172	0.3955 +/- 0.10332	0.7301587302
O2-Fp1 Overall	0.32354 +/- 0.091721	0.29058 +/- 0.10855	0.5555555556
O2-C3 Overall	0.28341 +/- 0.064559	0.24607 +/- 0.1406	0.4126984127
O2-T3 Overall	0.26635 +/- 0.064945	0.29039 +/- 0.10723	0.5555555556
O2-O1 Overall	0.37938 +/- 0.15976	0.40948 +/- 0.10116	0.9047619048
T4-Fp1 Overall	0.28423 +/- 0.11831	0.21769 +/- 0.10768	0.1904761905
T4-C3 Overall	0.28707 +/- 0.12892	0.19252 +/- 0.18098	0.1904761905
T4-T3 Overall	0.28602 +/- 0.15153	0.23179 +/- 0.17148	0.7301587302
T4-O1 Overall	0.31785 +/- 0.20383	0.26901 +/- 0.14193	0.9047619048
Fp1-C3 Overall	0.3626 +/- 0.11902	0.47435 +/- 0.11492	0.1904761905
Fp1-T3 Overall	0.33896 +/- 0.11182	0.41485 +/- 0.092341	0.2857142857
Fp1-O1 Overall	0.41154 +/- 0.15087	0.3931 +/- 0.2233	0.7301587302
C3-T3 Overall	0.32141 +/- 0.12297	0.60526 +/- 0.20605	0.06349206349
C3-O1 Overall	0.34928 +/- 0.10939	0.36326 +/- 0.2078	0.9047619048

T3-O1 Overall	0.52538 +/- 0.25706	0.49262 +/- 0.37964	0.7301587302

Table S16. Overall power and power within each band at each electrode location, responders vs. nonresponders at baseline.

Band	Responder	Nonresponder	*p*
Fp2 Theta	-88.7476 +/- 190.7184	3.9453 +/- 17.6653	0.4126984127
Fp2 Alpha	-77.7953 +/- 168.5764	3.9215 +/- 14.8228	0.4126984127
Fp2 Beta	-38.0153 +/- 81.9785	3.9051 +/- 12.3888	0.4126984127
Fp2 Delta	-97.8747 +/- 209.1688	5.8223 +/- 23.9937	0.5555555556
Fp2 Gamma	22.3515 +/- 46.4822	2.7708 +/- 2.1126	0.5555555556
Fp2 Overall	-0.84427 +/- 5.2972	-3.2852 +/- 3.0321	0.5555555556
C4 Theta	-26.5576 +/- 48.3814	21.4697 +/- 40.8932	0.06349206349
C4 Alpha	-22.9883 +/- 43.7827	16.0712 +/- 30.2145	0.06349206349
C4 Beta	-13.5931 +/- 26.0609	10.0012 +/- 17.6985	0.06349206349
C4 Delta	-30.137 +/- 53.9055	28.9849 +/- 56.0683	0.06349206349
C4 Gamma	6.4729 +/- 10.703	-3.0102 +/- 9.0175	0.2857142857
C4 Overall	-0.040596 +/- 5.5718	2.3646 +/- 5.1388	0.5555555556
T4 Theta	-5.063 +/- 8.3401	4.2408 +/- 8.3158	0.1904761905
T4 Alpha	-3.8213 +/- 6.8428	3.2828 +/- 6.4217	0.1904761905
T4 Beta	-1.7951 +/- 4.2483	2.31 +/- 4.5299	0.1904761905
T4 Delta	-6.1018 +/- 9.5156	5.1732 +/- 11.109	0.1904761905
T4 Gamma	2.0586 +/- 1.6713	0.49218 +/- 1.4633	0.4126984127
T4 Overall	2.9783 +/- 8.2406	3.1827 +/- 5.0326	0.9047619048
O2 Theta	-13.6977 +/- 27.9036	-12.5931 +/- 22.6078	1
O2 Alpha	-11.4641 +/- 24.2671	-9.8694 +/- 18.2305	1
O2 Beta	-6.0958 +/- 14.0333	-6.7426 +/- 13.2438	1
O2 Delta	-15.7504 +/- 31.2809	-14.9049 +/- 24.9415	1
O2 Gamma	4.3303 +/- 6.4476	1.6145 +/- 0.76846	0.7301587302
O2 Overall	2.7693 +/- 8.1794	-0.81734 +/- 3.2665	0.7301587302
Fp1 Theta	-11.2994 +/- 13.4592	-0.41362 +/- 6.3451	0.4126984127
Fp1 Alpha	-9.2018 +/- 11.3046	-0.30883 +/- 4.581	0.4126984127
Fp1 Beta	-4.9644 +/- 6.8791	0.38994 +/- 3.7894	0.2857142857
Fp1 Delta	-13.4669 +/- 16.3458	-0.52907 +/- 8.1962	0.4126984127
Fp1 Gamma	3.4147 +/- 3.004	1.742 +/- 0.353	0.9047619048
Fp1 Overall	-0.61286 +/- 5.0461	-3.1759 +/- 3.6473	0.4126984127
C3 Theta	18.2081 +/- 26.872	-1.087 +/- 5.8534	0.1904761905
C3 Alpha	13.6415 +/- 18.7374	-0.56052 +/- 4.3479	0.1904761905
C3 Beta	12.0817 +/- 18.0205	0.15598 +/- 3.0157	0.1904761905
C3 Delta	21.6449 +/- 32.2861	-1.9108 +/- 7.7547	0.1111111111
C3 Gamma	0.69776 +/- 2.0797	1.6641 +/- 0.94985	0.9047619048
C3 Overall	1.0985 +/- 2.7138	1.8305 +/- 6.0948	0.9047619048
T3 Theta	37.9362 +/- 72.159	-0.51708 +/- 5.2215	0.1904761905
T3 Alpha	29.9524 +/- 56.2856	-0.26395 +/- 3.9702	0.06349206349

T3 Beta	20.6991 +/- 37.576	0.21443 +/- 2.9813	0.1111111111
T3 Delta	46.6102 +/- 89.9466	-1.0041 +/- 6.7677	0.1904761905
T3 Gamma	-2.8622 +/- 8.0081	1.4583 +/- 0.78852	0.2857142857
T3 Overall	4.8702 +/- 3.8621	1.54 +/- 5.9038	0.4126984127
O1 Theta	4.2785 +/- 8.7783	-11.5345 +/- 22.2641	0.2857142857
O1 Alpha	3.6955 +/- 6.7074	-9.3346 +/- 18.2673	0.2857142857
O1 Beta	2.6512 +/- 4.2908	-6.0724 +/- 12.7109	0.2857142857
O1 Delta	4.7182 +/- 10.3752	-13.0014 +/- 23.9549	0.2857142857
O1 Gamma	0.5171 +/- 1.4734	1.7927 +/- 0.6065	0.1904761905
O1 Overall	3.4584 +/- 4.3841	-1.2789 +/- 3.9847	0.1904761905
R hemi Theta	-33.5166 +/- 68.2927	4.2658 +/- 15.2843	0.5555555556
R hemi Alpha	-29.0172 +/- 60.48	3.3516 +/- 11.8124	0.5555555556
R hemi Beta	-14.8749 +/- 31.1816	2.3683 +/- 8.147	0.5555555556
R hemi Delta	-37.4651 +/- 75.2733	6.2686 +/- 19.488	0.5555555556
R hemi Gamma	8.8033 +/- 16.3189	0.46683 +/- 2.6443	0.5555555556
R hemi Overall	1.2156 +/- 6.6095	0.36117 +/- 3.6588	0.9047619048
L hemi Theta	12.281 +/- 18.5603	-3.3881 +/- 6.5282	0.1111111111
L hemi Alpha	9.5219 +/- 14.421	-2.617 +/- 5.0944	0.1111111111
L hemi Beta	7.6169 +/- 9.7474	-1.328 +/- 3.8348	0.1111111111
L hemi Delta	14.8763 +/- 22.9223	-4.1113 +/- 7.7481	0.1111111111
L hemi Gamma	0.44186 +/- 2.3108	1.6643 +/- 0.58362	0.7301587302
L hemi Overall	2.2036 +/- 3.6055	-0.27108 +/- 4.5798	0.5555555556
Overall Theta	-10.6177 +/- 38.1871	0.43887 +/- 9.6024	0.9047619048
Overall Alpha	-9.7476 +/- 33.332	0.36722 +/- 7.616	0.9047619048
Overall Beta	-3.629 +/- 17.9351	0.5202 +/- 5.6204	0.9047619048
Overall Delta	-11.2947 +/- 42.7563	1.0787 +/- 11.6307	0.9047619048
Overall Gamma	4.6227 +/- 8.4839	1.0655 +/- 1.055	0.9047619048
Overall Overall	1.7096 +/- 5.0134	0.04505 +/- 4.058	0.7301587302

Table S17. Hemispheric asymmetry, overall and within each band, responders vs. nonresponders at baseline.

Band	Responder	Nonresponder	*p*
Frontal Theta	77.4469 +/- 185.0394	-4.3589 +/- 11.3392	0.7301587302
Frontal Alpha	68.5919 +/- 162.9033	-4.2303 +/- 10.2445	0.7301587302
Frontal Beta	33.0507 +/- 80.4063	-3.5151 +/- 8.6116	0.5555555556
Frontal Delta	84.4094 +/- 203.637	-6.3513 +/- 15.8759	0.7301587302
Frontal Gamma	-18.9366 +/- 43.9486	-1.0288 +/- 1.9867	0.9047619048
Frontal Overall	0.2314 +/- 2.8832	0.10931 +/- 1.6368	0.9047619048
Parietal Theta	44.765 +/- 54.8546	-22.5568 +/- 45.3511	0.06349206349
Parietal Alpha	36.6279 +/- 47.4097	-16.6317 +/- 33.4354	0.06349206349
Parietal Beta	25.6749 +/- 30.4103	-9.8452 +/- 19.4355	0.06349206349
Parietal Delta	51.7812 +/- 61.7961	-30.8945 +/- 62.2689	0.06349206349
Parietal Gamma	-5.7752 +/- 12.0453	4.6744 +/- 9.9149	0.9047619048

Parietal Overall	1.1391 +/- 3.9803	-0.53408 +/- 2.7845	0.5555555556
Temporal Theta	42.9989 +/- 73.0282	-4.7579 +/- 10.4373	0.1111111111
Temporal Alpha	33.7728 +/- 56.9712	-3.5469 +/- 7.6957	0.1111111111
Temporal Beta	22.4942 +/- 38.3981	-2.0955 +/- 4.5559	0.1111111111
Temporal Delta	52.7117 +/- 91.3961	-6.1772 +/- 13.8316	0.1904761905
Temporal Gamma	-4.9208 +/- 7.8382	0.96613 +/- 2.2438	0.4126984127
Temporal Overall	1.8919 +/- 5.2844	-1.6428 +/- 2.7397	0.2857142857
Occipital Theta	17.9763 +/- 31.2241	1.0586 +/- 1.4327	1
Occipital Alpha	15.1597 +/- 26.9177	0.53477 +/- 1.1043	0.7301587302
Occipital Beta	8.7471 +/- 15.0519	0.67039 +/- 0.69932	1
Occipital Delta	20.4684 +/- 34.9186	1.9033 +/- 1.9334	1
Occipital Gamma	-3.8132 +/- 7.4016	0.17817 +/- 0.71164	0.5555555556
Occipital Overall	0.68929 +/- 4.6354	-0.46153 +/- 0.81194	0.4126984127
Overall Theta	183.1888 +/- 258.7206	-30.6151 +/- 54.1997	0.06349206349
Overall Alpha	154.1549 +/- 229.344	-23.8741 +/- 39.7904	0.06349206349
Overall Beta	89.9679 +/- 116.4796	-14.7856 +/- 23.9345	0.06349206349
Overall Delta	209.3699 +/- 284.8495	-41.5203 +/- 73.6026	0.06349206349
Overall Gamma	-33.4463 +/- 63.9263	4.7898 +/- 12.7829	0.4126984127
Overall Overall	3.9517 +/- 14.3307	-2.529 +/- 6.7589	0.4126984127

Table S18. Inter-electrode coherence measures in the overall spectrum, responders vs. nonresponders at baseline.

Band	Responder	Nonresponder	*p*
Fp2-C4 Theta	0.51282 +/- 0.18869	0.24746 +/- 0.079216	0.1111111111
Fp2-O2 Theta	0.48515 +/- 0.082119	0.38032 +/- 0.13825	0.4126984127
Fp2-T4 Theta	0.44966 +/- 0.17677	0.28998 +/- 0.087546	0.1904761905
Fp2-Fp1 Theta	0.54116 +/- 0.16124	0.36314 +/- 0.12923	0.1111111111
Fp2-C3 Theta	0.39194 +/- 0.11657	0.28099 +/- 0.17733	0.2857142857
Fp2-T3 Theta	0.35963 +/- 0.18336	0.38372 +/- 0.1347	0.9047619048
Fp2-O1 Theta	0.34403 +/- 0.17319	0.37646 +/- 0.097559	0.9047619048
C4-O2 Theta	0.48201 +/- 0.25418	0.26637 +/- 0.11498	0.1111111111
C4-T4 Theta	0.4165 +/- 0.19704	0.55592 +/- 0.37815	0.7301587302
C4-Fp1 Theta	0.48349 +/- 0.15311	0.24661 +/- 0.07528	0.06349206349
C4-C3 Theta	0.44953 +/- 0.20552	0.28984 +/- 0.10937	0.1904761905
C4-T3 Theta	0.33298 +/- 0.16198	0.27204 +/- 0.078962	0.7301587302
C4-O1 Theta	0.33779 +/- 0.15795	0.28662 +/- 0.097447	0.9047619048
O2-T4 Theta	0.63253 +/- 0.22533	0.40084 +/- 0.30522	0.2857142857
O2-Fp1 Theta	0.43456 +/- 0.16047	0.34524 +/- 0.13462	0.5555555556
O2-C3 Theta	0.40407 +/- 0.20865	0.28476 +/- 0.12332	0.1904761905
O2-T3 Theta	0.31517 +/- 0.17515	0.35083 +/- 0.14503	0.7301587302
O2-O1 Theta	0.3473 +/- 0.14412	0.42943 +/- 0.14162	0.5555555556
T4-Fp1 Theta	0.42257 +/- 0.16603	0.30873 +/- 0.14208	0.2857142857
T4-C3 Theta	0.36826 +/- 0.1655	0.29096 +/- 0.13548	0.4126984127

T4-T3 Theta	0.34489 +/- 0.17842	0.34037 +/- 0.097892	0.7301587302
T4-O1 Theta	0.31505 +/- 0.17346	0.35036 +/- 0.15918	0.7301587302
Fp1-C3 Theta	0.48283 +/- 0.11404	0.45379 +/- 0.30584	0.9047619048
Fp1-T3 Theta	0.42111 +/- 0.25939	0.39749 +/- 0.12561	0.9047619048
Fp1-O1 Theta	0.45286 +/- 0.22995	0.45428 +/- 0.073792	0.9047619048
C3-T3 Theta	0.295 +/- 0.16708	0.50268 +/- 0.31158	0.4126984127
C3-O1 Theta	0.35445 +/- 0.14385	0.39399 +/- 0.20214	0.9047619048
T3-O1 Theta	0.62977 +/- 0.22173	0.47455 +/- 0.18105	0.9047619048
Fp2-C4 Alpha	0.43368 +/- 0.18652	0.27451 +/- 0.07985	0.2857142857
Fp2-O2 Alpha	0.38415 +/- 0.1176	0.4028 +/- 0.181	0.9047619048
Fp2-T4 Alpha	0.36509 +/- 0.12063	0.30747 +/- 0.10223	0.7301587302
Fp2-Fp1 Alpha	0.45069 +/- 0.1892	0.45899 +/- 0.19651	1
Fp2-C3 Alpha	0.37862 +/- 0.12974	0.40536 +/- 0.18662	0.7301587302
Fp2-T3 Alpha	0.35046 +/- 0.17591	0.45428 +/- 0.16665	0.2857142857
Fp2-O1 Alpha	0.3443 +/- 0.1812	0.42202 +/- 0.14432	0.4126984127
C4-O2 Alpha	0.43136 +/- 0.21116	0.27806 +/- 0.1004	0.4126984127
C4-T4 Alpha	0.32152 +/- 0.11725	0.56205 +/- 0.3604	0.4126984127
C4-Fp1 Alpha	0.4571 +/- 0.12856	0.30469 +/- 0.10182	0.1111111111
C4-C3 Alpha	0.46439 +/- 0.17408	0.32939 +/- 0.1168	0.4126984127
C4-T3 Alpha	0.34374 +/- 0.19024	0.29066 +/- 0.10614	1
C4-O1 Alpha	0.36973 +/- 0.1842	0.3053 +/- 0.11055	0.7301587302
O2-T4 Alpha	0.52012 +/- 0.18899	0.44537 +/- 0.28831	0.5555555556
O2-Fp1 Alpha	0.3822 +/- 0.10483	0.4495 +/- 0.16919	0.5555555556
O2-C3 Alpha	0.36889 +/- 0.18614	0.44335 +/- 0.111	0.4126984127
O2-T3 Alpha	0.32065 +/- 0.15743	0.40519 +/- 0.1581	0.5555555556
O2-O1 Alpha	0.39585 +/- 0.13226	0.51436 +/- 0.11921	0.2857142857
T4-Fp1 Alpha	0.34081 +/- 0.1201	0.40557 +/- 0.17119	0.7301587302
T4-C3 Alpha	0.32717 +/- 0.10163	0.37146 +/- 0.11926	0.9047619048
T4-T3 Alpha	0.31138 +/- 0.15814	0.37104 +/- 0.12729	0.5555555556
T4-O1 Alpha	0.30272 +/- 0.11058	0.3969 +/- 0.16155	0.4126984127
Fp1-C3 Alpha	0.48494 +/- 0.058442	0.56799 +/- 0.21426	0.5555555556
Fp1-T3 Alpha	0.40934 +/- 0.25317	0.49563 +/- 0.14488	0.4126984127
Fp1-O1 Alpha	0.48801 +/- 0.25109	0.62824 +/- 0.066318	0.2857142857
C3-T3 Alpha	0.26975 +/- 0.13853	0.55846 +/- 0.33026	0.06349206349
C3-O1 Alpha	0.4016 +/- 0.13932	0.53582 +/- 0.14137	0.1111111111
T3-O1 Alpha	0.5744 +/- 0.25128	0.51619 +/- 0.19646	0.7301587302
Fp2-C4 Beta	0.44352 +/- 0.19844	0.34299 +/- 0.11235	0.5555555556
Fp2-O2 Beta	0.39514 +/- 0.11889	0.44825 +/- 0.21087	0.7301587302
Fp2-T4 Beta	0.29826 +/- 0.10105	0.33695 +/- 0.1194	0.2857142857
Fp2-Fp1 Beta	0.37658 +/- 0.13543	0.45688 +/- 0.18162	0.5555555556
Fp2-C3 Beta	0.36927 +/- 0.16364	0.38172 +/- 0.15561	0.9047619048
Fp2-T3 Beta	0.28667 +/- 0.13814	0.42116 +/- 0.15072	0.4126984127
Fp2-O1 Beta	0.31049 +/- 0.12428	0.45271 +/- 0.18078	0.2857142857

C4-O2 Beta	0.42814 +/- 0.17289	0.26045 +/- 0.063325	0.1904761905
C4-T4 Beta	0.30893 +/- 0.10732	0.55748 +/- 0.33206	0.4126984127
C4-Fp1 Beta	0.40206 +/- 0.14613	0.3194 +/- 0.13577	0.7301587302
C4-C3 Beta	0.45924 +/- 0.15561	0.32499 +/- 0.13723	0.1904761905
C4-T3 Beta	0.27975 +/- 0.12043	0.30646 +/- 0.11774	0.7301587302
C4-O1 Beta	0.3241 +/- 0.11295	0.32235 +/- 0.13075	0.9047619048
O2-T4 Beta	0.47593 +/- 0.21538	0.44836 +/- 0.24142	0.7301587302
O2-Fp1 Beta	0.35491 +/- 0.081732	0.55446 +/- 0.10316	0.01587301587
O2-C3 Beta	0.36214 +/- 0.17383	0.48994 +/- 0.058556	0.1904761905
O2-T3 Beta	0.31996 +/- 0.12167	0.42214 +/- 0.10412	0.4126984127
O2-O1 Beta	0.3652 +/- 0.13259	0.6264 +/- 0.041948	0.01587301587
T4-Fp1 Beta	0.31479 +/- 0.14617	0.4682 +/- 0.17664	0.2857142857
T4-C3 Beta	0.28702 +/- 0.0924	0.39463 +/- 0.12486	0.2857142857
T4-T3 Beta	0.30876 +/- 0.14749	0.39771 +/- 0.13751	0.4126984127
T4-O1 Beta	0.29184 +/- 0.13191	0.45264 +/- 0.16743	0.1111111111
Fp1-C3 Beta	0.44044 +/- 0.1305	0.64528 +/- 0.21992	0.4126984127
Fp1-T3 Beta	0.33234 +/- 0.15718	0.51269 +/- 0.19405	0.2857142857
Fp1-O1 Beta	0.48919 +/- 0.16674	0.7505 +/- 0.092113	0.1111111111
C3-T3 Beta	0.26187 +/- 0.12649	0.50091 +/- 0.32353	0.1111111111
C3-O1 Beta	0.38392 +/- 0.12937	0.6236 +/- 0.1967	0.1111111111
T3-O1 Beta	0.46772 +/- 0.2185	0.51282 +/- 0.22514	1
Fp2-C4 Delta	0.49519 +/- 0.20818	0.25081 +/- 0.10857	0.1904761905
Fp2-O2 Delta	0.38972 +/- 0.13235	0.31749 +/- 0.092702	0.5555555556
Fp2-T4 Delta	0.40246 +/- 0.20909	0.30131 +/- 0.092662	0.5555555556
Fp2-Fp1 Delta	0.40987 +/- 0.20164	0.34107 +/- 0.07803	1
Fp2-C3 Delta	0.33638 +/- 0.058941	0.30125 +/- 0.14193	0.4126984127
Fp2-T3 Delta	0.32243 +/- 0.13777	0.38312 +/- 0.10068	0.5555555556
Fp2-O1 Delta	0.31977 +/- 0.10497	0.34174 +/- 0.1087	0.7301587302
C4-O2 Delta	0.36827 +/- 0.2344	0.23755 +/- 0.076212	0.4126984127
C4-T4 Delta	0.4341 +/- 0.23796	0.57446 +/- 0.32214	0.5555555556
C4-Fp1 Delta	0.38257 +/- 0.15344	0.24542 +/- 0.095208	0.1904761905
C4-C3 Delta	0.37036 +/- 0.10035	0.27346 +/- 0.09969	0.4126984127
C4-T3 Delta	0.29464 +/- 0.12066	0.25691 +/- 0.071215	0.7301587302
C4-O1 Delta	0.31546 +/- 0.091814	0.23443 +/- 0.074507	0.1111111111
O2-T4 Delta	0.58649 +/- 0.24378	0.38612 +/- 0.29641	0.2857142857
O2-Fp1 Delta	0.28603 +/- 0.13889	0.29645 +/- 0.14574	1
O2-C3 Delta	0.30781 +/- 0.12823	0.28703 +/- 0.11353	0.5555555556
O2-T3 Delta	0.23034 +/- 0.076135	0.31529 +/- 0.13318	0.2857142857
O2-O1 Delta	0.26591 +/- 0.079462	0.38614 +/- 0.15031	0.4126984127
T4-Fp1 Delta	0.31119 +/- 0.11437	0.26755 +/- 0.12737	0.4126984127
T4-C3 Delta	0.29773 +/- 0.11952	0.2662 +/- 0.10986	0.7301587302
T4-T3 Delta	0.29494 +/- 0.09115	0.28524 +/- 0.10629	0.9047619048
T4-O1 Delta	0.27567 +/- 0.085823	0.28298 +/- 0.15928	0.9047619048

Fp1-C3 Delta	0.40378 +/- 0.12796	0.45963 +/- 0.26901	0.9047619048
Fp1-T3 Delta	0.32507 +/- 0.15448	0.39632 +/- 0.1352	0.5555555556
Fp1-O1 Delta	0.42482 +/- 0.13158	0.37075 +/- 0.13767	0.9047619048
C3-T3 Delta	0.27909 +/- 0.14089	0.4931 +/- 0.34428	0.2857142857
C3-O1 Delta	0.34965 +/- 0.12732	0.36913 +/- 0.15827	0.9047619048
T3-O1 Delta	0.54534 +/- 0.21871	0.40714 +/- 0.1522	0.7301587302
Fp2-C4 Gamma	0.42578 +/- 0.23464	0.46634 +/- 0.23149	0.9047619048
Fp2-O2 Gamma	0.35362 +/- 0.15283	0.51696 +/- 0.21213	0.2857142857
Fp2-T4 Gamma	0.27868 +/- 0.12866	0.35884 +/- 0.14227	0.2857142857
Fp2-Fp1 Gamma	0.33614 +/- 0.15553	0.43845 +/- 0.22511	0.5555555556
Fp2-C3 Gamma	0.33329 +/- 0.13332	0.46764 +/- 0.21884	0.2857142857
Fp2-T3 Gamma	0.25128 +/- 0.14062	0.45869 +/- 0.22674	0.2857142857
Fp2-O1 Gamma	0.25261 +/- 0.13564	0.44519 +/- 0.20192	0.2857142857
C4-O2 Gamma	0.38011 +/- 0.1654	0.31621 +/- 0.11594	0.7301587302
C4-T4 Gamma	0.30661 +/- 0.12469	0.52324 +/- 0.3423	0.4126984127
C4-Fp1 Gamma	0.33958 +/- 0.17449	0.28702 +/- 0.14655	0.7301587302
C4-C3 Gamma	0.38879 +/- 0.157	0.33133 +/- 0.12064	0.7301587302
C4-T3 Gamma	0.26981 +/- 0.12773	0.30223 +/- 0.11422	0.9047619048
C4-O1 Gamma	0.27424 +/- 0.12865	0.2948 +/- 0.14068	1
O2-T4 Gamma	0.49939 +/- 0.24394	0.42929 +/- 0.21053	0.5555555556
O2-Fp1 Gamma	0.30242 +/- 0.090945	0.51841 +/- 0.047835	0.01587301587
O2-C3 Gamma	0.35136 +/- 0.12306	0.52013 +/- 0.13525	0.1904761905
O2-T3 Gamma	0.28621 +/- 0.11125	0.50641 +/- 0.18332	0.1904761905
O2-O1 Gamma	0.31241 +/- 0.093972	0.62947 +/- 0.10296	0.01587301587
T4-Fp1 Gamma	0.31512 +/- 0.10946	0.38578 +/- 0.10365	0.2857142857
T4-C3 Gamma	0.29512 +/- 0.072966	0.376 +/- 0.085589	0.2857142857
T4-T3 Gamma	0.31183 +/- 0.12636	0.4427 +/- 0.11606	0.1904761905
T4-O1 Gamma	0.2965 +/- 0.10126	0.38245 +/- 0.13796	0.5555555556
Fp1-C3 Gamma	0.41827 +/- 0.15398	0.60691 +/- 0.22268	0.4126984127
Fp1-T3 Gamma	0.26566 +/- 0.15356	0.51877 +/- 0.18167	0.1111111111
Fp1-O1 Gamma	0.36435 +/- 0.097116	0.61956 +/- 0.11947	0.03174603175
C3-T3 Gamma	0.3102 +/- 0.1328	0.61605 +/- 0.31893	0.1111111111
C3-O1 Gamma	0.35497 +/- 0.15331	0.55266 +/- 0.11568	0.1904761905
T3-O1 Gamma	0.45018 +/- 0.23723	0.47719 +/- 0.19973	0.9047619048
Fp2-C4 Overall	0.4443 +/- 0.20127	0.36427 +/- 0.17111	0.9047619048
Fp2-O2 Overall	0.36039 +/- 0.084047	0.4537 +/- 0.12848	0.4126984127
Fp2-T4 Overall	0.32982 +/- 0.095746	0.29388 +/- 0.10473	0.9047619048
Fp2-Fp1 Overall	0.38863 +/- 0.090776	0.44521 +/- 0.11825	0.4126984127
Fp2-C3 Overall	0.34057 +/- 0.093367	0.41362 +/- 0.12998	0.5555555556
Fp2-T3 Overall	0.30251 +/- 0.12197	0.43622 +/- 0.14193	0.1904761905
Fp2-O1 Overall	0.30077 +/- 0.10751	0.42225 +/- 0.085964	0.1111111111
C4-O2 Overall	0.40038 +/- 0.15891	0.26704 +/- 0.072157	0.1904761905
C4-T4 Overall	0.33611 +/- 0.12663	0.54918 +/- 0.35303	0.5555555556

C4-Fp1 Overall	0.40849 +/- 0.085805	0.24766 +/- 0.070981	0.06349206349
C4-C3 Overall	0.42448 +/- 0.15693	0.31536 +/- 0.097107	0.1904761905
C4-T3 Overall	0.32252 +/- 0.10907	0.28437 +/- 0.11026	0.7301587302
C4-O1 Overall	0.31173 +/- 0.1216	0.26438 +/- 0.074244	1
O2-T4 Overall	0.54329 +/- 0.2094	0.40049 +/- 0.18726	0.5555555556
O2-Fp1 Overall	0.33127 +/- 0.079242	0.49623 +/- 0.094799	0.03174603175
O2-C3 Overall	0.35902 +/- 0.15582	0.4597 +/- 0.061318	0.1904761905
O2-T3 Overall	0.31211 +/- 0.08785	0.46234 +/- 0.12245	0.1111111111
O2-O1 Overall	0.3555 +/- 0.13115	0.61072 +/- 0.053838	0.01587301587
T4-Fp1 Overall	0.34019 +/- 0.13297	0.32514 +/- 0.098092	1
T4-C3 Overall	0.32286 +/- 0.10581	0.35086 +/- 0.12241	0.7301587302
T4-T3 Overall	0.31054 +/- 0.10131	0.41141 +/- 0.15008	0.4126984127
T4-O1 Overall	0.32935 +/- 0.08642	0.36827 +/- 0.1143	0.9047619048
Fp1-C3 Overall	0.46226 +/- 0.15745	0.54666 +/- 0.23652	0.7301587302
Fp1-T3 Overall	0.31556 +/- 0.094544	0.46908 +/- 0.13863	0.1111111111
Fp1-O1 Overall	0.3986 +/- 0.10559	0.56135 +/- 0.12503	0.1111111111
C3-T3 Overall	0.33989 +/- 0.13487	0.59331 +/- 0.32634	0.2857142857
C3-O1 Overall	0.36607 +/- 0.12722	0.5248 +/- 0.1618	0.1904761905
T3-O1 Overall	0.45151 +/- 0.20726	0.52273 +/- 0.13885	0.5555555556
